# Towards personalized immersive virtual reality neurorehabilitation: a human-centered design

**DOI:** 10.1186/s12984-024-01489-5

**Published:** 2025-01-20

**Authors:** Salvatore Luca Cucinella, Joost C. F. de Winter, Erik Grauwmeijer, Marc Evers, Laura Marchal-Crespo

**Affiliations:** 1 Dept. of Cognitive Robotics, TU Delft, Delft, Netherlands; 2 Dept. of Rehabilitation Medicine, Erasmus MC, Rotterdam, Netherlands; 3 Dept. of Rehabilitation Medicine, Rijndam Rehabilitation Centre, Rotterdam, Netherlands

**Keywords:** Acquired brain injury, Stroke, Virtual reality, Head-mounted displays, Neurorehabilitation, Human-centered design, Co-creation

## Abstract

**Background:**

Head-mounted displays can be used to offer personalized immersive virtual reality (IVR) training for patients who have suffered an Acquired Brain Injury (ABI) by tailoring the complexity of visual and auditory stimuli to the patient’s cognitive capabilities. However, it is still an open question how these virtual environments should be designed.

**Methods:**

We used a human-centered design approach to help define the characteristics of suitable virtual training environments for ABI patients. We conducted (i) observations, (ii) interviews with eleven neurorehabilitation experts, and (iii) an online questionnaire with 24 neurorehabilitation experts to examine how therapists modify current training environments to promote patients’ recovery in conventional sensorimotor neurorehabilitation settings. Finally, (iv) we involved eight neurorehabilitation experts in a participatory design workshop to co-create examples of IVR training environments.

**Results:**

Five phases of the recovery process (Screening, Planning, Training, Reflecting, and Discharging) and six key themes describing the characteristics of suitable (physical) training environments (Specific, Meaningful, Versatile, Educational, Safe, and Supportive) were identified. The experts agreed that modulating the number of elements (e.g., objects, people) or distractions (e.g., background noise) in the physical training environment enables therapists to provide their patients with suitable conditions to execute functional tasks. Additionally, the experts highlighted the importance of developing IVR training environments that are meaningful and realistic.

**Conclusions:**

Through consultations with neurorehabilitation experts, we gained insights into how therapists adjust physical training environments to promote the execution of functional sensorimotor tasks in patients with diverse cognitive capabilities. Their recommendations on how to modulate and make IVR environments meaningful may contribute to increased motivation and skill transfer. Future studies on IVR-based neurorehabilitation should involve patients themselves.

**Supplementary Information:**

The online version contains supplementary material available at 10.1186/s12984-024-01489-5.

## Introduction

Stroke and traumatic brain injury (TBI; e.g., after motor vehicle accidents and sports injuries) are the two most common causes of acquired brain injury (ABI), with stroke affecting approximately 12.2 million people each year [1], making it the second-leading cause of death worldwide [2]. Almost 50% of stroke survivors suffer from motor deficits, and 14% to 42% suffer—also or only—from cognitive impairments [3], limiting their functional autonomy. These patients require rehabilitation [4], and rapid access to treatment improves recovery, especially during the first weeks after an ABI [5, 6].

Neurorehabilitation is a multidisciplinary program essential for regaining functional independence [5]. In rehabilitation centers, patients engage in therapy sessions, which may include speech therapy [7, 8], activities to improve mobility [9], cognitive rehabilitation [10], pain and stress management [11, 12], and emotional support [13], among others. They also receive assistance with activities of daily living, such as walking and personal hygiene, as the inability to perform these tasks may lead to unsafe conditions and poor quality of life [14]. Sensorimotor training exercises are a key component of the neurorehabilitation process to support patients in improving their sensory integration and motor coordination [15]. Specifically, physical and occupational therapists engage patients in highly repetitive high-dosage [16], task-specific [17], and personalized [18] motor tasks to promote their recovery.

In assisting patients in achieving their recovery goals, multidisciplinary teams, including physical and occupational therapists, ensure that patients have stimulating experiences without becoming overwhelmed. Previous research suggests that brain-injured patients who engage in more cognitively, socially, and physically stimulating activities may be more physically active and mentally healthier [19]. Training experiences are usually tailored to each individual patient, as the effect of sensory stimulation on ABI patients can range from experiencing sensory stimuli as abnormal (sensory hyposensitivity) to overwhelming (sensory hypersensitivity) [20]. While several clinical guidelines for stroke provide recommendations on how to reduce the cognitive demands on people with impaired attention after a stroke (e.g., [21]), they do not describe the types of elements or distractors neurorehabilitation experts should modulate. Overall, while these findings indicate the importance of providing adequate stimulation while avoiding excessive complexity, there is a lack of clarity about the influence of training environments on the recovery process.

In daily practice, sensorimotor training is usually conducted either individually or in groups within environments designed for practicing task-specific and context-specific training. In some rehabilitation centers, healthcare staff employ environmental enrichment techniques. Environmental enrichment is an intervention designed to create housing environments that encourage physical (motor and sensory), cognitive, and social activities [22, 23]. Different environmental settings include gyms, corridors, or stairs, and outdoor spaces such as buses and supermarkets. However, such environments can be too demanding, as patients may have to deal simultaneously with several stimuli. For instance, in a meal preparation activity, patients might have to cook in a kitchen surrounded by people talking and moving around them, all while paying attention to not burning the food.

In practice, although physical and occupational therapists strive to keep patients motivated while performing repetitive and intensive exercises to promote maximal recovery, the training environments may not always be adequately stimulating, and the training tasks may be too complex or too simple for the patient. The advent of new technology, such as immersive virtual reality (IVR) using Head-Mounted Displays (HMD), allows for the manipulation of the virtual training environment to provide more suitable training experiences. IVR is a form of human–computer interface in which users are immersed and can interact with computer-generated three-dimensional (3D) virtual environments, allowing for high levels of control of stimulation [24]. IVR can add value to conventional therapy [25, 26] by ensuring that patients receive a personalized level of stimulation during training [27]. IVR has been shown to lead to improved patient outcomes, motivation, and engagement [28, 29]. For instance, in upper-limb rehabilitation, after playing games on IVR, participants with chronic stroke showed improvements in upper extremity activity capacity [30]. Similarly, in gait and balance training, IVR increased motivation and performance (i.e., increased walking speed) of both healthy participants and those with multiple sclerosis and stroke [31]. In cognitive rehabilitation, IVR has been shown to improve cognitive functions such as memory and attention [32].

Despite advances in the use of IVR for training purposes, it is still an open question how these virtual environments should be designed. A frequent assumption is that virtual environments should resemble real-life environments, as a realistic environment can increase acceptance and user experience [33, 34], improve the transfer of learning to the real world [35], and support targeted action training (e.g., removing cups from cabinets in real kitchen environments) [36]. However, as in conventional therapy, the creation of virtual environments that neither over- nor under-stimulate but adequately challenge users should be taken into consideration. This has been shown to be relevant in different contexts, including motor learning [37] and neurorehabilitation [38, 39], as well as in studies that report the benefits of gradual exposure to stimuli for patients’ training communication skills [40] and overcoming phobias (e.g., fear of public speaking) [41]. There is also evidence that *gamified* virtual environments could improve motor recovery in acute stroke patients, e.g., by providing extrinsic feedback (e.g., game score, changing colors of targets and sounds) on their performance during the execution of a task [42]. Therefore, ABI patients might benefit from training in immersive virtual environments that are realistic, provide feedback, and assist with the gradual desensitization to stimuli in daily life, as done in desensitization treatments [20].

The aim of this work was to define, together with neurorehabilitation experts, the characteristics of conventional physical training environments for ABI patients with different levels of cognitive abilities to guide the development of personalized IVR training environments. We mainly focused on physical and occupational therapy but also involved other healthcare professionals, such as speech therapists, rehabilitation physicians, nurses, neuropsychologists, and psychologists, as physical and occupational therapists work in collaboration with them [43]. Experts were included in this study because they have extensive knowledge of therapy design and best practices, exhibit a thorough understanding of patient needs, and are able to assess the feasibility of new neurorehabilitation solutions. Patients were not included at this stage due to their sometimes complex needs, as well as safety and ethical concerns. However, future studies should include patients to validate our findings with respect to usability, motivation, and efficacy. The current work focused on subacute inpatient neurorehabilitation of ABI patients with limited upper extremity functioning. Patients in the subacute phase may benefit from IVR as, in this stage, the brain is more plastic [44]. When added to standard care, IVR can help improve the recovery of functional capabilities, as it can offer patients ways to do high-intensity, highly repetitive, and engaging exercises, sometimes not achievable in conventional rehabilitation therapies [25, 45].

We employed a Human-Centered Design (HCD) approach [46]. Previous studies that employed similar approaches to develop immersive and semi-immersive virtual training environments for neurorehabilitation have explored various areas such as lower-limb rehabilitation [47], upper-limb rehabilitation [48], communication rehabilitation [49, 50], and dementia [51]. While these studies offer valuable design recommendations for effective VR solutions (e.g., providing patients with realistic simulated experiences, quantifying patient progress, and challenging patients at appropriate levels to avoid stagnation due to overexertion or under-challenge), they do not provide specific guidance on manipulating the complexity of virtual training environments to tailor them to patients’ cognitive capabilities.

Our work, conducted at Dutch rehabilitation centers, explores the perspectives of neurorehabilitation experts on the characteristics of effective physical and IVR environments designed for patients with different cognitive capabilities. The current research focuses on the topic of personalization for the benefit of the patient, i.e., we address the question of how therapists currently adjust training conditions to the patient, and we examine how a future IVR environment should be designed to meet patients’ characteristics and capabilities, with a focus on cognitive demands. For structuring our research process, we used the Double-Diamond model [52]. According to this model, the human-centered design process is divided into four phases: Discover, Define, Develop, and Deliver [52]. This paper engages in the first three phases of the Double-Diamond, characterized by four research activities. In the discovery phase, we conducted *observations* of the recovery process of ABI patients (Study 1) to understand the steps characterizing the inpatient rehabilitation experience. Following this, we performed *semi-structured interviews* with neurorehabilitation experts (Study 2) to understand how conventional treatment is tailored to each individual’s clinical needs, recovery goals, and capabilities. In the definition phase, we verified our findings from Studies 1 and 2 by conducting an *online questionnaire* (Study 3) with 24 neurorehabilitation experts to collect their opinions on the strategies therapists seem to adopt to create suitable sensorimotor training environments. Finally, in the development phase, we conducted a *participatory design workshop* (Study 4) with neurorehabilitation experts to collect their opinions on the use of IVR-based neurorehabilitation and co-create examples of low- and high-cognitively demanding immersive virtual training environments.

## Methods

The study was approved by the Human Research Ethics Committee of the Delft University of Technology, and by the board of directors of Rijndam, who approved a request not subject to WMO research (“Wet Medisch-wetenschappelijk Onderzoek met mensen,” which translates to “Medical Research Involving Human Subjects Act” in English).

### Study 1: observations

The first author spent approximately 192 h at Rijndam Rehabilitation Centre (Rotterdam, The Netherlands) observing therapist-patient interactions and the training environments to understand the steps characterizing the inpatient rehabilitation experience. The observation period began with the researcher introducing himself and the goals of his observations to the multidisciplinary teams at the rehabilitation center. During observations, the researcher wore a white lab coat to blend in with the context.

The researcher used two observation methods, *Fly-on-the-wall* and *Shadowing*, and recorded his findings using hand notes and sketches. *Fly-on-the-wall* is a technique used to collect data and gain insight into people, environments, interactions, and objects without interfering with the users being observed [53, 54]. In the current study, observations were made while sitting in silence inside training rooms, seeing and listening to therapists and patients. *Shadowing* involves following a member of an organization closely for an extended period [55]. The researcher performed multiple observations while following ten different neurorehabilitation experts (nurses, physical therapists, occupational therapists, speech therapists, and psychologists) in daily patient interactions inside and outside the rehabilitation center. On average, each shadowing activity lasted one hour. This allowed the researcher to observe how users perform specific activities (e.g., transfer from bed to a wheelchair, brushing teeth, crossing streets) in different training environments (e.g., bathroom, bedroom, stairs, street) while interacting with one or more people (e.g., nurses, family members, other patients). During shadowing, the researcher wrote down questions, e.g., regarding the assessment tools and patients’ behavior in the environment, and asked those questions to the neurorehabilitation experts only after patients had left the session. In a few cases, the researcher was allowed to participate in the interaction, for example, by assisting the patients in getting onto a pedal exerciser bicycle or the therapists in preparing the training environment.

### Study 2: semi-structured interviews

In Study 2, the first author conducted *semi-structured interviews* [61] with eleven neurorehabilitation experts from Rijndam: three physical therapists, two occupational therapists, one speech therapist, one rehabilitation physician, one nurse, one neuropsychologist, one psychologist, and one researcher. The goal was to understand how treatment is tailored to each individual’s clinical needs, recovery goals, and capabilities.

The interview period began with the researcher introducing the nature and purpose of the activity to the multidisciplinary team at the rehabilitation center. Interested people were invited to contact the researcher. The participants included seven females and four males, with an average of 8.2 years (*SD* = 6.3) of professional experience in their field. At least 1 week before the interview, participants received a document containing detailed information about the study, including an informed consent form, which they signed before the interview started. Two interviews were conducted online, and nine in person. Interviews were conducted individually and had an average duration of 49 min. The interviews were held in English and began with a brief introduction of the activity and the experimenter, followed by open-ended questions on six topics, including: (1) Roles, responsibilities, and tasks; (2) Diagnosis, prognosis, and treatment design; (3) Therapy treatment; (4) Patients’ challenges, needs, and motivation; (5) Users’ involvement; and (6) IVR-based neurorehabilitation. The complete list of questions can be found in Additional file 1.

Audio from the interviews was recorded using a recording device and then transcribed and processed using thematic analysis [56]. The thematic analysis focused on the research question: *What strategies do therapists adopt to create a suitable training environment for patients*? Transcriptions were processed using Atlas.ti 22 and Microsoft Excel (Microsoft^®^ Excel^®^ for Microsoft 365 MSO).

### Study 3: online questionnaire

To verify the findings from Studies 1 and 2, we invited neurorehabilitation experts to participate in an *online questionnaire* (see Additional file 2, in Dutch). The questionnaire consisted of 22 statements, each representing a strategy that therapists seem to adopt to create suitable training environments for patients with different cognitive capabilities. Strategies were formulated based on data collected during the observation (Study 1) and interviews (Study 2). Eleven Dutch rehabilitation institutes were contacted via email and invited to share a link to the questionnaire with their employees. The questionnaire was offered in Dutch. Respondents expressed their degree of agreement with the statements using a Likert scale, from 1 (strongly agree) to 5 (strongly disagree). For each statement, they were invited to leave a comment about how they use the strategy in their daily work. Additionally, seven open questions were presented, through which participants could explain, for instance, what they liked the most and least about the strategy presented in each statement, how they applied them in therapy, and whether they were aware of other strategies they or their colleagues adopt to create suitable training environments. The seven open questions can be found in Additional file 2. Twenty-four respondents (18 from Rijndam and 6 from other Dutch institutions) completed the questionnaire: seven occupational therapists, six physical therapists, four speech therapists, two nurses, one rehabilitation physician, one neuropsychologist, one social worker, one researcher, and one dietitian. There were twenty-one females and three males, with an average of 14.0 years (*SD* = 10.0) of professional experience in their fields.

### Study 4: participatory design workshop

The goal of the participatory design workshop [60] was to create, with neurorehabilitation experts, examples of low- and high-cognitively demanding immersive virtual training environments for ABI patients with different levels of cognitive capability. The workshop period began with the introduction of its nature and purpose to a group of experts working at Rijndam. Interested personnel contacted the first author personally and/or digitally via email to sign up for the activity. Eight neurorehabilitation experts from Rijndam (three occupational therapists, three physical therapists, one psychologist, and one speech therapist) joined the two-hour in-person participatory design workshop. Participants included five females and three males, with an average of 13.1 years (*SD* = 10.3) of professional experience in the field of their expertise and from none to intermediate (from 10 to 100) hours of experience with virtual reality. After signing the informed consent form, participants were divided into two groups (Group A: one occupational therapist, two physical therapists, one neuropsychologist; Group B: two occupational therapists, one physical therapist, one speech therapist). Each group was assisted by a VR developer. The workshop was facilitated by the first author and held in English. However, participants were free to speak Dutch with their peers.

**Fig. 1:**
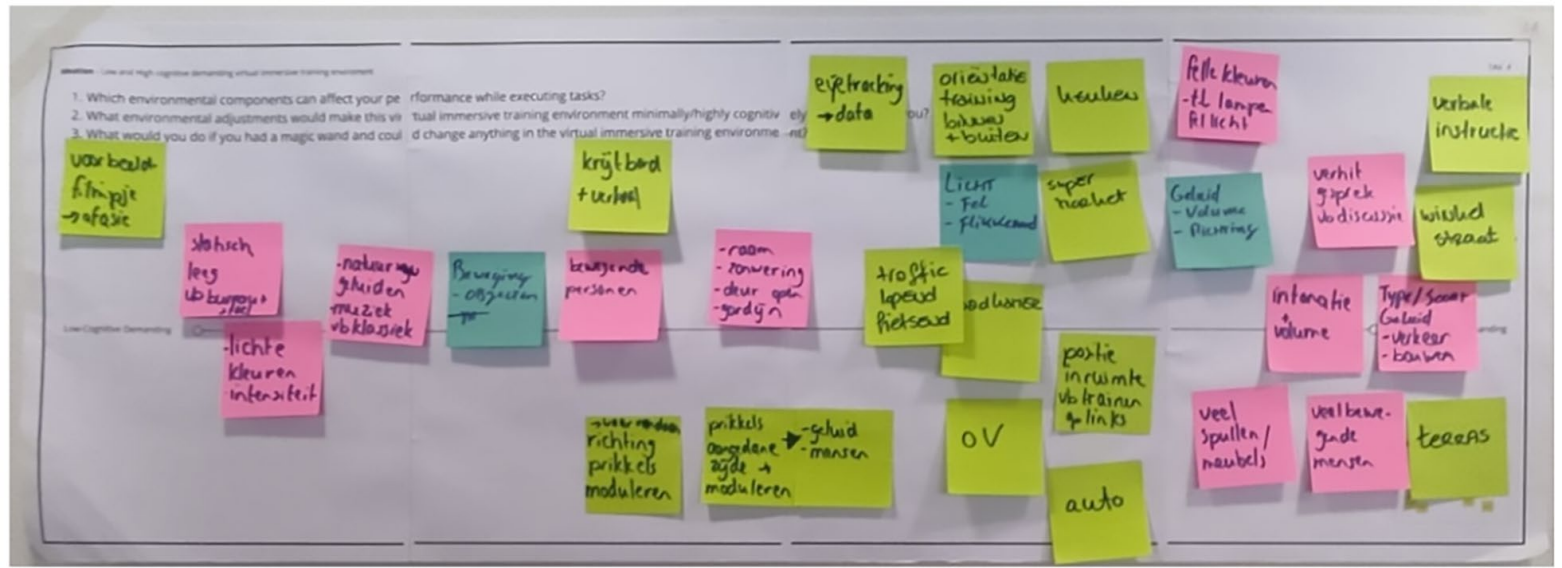
Example of  one of the paper sheets used during the ideation session. Post-its placed on top of the paper sheet report participants' ideas

The workshop consisted of a *focus group* (Activity 1; 15 min) and an *ideation session* (Activity 2; 1 h). Both activities were conducted on the same day. Between the two activities, participants took a break of 15 min. The activities were preceded by a preparation phase (15 min each) during which we provided participants with definitions of IVR, and instructions on how to use the equipment (e.g., head-mounted displays). To guide the two activities in a structured way*,* we provided each group with Post-its and four paper sheets designed by the first, second, and last author (Fig. 1). Paper sheets posed specific questions to stimulate reflection and conversation.

**Fig. 2:**
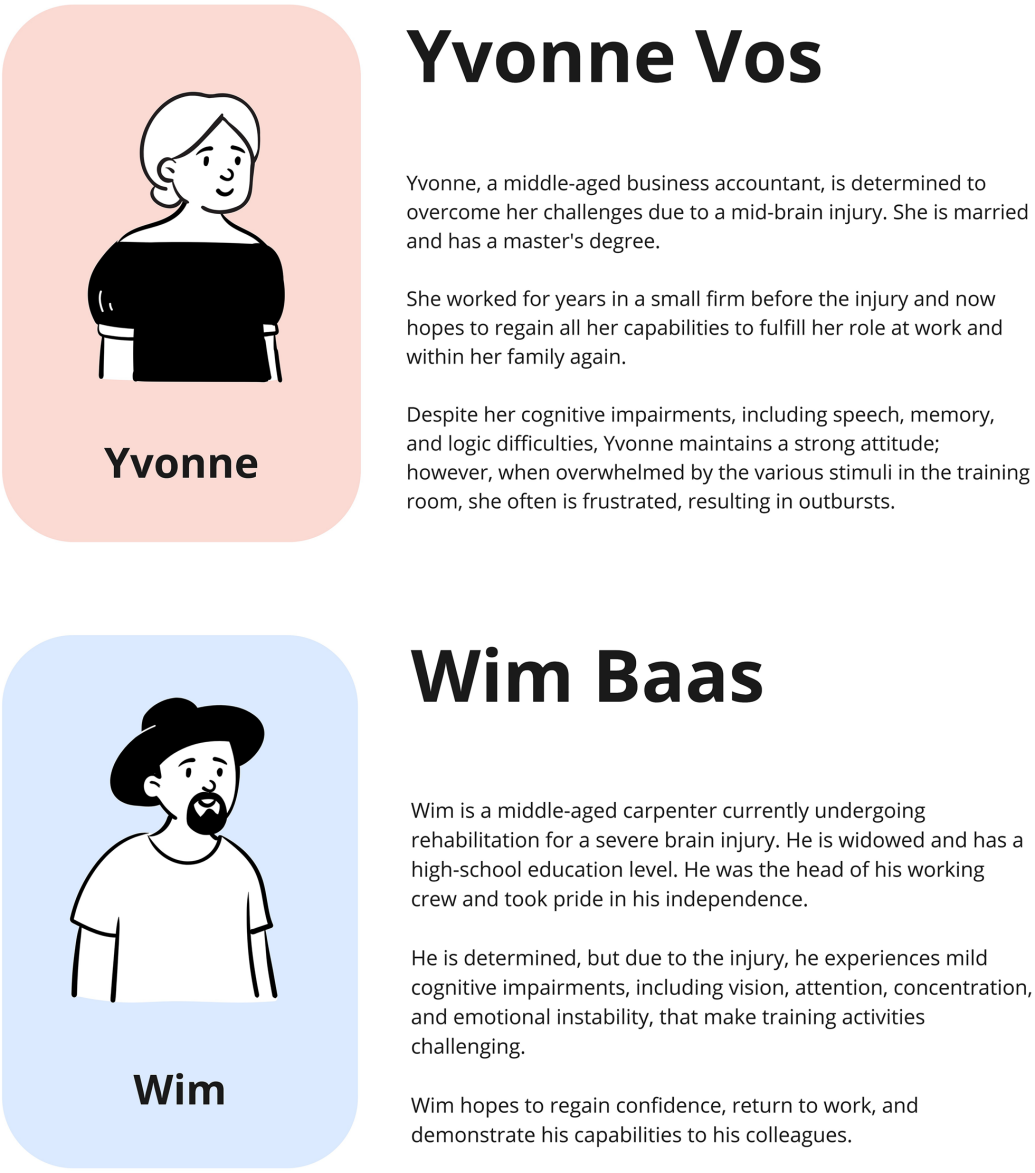
The two personas employed in Study 4. Top: Yvonne’s persona was assigned to Group A. Bottom: Wim’s persona was assigned to Group B

Each group received a *Persona,* i.e., a fictional representation of an ABI patient (Yvonne and Wim, Fig. 2). Personas are research tools that help designers empathize with end-users by reading about their needs, experiences, behaviors, and goals, and therefore, create products that would fit personas' needs [57]. The Yvonne and Wim personas were employed to facilitate that participants could experience the virtual environment from the perspective of a patient with specific motor and cognitive impairments, and, by pretending to act and think like them, propose changes that would make sense for a user with that specific clinical need. These personas were designed with a physician and a physical therapist from Rijndam, and three researchers from TU Delft, in an online workshop conducted weeks earlier. Illustrations for the Personas were designed in Miro (miro.com) using the Characters Mix and Match Icebreaker template created by Facilitator School [58].

#### Study 4-activity 1: focus group

The focus group aimed to prepare participants for the ideation session (Activity 2). Each group had three paper sheets, each with one question: (1) *What do you think about and what are the advantages and disadvantages of immersive virtual reality?;* (2) *How would you use immersive virtual reality in rehabilitation?;* and (3) *What should researchers consider when developing immersive virtual reality experiences for brain-injured patients?* These questions were designed to stimulate reflection on using IVR in neurorehabilitation. Participants were invited to read a question, verbally share their opinions using a think-aloud method [59], write their answers on Post-its, and place the Post-its on the paper sheet. They had five minutes to answer each question.

#### Study 4-activity 2: ideation session

The ideation session aimed to understand what would make an IVR training environment low- and high-cognitively demanding for ABI patients. To answer this question, we invited groups to create examples of a low- and a high-cognitively demanding immersive virtual environment using a real-time rapid prototyping technique [60]. The participants could experience an IVR environment from a first-person perspective using a HMD, and observe the effects of their changes in real time. We assigned each group a HMD (HTC Vive Pro Eye, HTC Vive, Taiwan & Valve, USA) and a monitor to display to the other team members what the user sees in the HMD. A VR developer was assigned to each group to assist with rapid prototyping. Participants in each group informed the VR developer about desired changes in the virtual environment, e.g., characteristics of objects (e.g., texture fidelity, position, size), or removal/addition of elements (e.g., furniture, sounds, avatars). The provided virtual environment was a replica of a training room at Rijndam, created prior to the activity by a VR developer with the assistance of the first author in the Unity game engine (Unity Technologies, USA—version 2021.3.24f1). The elements in the virtual environments were taken from a pre-created folder containing various Unity assets (downloaded from https://assetstore.unity.com).

**Fig. 3:**
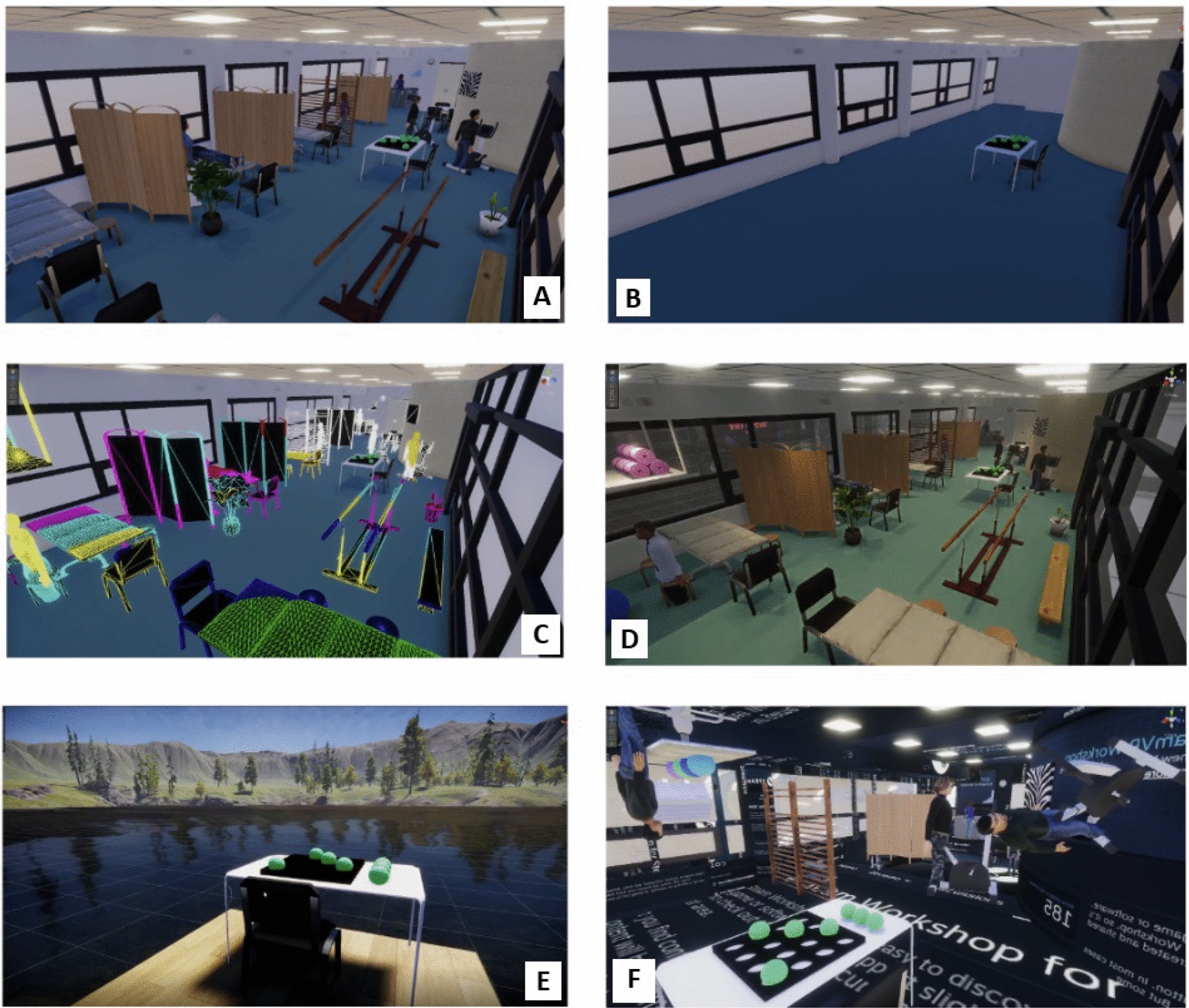
Screenshots of the six videos presented during the workshop: **A**. Normal virtual environment; **B**. Empty virtual environment; **C**. Textured virtual environment; **D**. Dynamic virtual environment; **E**. Outdoor virtual environment;** F**. Incongruent virtual environment

To show participants the potential of IVR and promote their creativity, we showed both groups six videos of different versions of the same virtual environment. The first video was the replica of the training room at Rijndam (Fig. 3A). We then showed videos of the same room but with fewer objects (Fig. 3B), and with elements with different textures (Fig. 3C). These videos were created to show participants that in IVR, it is possible to manipulate the number of objects and change their textures. Later, to show that in IVR it is possible to animate things like light, doors, windows, and avatars, we showed a video of a dynamic virtual environment (Fig. 3D). Two final videos were shown to illustrate that it is possible to re-create any environment, e.g., an outside landscape (Fig. 3E) or even incongruent scenarios, e.g., with avatars upside down and standing on the ceiling (Fig. 3F).

In a co-creation setting, each group was then asked to reflect on the characteristics of two types of virtual environments: a low- and a high-cognitively demanding one. To stimulate the reflection, we used a paper sheet containing three questions: (1) *Which environmental components can affect your performance while executing tasks?*; (2) *What environmental adjustments would make this virtual immersive training environment minimally/highly cognitively demanding for you?*; and (3) *What would you do if you had a magic wand and could change anything in the virtual immersive training environment?* Additionally, the paper sheet had a horizontal line drawn in the middle with the left extremity labeled low-cognitive demanding and the right one high-cognitive demanding. The groups were invited to read the questions on the paper sheet and the description of the assigned Persona (one Persona per group). They were also instructed to share ideas verbally using a think-aloud method [59] and write them on Post-its to place on the paper sheet. Post-its had to be placed on the left (low-cognitive demanding) or right (high-cognitive demanding) side of the line drawn on the paper sheet to discriminate between the two types of environments.

Following this first reflecting exercise, participants entered the virtual environment individually. In the virtual environment, from a first-person perspective, they sat in front of a table and had to execute a task: pretending to grasp and move some static objects from one side of the table to the other. In doing so, they had to pretend to act as the assigned Persona and propose ideas that would make that environment low- and high-cognitively demanding. VR developers did not interfere with group discussion but implemented the ideas participants agreed upon and wanted to test. At the end of this activity, the groups quickly presented and discussed their ideas.

#### Analysis

Both activities of Study 4 were audio-recorded and, during the ideation session, we also performed video-recordings of the computer monitors, thus capturing the changes that the VR developers made to the virtual environments following the participants’ recommendations. Audio recordings were manually post-processed, i.e., transcribed verbatim, and the recorded Dutch exchanges between participants were translated from Dutch to English by a Dutch-speaking student assistant.

The transcripts enabled the analysis and interpretation of the paper sheets used in both activities and the video recordings (from Activity 2). From Activity 1, analysis of the transcripts and notes on the Post-its resulted in a list of advantages and disadvantages of IVR (Question 1), examples of rehabilitation experiences to replicate in IVR (Question 2), and recommendations for the proper use of the technology with ABI patients (Question 3). From Activity 2, analysis of the transcripts, notes on the Post-its, and the video recordings resulted in a list of environmental factors affecting task performance (Question 1), adjustments for manipulating cognitive demand levels (Question 2), and examples of out-of-the-box concepts (Question 3). These findings enabled us to formulate recommendations on how IVR environments should be designed for neurorehabilitation purposes, and specifically how the IVR environment can be adjusted to offer low versus high cognitive demands.

## Results

### Study 1: observations

**Fig. 4:**
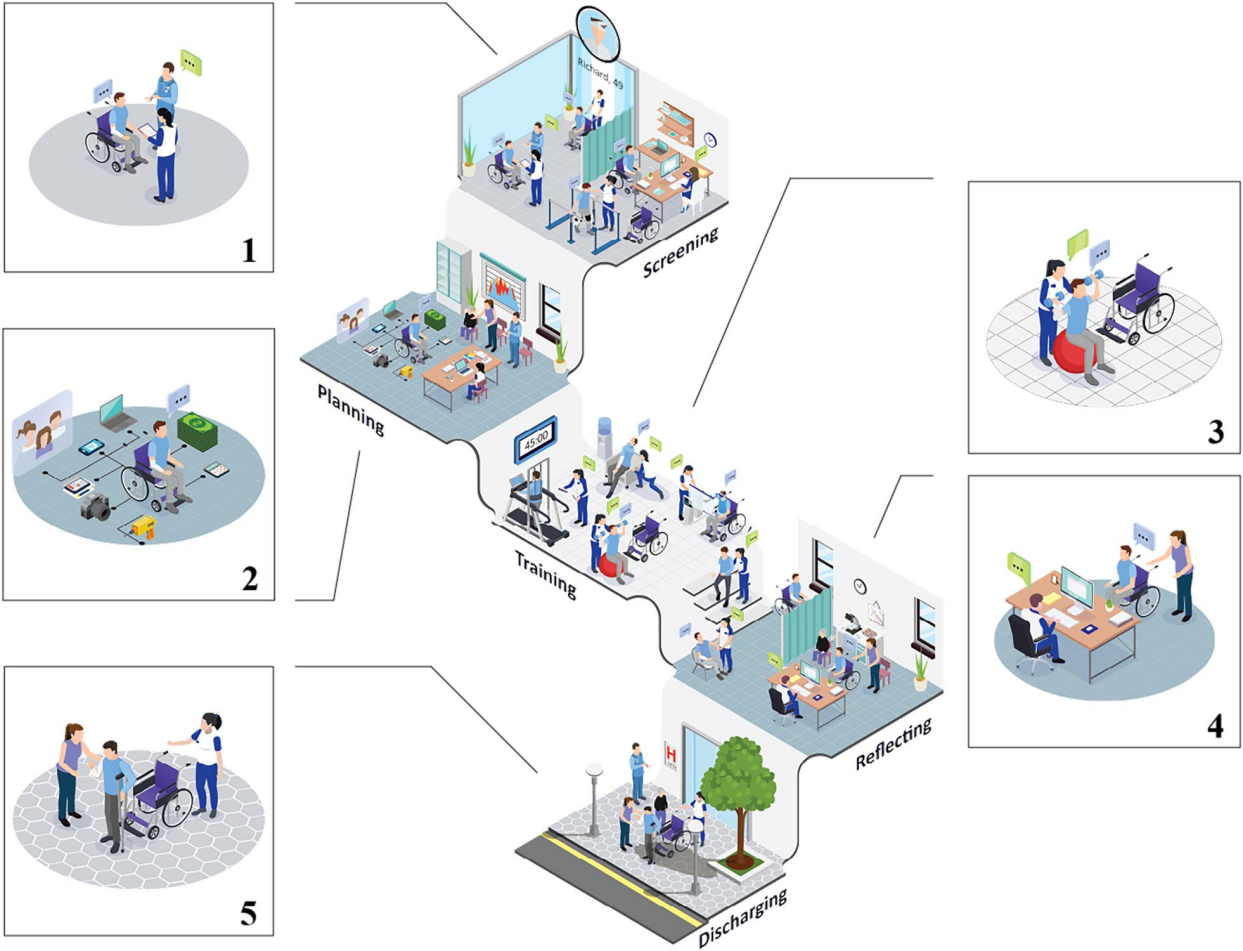
The inpatient Journey map, including the five treatment phases: (1) Screening, (2) Planning, (3) Training, (4) Reflecting, and (5) Discharging. Illustrations used in this infographic, including 'people-characters,' 'furniture,' and 'gym equipment,' were downloaded from Freepik.com [91]. The first author designed the frames for the five steps and combined and edited the illustrations accordingly to show the many patient-therapist interactions [92]

Field notes from the *Fly-on-the-wall* and *Shadowing* activities, together with learnings from the related literature [5, 62], resulted in a Journey map, which is a graphic representation of an individual’s process to accomplish a goal [63]. Our map (Fig. 4) illustrates the recovery process of an inpatient stroke survivor at a rehabilitation center. The different phases describe the environment and the interactions among therapists, family members, and patients that allow for accomplishing specific goals (e.g., collecting patient data) and the provision of tailored treatment (e.g., modifying the physical environment to meet the patient’s capabilities). Once a phase is completed, patients move to the next phase. We identified five treatment phases (Fig. 4):

*Screening:* During this phase, patient data (e.g., demographics, social, and clinical data) are collected in preparation for further clinical visits. To do so, neurorehabilitation experts use specific assessment tools, such as the Fugl-Meyer Assessment [64] for assessing patients’ motor function, and the Montreal Cognitive Assessment (MoCA) for assessing their cognitive function [65].

*Planning*: This phase involves collaboration among neurorehabilitation experts, patients, and families to define short- and long-term goals related to daily activities. Screening and planning phases are repeated after each team meeting or multidisciplinary consultation [66]. At these consultations, a team of experts who can help patients meet their specific needs during rehabilitation is involved. Team composition (e.g., physical, occupational, speech therapists, psychologists, family members) mainly depends on the patient’s clinical needs. For instance, patients suffering from visuospatial inattention (i.e., neglect) and those suffering from language impairments (i.e., aphasia) will need the support of two different specialists, either an occupational or a speech therapist, to train their lost functions. Teams meet regularly to evaluate patients’ progress and adjust therapy accordingly. Each therapist reports their evaluations and opinions on a patient’s progress to the team and proposes adjustments to the treatment plan. In consultation with physiatrists and neuropsychologists, the therapy is adjusted, and therapists help patients set more realistic recovery goals.

*Training*: This phase focuses on individual or group sessions where patients train their paretic limbs and cognitive functions under therapists’ guidance. Activities such as preparing a meal or dressing are deconstructed into small tasks, allowing patients to reconstruct a lost ability. During this phase, the environment, tasks, activities, and therapist’s attitudes are adjusted based on the decisions of the neurorehabilitation team. Assessment tools, and training tools—such as hand exercise balls, mirror therapy boxes [67], and rehabilitation technology [68]—are used to track and improve therapy outcomes. Moreover, therapists emotionally support patients to ensure they maintain high motivation.

*Reflecting*: This phase involves team members sitting with patients to reflect on their performances and set new goals (re-*Planning*). This allows patients to become aware of their motor-cognitive capabilities and accept their limitations.

*Discharging:* This final phase marks the end of inpatient care and usually the beginning of outpatient rehabilitation, with a complete treatment process following. Outpatient care focuses on continued recovery and aims to maximize independence over time [69]. This therapy does not require a hospital stay but is often provided through a clinic, day hospital, or at a patient’s home [5]. The same team of experts who planned the inpatient treatment determines if an individual can begin with outpatient rehabilitation: They must establish if a patient has improved their physical ability and can transfer the skills learned during inpatient care to their home environment.

Overall, our observations allowed us to better empathize with patients and neurorehabilitation experts and understand how treatment is tailored to each individual patient’s specific needs, goals, and capabilities.

### Study 2: semi-structured interviews

The thematic analysis [56] focused on the research question: *What strategies do therapists adopt to create suitable training environments for patients?* After transcribing and familiarizing ourselves with the semi-structured interviews, we generated 151 codes representing the meanings and patterns discovered in the data. We then organized these codes into six groups based on shared concepts. Each group describes the quality of the training environments: *Specific*, *Meaningful*, *Versatile*, *Educational*, *Safe*, and *Supportive*. In the following paragraphs, we describe the six groups with examples of supporting participant statements. We value using inclusive language. Yet, the use of the pronouns “he” and “she” was not modified from the participants’ statements, as we also value reporting verbatim transcripts.

#### Specific

The theme *Specific* refers to the patient’s characteristics (e.g., clinical needs, personal goals, social circumstances, personality) that therapists consider when preparing the training environment. Therapists need to know as much as possible about their patients to create training conditions that help them function properly in their environment. This requires seeing patients as a whole to get a clear understanding of how their acquired brain injury influences (physically, behaviorally, cognitively, and emotionally) their functioning and behavior in daily life, and organizing the training environment so that it can meet the set requirements:

- “*There are a lot of things I want to know about the patient. I want to know what someone did before the stroke.. what was his way of life before?.. what kind of job did you do? How does her social life was?.. I have to make clear how someone is doing right now like: can you be independent in movements?.. What’s the level of performance right now?*”* (P05)*
- “*.. every patient is unique.. everyone has different problems, different goals; every person has different needs or different questions.. every problem, every disability is different; there are a variety of ways to do exercises.*”* (P09)*

Additionally, the qualities of a specific training environment can vary even between patients who want to practice similar daily activities, e.g., depending on their social background and social support:

- “*.. patients that come in here that have less socio-economic status or are not as intelligent, they complete less complex tasks in the daily living.. [instead] if you’re high-functioning executive, you [might] need to be able to organize a team.. if you.. only need to hand out newspapers in the morning, it uses a whole other base of functions..*”* (P01)*
- “*.. if you have family support or not.. if you have children and at what age.. they can affect you in your daily life.. maybe one patient.. has more risk factors to develop some kinds of cognitive or physical situations.. because they are more likely to develop a fear of [to perform] the movement.. or.. depression..*”* (P08)*

#### Meaningful

*Meaningful* refers to real-life conditions. Training environments should not be decontextualized, as this could demotivate patients; instead, they should feel naturalistic and realistic to patients. Training environments should be functional and meaningful, imposing challenges coherent with what patients may encounter once discharged.

- “*.. [if] I'm not living here in the city center, so I'm just from a small village, so my life is in a small village, meaning that it is less crowded, so why do I need all the information about this city?..*”* (P03)*
- “*.. if.. his daily job was cleaning, and that's his main job; then, I know that if we're going to do something like finance, he may not do that very well because he didn't have to do it before..*”* (P05)*
- “*.. the functionality of the activity depends on the relevance of the activity for you.. maybe the activity is the same.. [e.g.] I want to [learn how to] reach my mobile phone because I’m an influencer, but you are a writer–so, you need to reach the pen.. the movement in your body is the same.. but the environment.. is different!*”* (P08)*

Yet, some patients may struggle with transferring the skills learned in training environments to their own home environments. These patients may not be able to imagine how to execute a task in an environment different from the training one:

- “*.. some patients can only make coffee at home. If they are over here [rehabilitation center] or maybe in another kitchen, they can’t find the thing.. they can’t imagine the solutions to different environments..*”* (P05)*
- “*.. we are not always having the possibility to go home and practice.. you want to give them more views of what can happen and how they can react.. (but) they can’t imagine..*”* (P06)*

#### Versatile

*Versatile* relates to the different stimuli that can be manipulated in the training environment to facilitate functional task execution (e.g., secondary tasks, sounds, group or individual sessions, and the distance of people and objects from the patient). The manipulation of the characteristics of a training environment may influence patients’ attention and the risks of making mistakes:

- “*.. I let her cook in her own kitchen.. and I let someone call.. that [became] a very mistake.. she was just talking about everything, and it became black in the kitchen–she forgot about there was something on the fire..*”* (P06)*

Yet, environments should offer patients the possibility to train under different conditions that can be manipulated, such as practicing alone instead of in a group, filtering out distractions, including secondary tasks, and auditory and/or visual distractions:

- “*..[here] it’s silence, and there is no distraction, so, I can focus and I can read your paper.. when.. there are other people walking outside there, I get a phone call, or there’s someone who’s knocking on the door, and my attention is like everywhere, so there is so much more interaction in daily living than here in this room..*”* (P03)*
- “*.. when you wear sunglasses, they get less overstimulation and less distraction because it filters away impulses [stimuli] from outside.. some patients also wear headphones when they want to lower the noises around them, when they go to the store and so they can better cook and do their stuff. Then, when in a crowded room, they shut down..*”* (P11)*

While too many stimuli can become overwhelming, sometimes additional stimuli can further engage the patient and facilitate their adaptation to the training environment:

- “*.. music can help with adapting..*”* (P11)*

#### Educational

*Educational* relates to patients being educated and made aware of their capabilities. In training environments, patients should be allowed to explore the environment and make mistakes to learn from them. Thus, all elements in a training environment should be accessible for interaction. Training environments should promote understanding to help patients gain insight into their functioning.

- “*Just let them make a big mistake and try to let them see what happens and why this can be dangerous in the future in their own home when there’s no one around!*”* (P06)*
- “*.. if you don’t know about something, you are going to imagine the worst situation.. when they have information about what happens to them, why they have these symptoms now, and what’s the natural prognostic to these kinds of symptoms—they understand it and then their mindset changes a lot..*”* (P08)*
- “*.. if you write down in words what you are saying.. they can hear and read the information that they need to understand.. if they want to tell something.. they can point at it..*”* (P09)*

Importantly, training environments should encourage patients to self-reflect on their actions and express themselves, thus promoting awareness and acceptance of their new condition:

- “*.. to help him in the rehabilitation and learning process.. we try to help the patient to get insight into its functioning to recover better..*”* (P04)*
- “*.. there are also a lot of people who.. think they can do everything, and they don’t need help.. you have to let them see the change they have [endured]..*”* (P06)*
- “*.. you have to teach them that it’s ok to have another life.. they have to learn that the life that they have now is also ok..*”* (P08)*

Yet, to promote learning, training environments should also promote repeating the same task and following a given order.

- “*.. you have to make sure that all the steps are done in the same order.. and make sure they are doing it in automatic pilot and for as long as possible.. If they don’t know.. I do all the steps myself.. and I show them, and then you can slowly let them do it..themselves*.”* (P10)*
- “*.. you have to make sure that all the steps are done in the same order.. and make sure they are doing it in automatic pilot and for as long as possible.. If they don’t know.. I do all the steps myself.. and I show them, and then you can slowly let them do it..themselves*.”* (P10)*

#### Safe

*Safe* refers to training in an environment where patients can make mistakes without the risk of injury or experiencing unnecessary stress:

- “*.. we have to make sure that someone can try and provide a safe space to learn.*”* (P04)*
- “*.. [if] I'm going to do a task in the kitchen that isn't that complicated.. and if I see that it's very complicated for the person, I know that I have to make it easier..*”* (P05)*

To protect patients from physical harm and psychological discomfort, training environments should help balance activity levels to ensure rest periods, thus preventing overexertion or fatigue:

- “*.. if we notice that someone gets tired.. they are closing their eyes.. blink a lot.. start to shut themselves off.. we make sure that these tests are done at a different time..*”* (P01)*
- “*.. you have to make sure that he takes a rest at the right time, or [if] he has some problems with focusing.. he has to be somewhere in a quiet space..*”* (P05)*

*Safe* also refers to a training environment characterized by trust and empathy, facilitating clear and effective communication between therapists and patients who feel comfortable openly sharing their feelings:

- “*.. let people trust you to share the information and focus on how it was and where you are now.. we have to give them perspective, and you won’t give them.. false hope..*”* (P03)*
- “*.. it is not only doing exercise but also just to talk about how they feel, how they are doing and if they are ok.. you create a bond—a connection with the patient..*”* (P09)*

#### Supportive

*Supportive* relates to motivation and the physical and verbal support therapists give to patients.

- “*.. we’re looking for the motivation which is in the patient to get it out and work with.. from the motivation, go further and further to stimulate the patient even when he’s sad or anxious.*”* (P04)*
- “*.. the key.. is to keep them motivated.. you have to know very good your patient, to know what motivates them.. because sometimes they want to give up..*”* (P08)*

To promote motivation, training environments should act as intersections where patients are engaged in meaningful social interactions that encourage collaboration, peer support, and training of the desired skills:

- “*.. if they want to go home and there are some skills they have to learn to go home, then they are extra motivated..*”* (P05)*
- “*.. if the patients really would like to go to the markets, you want to give him a possibility to exercise in conversation on the markets..*”* (P09)*

Additionally, how support is provided to patients is important. This includes the provision of positive feedback, the ways of reporting a message (e.g., directly or indirectly), the use of alternative means to provide instructions (e.g., verbal, written instructions, or physical interactions to assist movements), and the distance of people or things from the patient (e.g., sitting next to patients, standing far from them).

- “*.. you have to find out what their expectations are and how they interact with people, I connect to that and that is also changing my language.. if you have a higher education, these people usually are more problem-solving kind of types: they need information, they understand.. other people get confused by the information.. the communication aspects in the environment do predict or do provoke behavioral outcomes..*”* (P02)*
- “*.. when they are having aphasia, you make sure you talk slower, make shorter sentences, and ask closed questions where they can answer yes or no..*”* (P10)*
- “*.. the positive feedback of the team, it helps them a lot.. to function, to regain.. recovery.. and working with the plan and with the timetables.*.”* (P11)*
- “*.. try to stand next to the other, and just do it together and find out together what's the best way to go..*”* (P06)*

We used the quotations in each group to formulate *twenty-two statements* that would answer our research question. These statements*,* which report the strategies therapists seem to adopt in conventional neurorehabilitation to create suitable training environments, were subsequently incorporated into an online questionnaire in Study 3.

### Study 3: online questionnaire

Table 1 summarizes the mean ratings for the 22 questionnaire items and their corresponding themes. For each statement, participants could comment on how they use it in their daily activities; below are some transcripts of participants’ comments left online. Overall, the neurorehabilitation experts strongly agreed that the training environment should be adjusted to the cognitive abilities and social environment of the patient (Items 1 & 4 in Table 1).

- “*.. we have recently started working with an interdisciplinary cognitive treatment plan in which all therapists have the same approach in these areas and take this into account in every therapy*”* (Item 1—P11)*

**Table 1:** Therapists’ agreement with statements from 1 (strongly agree) to 5 (strongly disagree)

Item No.	Theme	Statement	*M* (*SD*)
4	Supportive	During therapy, therapists should adjust the levels of interaction* with their patients, depending on their cognitive capacities, to improve motor learning	1.13 (0.34)
1	Specific	In the preparation of the training environment, therapists should **take into account the physiological and psychological capacities** of the individual patient, **with understanding for their social circumstances**	1.17 (0.48)
5	Educational	During therapy, therapists should **choose between learning through mistakes and errorless learning strategies** to improve motor learning, depending on the cognitive capacities of the patient	1.38 (0.82)
14	Meaningful	During therapy, therapists should **adapt the realism of a task** to the cognitive capacities of the patient to improve motor learning	1.42 (0.65)
2	Versatile	During therapy, therapists must **adjust the environment** to the progress and recovery goals of the patient	1.46 (0.59)
11	Versatile	During therapy, therapists should, depending on patients’ cognitive capacities, choose whether to **place patients in large and crowded rooms** (group sessions) **or small and isolated rooms** (1-on-1 sessions) to improve motor learning	1.46 (0.59)
18	Supportive	During therapy, therapists should **choose from verbal, gesture, or written instructions**, adapted to the cognitive capacities of the patient to improve motor learning	1.50 (0.78)
16	Supportive	During therapy, therapists should **give patients motivating feedback**, depending on their cognitive capacities, to improve motor learning	1.50 (1.02)
12	Versatile	During therapy, therapists should **adjust the exposure to background noise or unintended sounds** according to the cognitive capacities of the patient, to improve motor learning	1.67 (0.76)
20	Versatile	During therapy, therapists should choose to **give patients secondary tasks**, depending on their cognitive capacities, to improve motor learning	1.75 (0.79)
8	Meaningful	During therapy, therapists should **introduce familiar elements or mimic familiar conditions** to train patients’ motor functions, depending on their cognitive capacities	1.79 (0.59)
3	Safe	During therapy, **therapists and patients should decide together** whether they want to change the training environment, depending on their cognitive capacities to improve motor learning	1.88 (0.90)
19	Supportive	During therapy, therapists should **involve the patient’s family members**, depending on the cognitive capacities of the patient, to improve motor learning	1.88 (1.03)
10	Meaningful	During therapy, therapists should provide patients with work-specific tools*—depending on their cognitive capacities to improve motor learning	1.96 (0.81)
13	Versatile	During therapy, therapists should **modulate the direction of light (spotlight or diffuse light) and intensity,** depending on the cognitive capacities of the patient, to improve motor learning	2.13 (0.85)
9	Meaningful	During therapy, therapists should choose to **expose patients to conditions typical of a city or village**—depending on where they live—to improve motor learning, depending on their cognitive capacities	2.17 (0.82)
17	Safe	During therapy, therapists should **influence patients’ stress levels**, depending on their cognitive abilities, to improve motor learning	2.17 (0.92)
21	Supportive	During therapy, therapists should **let patients communicate with other people**, depending on their cognitive capacities to improve motor learning	2.17 (1.01)
22	Educational	During therapy, therapists should choose to **restrict the movements of the patient's less affected arm** to train the more affected arm, depending on their cognitive capacities to improve motor learning,	2.33 (0.87)
6	Educational	During therapy, therapists should **adopt the use of mirrors**, allowing patients to watch their movements to improve motor learning, depending on the cognitive capacities of the patient	2.33 (1.05)
15	Specific	During therapy, therapists should **choose between unilateral or bimanual exercises**, depending on the cognitive capacities of the patient to improve motor learning	2.50 (1.14)
7	Educational	During therapy, therapists should **use video recordings of previous training sessions** with patients, depending on their cognitive capacities, to improve motor learning	2.67 (0.96)

The authors added the boldface text for clarity, but it was not part of the original questionnaire. Also, for brevity, we removed some text (e.g., explanations of the level of interaction) in Items 2, 4, 6, 8, 10, 12, 13, 14, 17, and 20

Additionally, choosing between error-free learning or learning from mistakes (Item 5) and modulating the realism or complexity/distractions of the task (e.g., Items 2, 12 & 14) were generally agreed-upon approaches. However, in some cases, the manipulation of the level of complexity of the environment should be enforced due to patients’ disorders, as commented on by some respondents:

- “*.. it is certainly important that you make the conditions as pleasant as possible for the patient.. lights off, blinds closed, removing ambient noise.. as soon as the patient can tolerate this better, slowly increase/expand the stimuli*”* (Item 2—P02);*
- “*If the patient experiences problems with communication in background noise, exercises are offered in which the patient learns to deal with these stimuli, i.e., participation in a group or radio on during an exercise, ask colleague to disturb during a treatment*”* (Item 12—P11)*

Motivational techniques (Item 16), co-deciding with the patient, and involving the family (Items 3 & 19) were also regarded as relevant:

- “*Shared decision-making with patients: very important to find out what their end goals look like and how you can imitate them—or build them up in steps*”* (Item 3—P24)*
- “*It is important to involve family members in the rehabilitation so that the patient can put what they have learned into practice at home*”* (Item 19—P09)*

Relatively low ratings, but still on the ‘agree’ side, were provided for more specific educational techniques, including the use of mirrors (Item 6), video (Item 7), and the constraining of movements (Items 15 & 22). According to participants, their use is not ordinary but depends on the therapy goals, patients’ motor abilities, and behavior.

- “*Depending on whether this is helpful…Can be like that in certain situations, this doesn't seem like a 'must' to me.*”* (Item 7—P17)*
- “*If the patient is inclined to let less affected arm things take over, immobilize them by, e.g., slinging*”* (Item 22—P05)*

### Study 4: participatory design workshop

While the previous sections focused on conventional non-VR-based sensorimotor neurorehabilitation, the present section reports findings from the focus group and the ideation session that describe neurorehabilitation experts' opinions and ideas on IVR environments.

#### Study 4—activity 1: focus group

The paper sheets reporting participants’ ideas can be found in Additional file 3.

#### Q1. What do you think about and what are the advantages and disadvantages of IVR?

Participants indicated that IVR would require fewer therapists to train the same number of patients. They also noted the versatility of IVR, including having many exercises built-in to train for different impairments, use at home, and creating targeted challenges adjusted to meet the patient’s specific clinical needs and responses.

- “*.. you can also cover different disciplines, different areas. You only need one tool..*” (Group B)
- “*.. you do traffic training with a patient.. you want to see if they pay attention to the left side.. you think: maybe I should have had a few cars and cyclists coming from the left side, you can simply build them in the VR.. targeted challenges for practice..*” (Group A)
- “*.. you don’t have to use it here, but maybe at home*” (Group B)

When discussing the potential disadvantages of IVR, participants remarked that this is a costly technology and not everyone might benefit from it. Some patients might be unable to handle it cognitively or understand how it works. They also expressed their concerns about safety and excessive use. Some patients might feel unwell while using IVR (i.e., cybersickness), while others, due to excessive use, might have difficulties with transferring the skills learned in the virtual world to the real one.

- “*.. not everyone can use it.. it’s only suitable for a select group*” (Group B)

#### Q2. How would you use IVR in rehabilitation?

Participants recommended that therapy in IVR should be targeted to the patient’s clinical impairments. They also discussed the importance of providing training within diverse real-world inspired scenarios, particularly those outside rehabilitation centers.

- “*.. simulate activities that you can’t do here..*” (Group A)
- “*.. traffic training seems like a good idea.. groceries*.” (Group B)

Their suggestion of taking pictures of patients’ homes further underlined the idea of creating virtual experiences that closely mimic real-world environments. Additionally, participants expressed interest in using IVR to simulate home visits, allowing therapists to virtually enter patients’ homes to provide remote assistance and guidance.

- “*.. the ideal thing would be to put on the device, walk into someone’s home, and then help them*” (Group B)

Finally, they valued IVR for its potential as a tool for research, exploring related possibilities, such as eye tracking—even though eye-tracking is not directly linked to IVR and is only available in some HMDs—, and to facilitate learning by exploring, e.g., visual feedback.

- “*.. what does someone do with their peripheral vision, you can measure all of that in VR with eye tracking.. you can potentially use that in your visual research..*” (Group A)
- “.. *you could apply it to different cognitive domains.. if you do something wrong, a signal comes up.. if you put your hands in the wrong position, you get a.. buzzer alarm.. or you get a red cross or.. just won’t move forward.*” (Group A)

#### Q3. What should researchers consider when developing IVR experiences for brain-injured patients?

Overall, participants recommended that developers create engaging experiences that can be adjusted to the patient’s physical and cognitive capabilities, and age.

- “*Fun interactive exercises.. enjoyable and challenging.. exercises that change every time*” (Group A)
- “*.. one person might have a lot of cognitive problems in the beginning, but you can also create a certain progression or level of difficulty.*” (Group A)
- “*.. you should be able to adjust it so that you can put it on and operate it with one hand.*” (Group B)
- “*Suitable for each person.. so that you don’t only have super modern things that young people have an affinity with, but also for seventy-year-olds.*” (Group A)

Concerns were also raised around safety, highlighting the need to develop experiences that limit the risks of getting physically injured or fatigued during use.

- “*.. somehow measure whether it becomes too much for the patient.. monitoring fatigue or something, or attention going down.*” (Group A)
- “*.. if you have real physical problems, and you’re in such a world and you think: oh! I’ll stand up! There should be.. limitations because it’s about what someone can’t do..*” (Group B)

Further discussion was on the modalities of how to guide or instruct patients during use:

- “*Demonstrations, show example video, instructional video, little text, simple language, short sentences.*” (Group A)
- “*Demonstrations, show example video, instructional video, little text, simple language, short sentences.*” (Group A)

#### Study 4—activity 2: ideation session

The paper sheets reporting participants’ ideas can be found in Additional file 3. Groups A and B produced similar results regardless of the persona assigned, probably because they were tasked with designing both low and high-cognitively demanding virtual environments.

#### Low-cognitively demanding virtual environments

**Fig. 5:**
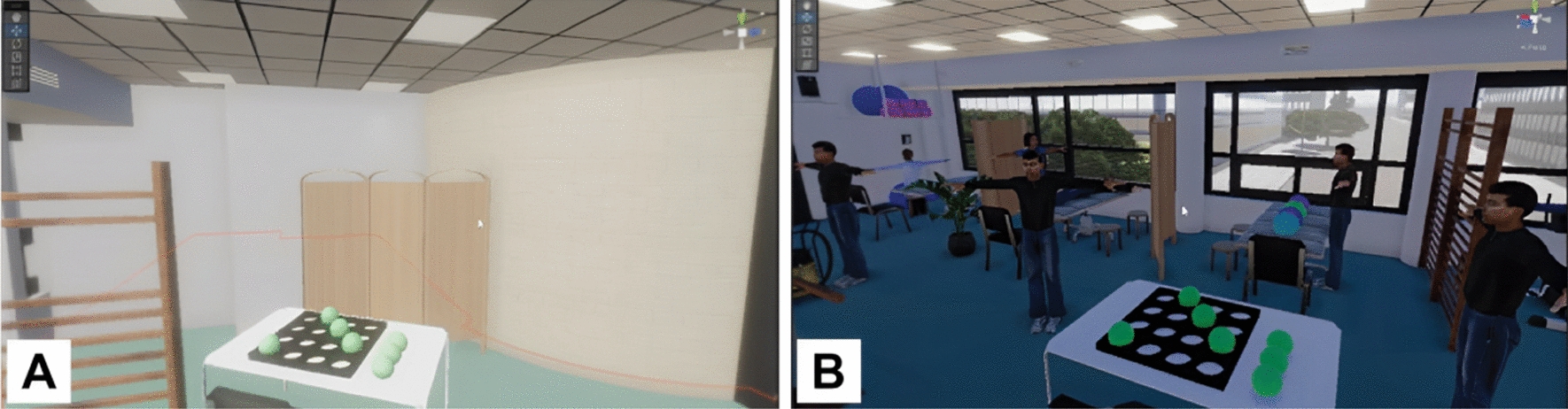
Screenshot from a video recording of one of the monitors used to display to the participants what the user saw in the HMD. These are examples of: **A.** a low-cognitively demanding environment with no distractions; and **B.** a high-cognitively demanding training environment , co-created by Group B in collaboration with the VR developer

In low-cognitively demanding virtual environments, the priority is ensuring the user performs the given task. For patients with reduced cognitive abilities, it would be beneficial to train in static and empty environments (Group A) and simplified environments with none or minimal distractions (Group B), as depicted in the generated IVR environment by Group B—Fig. 5A.

- “*.. the first stage could be where there’s nothing happening around you as you move..*” (Group A)
- “*.. you want to get all the distractions away. You don’t want to place him in front of a window because he can see all the movements outside. You don’t want a lot of people to walk by, that’s why you want to face him to the wall or the screen.*” (Group B)

**Fig. 6:**
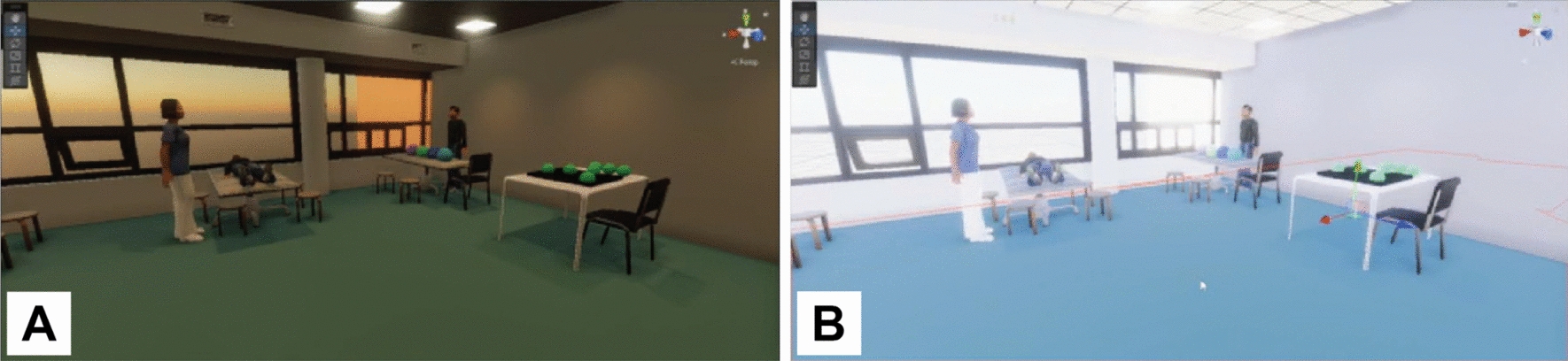
Screenshots from a video recording of one of the monitors used to display to the participants what the user saw in the HMD. These are two examples of the same virtual environment where light intensity and temperature were manipulated to: **A.** decrease or **B.** increase the intensity of sensory input, as suggested by Group A to the VR developer

Both groups also raised concerns about the effects of bright colors and excessive noise on patients, as too much noise or light can affect attention (see an example of different lighting generated by the participants in Fig. 6). For instance, while Group A suggested using calming colors and sounds from nature and classical music, Group B recommended neutral colors and music familiar to the patient.

- “*Loud music sounds, voices, changes in sounds.. the low demanding side is nature sounds and classical music.*” (Group A)
- “*.. sometimes we try to add music or things that are important to people so they are more focused on the task.. music that they prefer, to make them more alert.. the music to help patients concentrate.*” (Group B)

**Fig. 7:**
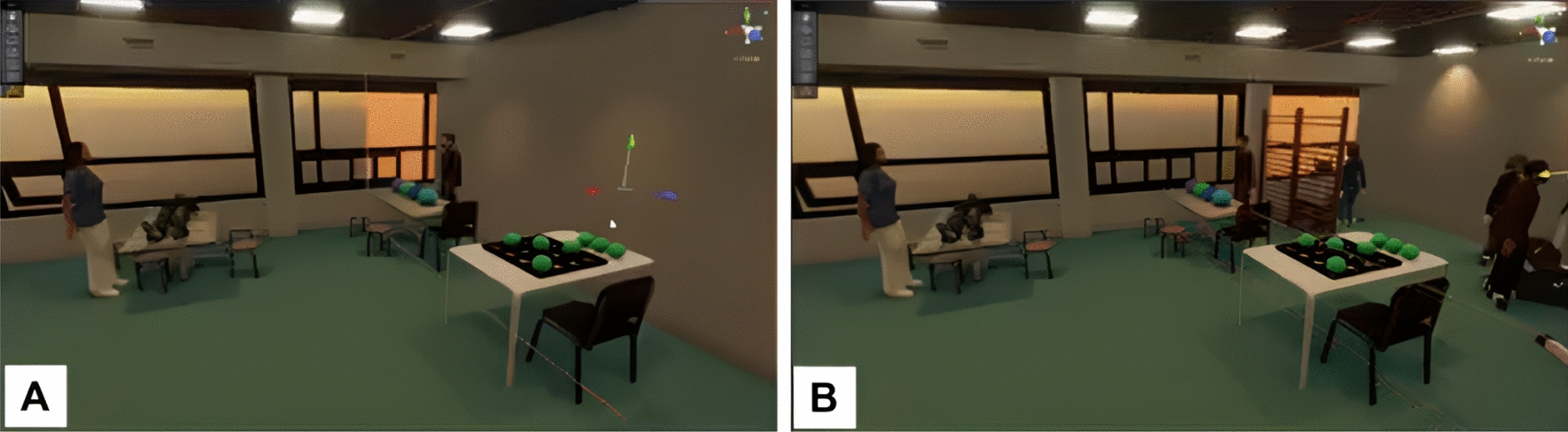
Screenshots from a video recording of one of the monitors used to display to the participants what the user saw in the HMD. The figures illustrate the concept of a movable virtual wall within the virtual environment, designed to manipulate the amount and direction of stimuli. Participants suggested that this strategy could be beneficial for patients with neglect, as it would facilitate training of the affected side by forcing attention to the only presented objects. This concept was co-created by Group A in collaboration with the VR developer

This discussion also led to the benefits that some patients could experience with increasing and decreasing the number of stimuli. In particular, Group A suggested that for some patients, such as those suffering from spatial neglect, showing only one side of the virtual environment could help train the affected side, as the other half of the environment would be hidden. This is presented in the generated IVR environment shown in Fig. 7.

- “*.. a wall can be very useful because very often people with hemianopsia or neglect drive towards something and then there’s a wall. They can’t get past it. And if you can create more stimuli there, or trigger them that when there’s a wall and I notice I can't go further, they are challenged to go the other way.*” (Group A)
- “*.. you want all the stimuli to come from one side so that people have to pay attention to that side, so they have to look in a certain direction*”* (Group A)*

Finally, low-cognitively demanding virtual environments should provide an introduction to the training using a video showing how to execute a task. Video may enhance comprehension of instructions, especially among patients with speech problems (i.e., aphasia).

- “*.. the instruction in the example video.. a video with someone demonstrating it, so you don’t have to rely on language..*” (Group A)

#### High-cognitively demanding virtual environments

For high-cognitively demanding virtual environments, both groups described them as being dynamic, presenting increased sensory input and distractions, such as bright lights and loud sounds, moving objects (e.g., windows, doors, blinds, people), and challenging patients with secondary tasks. Figures 5B and 6B show examples of high-cognitively demanding virtual environments created by the participants.

- “*.. stimuli are all around you.. from multiple sides.. from left and right, increasing the one, reducing the other.. something can come from behind on the left, while you have to focus on the right, but there's a lot happening behind you.. imagine being in a space where people are actively exercising or running, and you're right in the middle of it..*” (Group A)
- “*.. let people walk by.. in and out of the room.. people start conversations with him to see how he manages the task he’s working on.. what I would do is a person in the kitchen.. I would add a lot of music, add a lot of bells, people getting in and out and grab something out of the kitchen*” (Group B)

Both groups emphasized the importance of indoor and outdoor environments for training. They also suggested environments resembling real-life ones (e.g., kitchens, bathrooms, public spaces), as they could facilitate the training of daily life activities.

- “*.. someone has a young baby at home, you can add, for example, baby noises..*” (Group B)
- “*With a lot of distractions, and you prefer to keep it as close to what they are used to. So their kitchen, their supermarket, their devices, the things they are using.*” (Group B);
- “*.. public transportation.. public locations.. bathroom, kitchen.. supermarket, traffic.. you’re in a car..*” (Group A)
- “*.. a concert because a lot of people think.. they can handle it.. or a party from family, social environment, birthday!*” (Group B)

Group A further discussed the topic of instruction and suggested adding simultaneously verbal instructions on top of other modalities to challenge the patient further.

- “*.. like a chalkboard where you put the instructions step by step.. you can also verbally explain it.. with verbalization because then you got double input because you got more information you can read here.*” (Group A)

In summary, both groups discussed the same types of stimuli—visual, auditory, and cognitive—and how their manipulation would ensure the creation of different IVR experiences. Only Group A discussed the need to provide instructions by using different means, from video to verbal and written instructions. Besides, groups shared similar ideas about low-cognitively demanding environments being static, empty, and silent, and high-cognitively demanding environments resembling real-life environments (e.g., kitchens, supermarkets, metro and train stations, shopping streets), moving elements (e.g., objects, people), bright lights, and loud sounds.

## Discussion

This work presented a human-centered design approach to identify recommended characteristics of immersive virtual training environments for ABI patients. With our research, we specifically aimed to learn about training environments and how they could be manipulated to enhance recovery. Based on knowledge gained from contextual research in a Dutch rehabilitation center and semi-structured interviews with eleven neurorehabilitation experts working within it, we defined the *five steps* of the inpatient rehabilitation experience (Study 1) and *six themes* describing the characteristics of suitable training environments (Study 2). Our findings were verified (Study 3) by 24 neurorehabilitation experts working in Dutch institutes who answered a questionnaire online to express their degree of agreement with twenty-two statements derived from Studies 1 and 2 representing strategies that physical and occupational therapists use to create suitable training environments for ABI patients with different cognitive capabilities. In Study 4, eight neurorehabilitation experts co-created with VR developers examples of low- and high-cognitively demanding IVR training environments for brain-injured patients. Thus, we gained insights from conventional rehabilitation (Studies 1–3) and generated recommendations based on all four studies, with Study 4 specifically informing IVR design.

### Insights gained from conventional training environments in neurorehabilitation

This section discusses findings from Studies 1–3, which focused on conventional non-VR-based sensorimotor neurorehabilitation and training environments.

The picture that emerged from Study 1 was a recovery process consisting of five phases: (1) *Screening*; (2) *Planning*; (3) *Training*; (4) *Reflecting*; and (5) *Discharging*. Patients iteratively go through the first four phases while a multidisciplinary team monitors their progress and performance, and ensures tailored treatments. These findings are in line with previous studies that report that stroke rehabilitation typically entails a cyclical process involving: (1) *Assessment* or *Admission* to identify and quantify the patient’s needs; (2) *Goal setting* to define realistic and attainable goals for improvement; (3) *Intervention* or *Participate in therapy* to assist in the achievement of goals; (4) *Reassessment* to assess progress against agreed goals; and (5) the *Discharge* phase indicating the end of the inpatient and the beginning of the outpatient experience [5, 62, 70].

Study 2 contributed to understanding how therapist-patient interactions and conventional training environments are tailored to suit patients with different capabilities. We identified six themes describing the characteristics of suitable conventional training environments: (1) *Specific;* (2) *Meaningful*; (3) *Versatile*; (4) *Educational*; (5) *Safe*; and (6) *Supportive*. Our observations were further verified by the online questionnaire in Study 3.

Our first three studies highlight the importance of considering patients holistically (*Specific*), not only from a clinical point of view but also by understanding their preferences and needs, as indicated by the model of *Person-centered care* [71]. Achieving such an understanding requires the collaboration of experts from different fields of specialization who know how to support patients and satisfy their diverse clinical needs. Family members are also involved [72], as considering patients’ social context and support networks helps therapists identify sources of motivation to support them across the recovery process [73].

According to the neurorehabilitation experts, training in environments that are *Meaningful* to patients can increase patients’ motivation and promote the transfer of skills learned during therapy to their daily lives. We also found that tailored (*Versatile*) physical environments may influence the patient’s attention during task execution and support therapists in maximizing patients’ recovery. Therapists tailor the training environments to meet patient needs. This finding relates to the research on environmental enrichment, which supports the idea that exposure to environments that foster voluntary engagement in physical, cognitive, and social activities may improve patients’ rehabilitation outcomes [19]. The neurorehabilitation experts listed several elements that could be manipulated to meet patients’ cognitive capabilities, e.g., the size of the training room, the level of surrounding noises and light, the interactions between patients and personnel, and the provision of feedback, among others. Previous investigations on the role of the physical environment of hospital buildings showed that physical environments could affect clinical outcomes, patient experiences, safety, efficiency, and cost [74, 75] and that rehabilitation spaces should allow for privacy without isolation and provide a patient-centered environment that is coherent and convenient for stroke survivors [76]. Finally, our results stress the importance of assisting patients in understanding their new functioning (*Educational*) through effective communication and trusting relationships between therapists and patients to provide a *Safe* and motivating (*Supportive*) training environment. This is supported by motor learning literature, which indicates that providing feedback and performance information tailored to the learner's current abilities can enhance skill acquisition and task performance (see Challenge Point Framework [37]). Building therapeutic relationships [93] and encouraging people's intrinsic motivation are also crucial (see OPTIMAL theory [77]). Motivational strategies such as establishing a therapeutic alliance, setting attainable goals, and offering emotional support [78] can help stroke survivors meet their rehabilitation goals and improve outcomes.

In short, results from Studies 1–3 can be summarized in the list of recommendations to create suitable conventional training environments listed in Table 2.

**Table 2:** Recommendations for creating suitable conventional training environments, derived from Studies 1–3

Recommendations	Explanation
1. Understand the patient’s physical, behavioral, cognitive, and emotional functioning (Specific)	Understand how the ABI impacts patients’ physical, behavioral, cognitive, and emotional functioning, as well as their social background and available social support, to create an environment that meets their needs
2. Provide patients with coherent challenges (Meaningful)	Let patients train in environments that replicate real-life conditions and challenges coherent with the exercises they will perform in daily life
3. Manipulate patients’ exposure to stimuli (Versatile)	Control different types and amounts of visual, auditory, and cognitive demands to meet patients’ cognitive capabilities
4. Facilitate that patients gain insights into their functioning (Educational)	Assist patients in understanding their new functioning, while training in environments that encourage making errors
5. Ensure that patients can learn without risks (Safe)	Promote a balance of physical activities and resting periods, and honest communication between therapists and patients
6. Engage patients in motivating environments (Supportive)	Encourage patients to reach their full potential by engaging in social interactions that sustain both their progress and setbacks. Provide them with feedback and use varied communication methods

### Recommendations for designing virtual training environments

This section discusses findings from Study 4, which focused on immersive virtual training environments.

In Study 4, neurorehabilitation experts provided examples of low- and high-cognitively demanding immersive virtual training environments. Overall, they suggested the design of virtual environments targeting patient-specific neurological disorders, such as visual neglect [79]. Moreover, they indicated that virtual environments should offer the appropriate amount and level of stimuli based on the patient’s capabilities and current state. This is supported by literature arguing that the provision of stimuli, such as auditory or visual, affects people’s workload [80]. These findings—which are consistent with the results from Studies 1–3, specifically, with the recommendations “*Understand the patient's physical, behavioral, cognitive, and emotional functioning (Specific)*” and “*Manipulate patients’ exposure to stimuli (Versatile)*”—indicate the need for personalized virtual training environments.

Additionally, neurorehabilitation experts proposed virtual environments that allow patients to practice in *realistic* and *meaningful* settings that focus on replicating their real-life contexts and training their desired daily activities. Similar findings were reported in previous research, which suggests that realistic interactions and environments are important for patient motivation and effective rehabilitation. This includes a study in which health professionals and technology specialists co-designed and evaluated a VR application prototype for communication rehabilitation [50], as well as a focus group study with rehabilitation specialists exploring the use of shopping malls during rehabilitation [81]. Although the neurorehabilitation experts involved in our Study 4 argued that virtual environments should be realistic and meaningful to patients, this still needs to be empirically substantiated. Observations from simulators in other domains, such as flight simulators or medical training simulators [82, 83], show that there is a tendency or desire to create increasingly realistic simulators, which carries the risk of increasing cost without necessarily having a proven effect on the transfer of learning. In the field of motor learning, a common assumption is that to improve skills in a given task, the practice should be conducted under conditions, including environment and movements, that closely resemble those of the actual task setting, in accordance with the *specificity of learning hypothesis* [84]. Nevertheless, therapists pointed out that the level of realism should be adjusted to meet the cognitive capacities of the patient and that patients should be provided with more scenarios of what could happen and how they could react under different conditions. This is in line with literature that suggests that variability of practice is essential to generalize the acquired skills to different contexts [84]. These results are in line with those from Studies 1–3; specifically, with the recommendation “*Providing patients with coherent challenges (Meaningful)*”. However, some neurorehabilitation experts were also concerned about spending too much time in virtual training environments as it may make the transfer of learned skills to daily life more difficult for some patients.

Participants also discussed safety concerns, focusing mainly on physical safety and ensuring patients rest during IVR training. They noted that patients may get physically injured and prolonged training sessions may lead to overstimulation, fatigue, and physical risks. Similar concerns were discussed in a study on XR-based exergames for motor rehabilitation involving, among others, therapists and ABI patients, where patients’ disabilities and lack of ability to judge their capabilities were identified as factors increasing the risks of injury [47]. In our study, participants suggested monitoring attention and fatigue—potentially through eye movements and behavioral measures (e.g., reaction time)—to enforce resting periods. Incorporating objective measures of people’s responses into a VR system may help ensure that the amount of practice is appropriate for a given patient [85, 86]. This also aligns with [47] which recommended automatic adaptation of game mechanics based on a patient’s capabilities. Findings from Studies 1–3 address safety in similar terms.

Participants engaged in a discussion concerning the provision of instructions during training. They suggested different means to instruct patients, e.g., using videos for patients with disorders like aphasia, and written and audio instructions for less cognitively impaired patients. Finally, the use of feedback to inform patients about their performance, progress, and mistakes was discussed. This is aligned with previous research that had shown that the provision of congruent feedback about performance based on objective measures of movement may improve motor learning [87, 88]. The importance of providing feedback on performance has also been discussed in related domains, such as speech therapy using IVR [49].

In short, we extracted several recommendations from Study 4, also supported by Studies 1–3, for the development of suitable IVR training environments, which are summarized in Table 3.

**Table 3:** Recommendations for VR developers to create suitable virtual training environments, derived from Studies 1–4

Recommendations	
1. Control the complexity of virtual training environments to prevent overstimulation while maintaining patients’ motivation	
2. Create real-world-inspired environments that resemble patients’ everyday contexts	
3. Ensure patients’ physical safety by encouraging taking regular breaks, based on their physical and mental state	
4. Offer different instruction modalities, including videos, written, and/or audio instructions, based on patients’ specific cognitive capabilities	
5. Facilitate patients’ learning and self-reflection by providing feedback on their performance and progress	

### Limitations

Our work has several limitations. First, a limited number of neurorehabilitation experts working only in Dutch institutes participated in our studies, and most of them were from a single rehabilitation institute. Despite including neurorehabilitation experts from various disciplines and with different years of professional experience to ensure heterogeneity, the limited availability of participants required us to involve the same two physical therapists and two occupational therapists in our studies. The generalizability of our results in other countries may be limited due to national differences in the development of rehabilitation interventions for ABI patients, even within the European region [89]. Additionally, the ability to provide care may be influenced by variations in patient bedrooms and furniture between institutions [90]. However, our human-centered design approach allows for further evaluation to determine if experts in other countries share similar approaches to rehabilitation and opinions on IVR as their colleagues in the Netherlands.

Second, we did not involve patients and caregivers (e.g., family members) in our studies. Neurorehabilitation experts are the ones recommending therapies and interventions; therefore, their experiences and insights are important when developing new therapeutic solutions. However, missing the patients’ perspective in the workshop may have influenced the results, as experts might have focused more on optimizing training rather than on the subjective experiences of patients with different cognitive capabilities. Engaging patients with varying levels of ABI severity and different ages in future research activities is essential to capture their opinions on virtual training environments, examine the topic of motivation, and evaluate the usability of technology.

Third, although the neurorehabilitation experts argued that virtual training environments should be realistic and meaningful to patients (see Recommendation 2 in Table 3), this will still need to be empirically substantiated using a transfer-of-learning study with a control group (e.g., realistic vs. non-realistic IVR environment). Observations from other domains suggest that more realistic simulators may increase costs without necessarily having a proven effect on the transfer of learning [82, 83].

Fourth, language barriers may have limited the data collection process, especially in Studies 1 and 2. The principal researcher did not speak Dutch, and the participants’ preference for using their mother tongue led to some communication challenges. Language barriers could have also discouraged some people from participating. To address this issue, we created the online questionnaire in Dutch (Study 3), and let participants in Study 4 speak their language. We also involved a Dutch VR developer in the participatory design workshop and hired a Dutch student assistant to help with translations.

Another limitation is the use of the modal verb “should” in the twenty-two statements of Study 3. Although participation in the online questionnaire was anonymous, using this verb may have introduced response bias, as participants may have felt pressured to agree with what they perceived as the desirable answer or better conform to social norms.

Sixth, in Study 4, we let participants co-create examples of low- and high-cognitively demanding virtual environments starting from a virtual replica of a training room at Rijndam, an environment familiar to them. It remains an open question whether building their solutions from scratch might have resulted in more creative solutions. However, to ensure participants could understand the potential of IVR and generate original and out-of-the-box concepts, we showed six videos of different versions of the same virtual environment before the co-creation exercise.

A final limitation is that while the research team has been involved in multiple sessions of reviewing the results of the thematic analysis of transcripts and notes, the thematic analysis was conducted by the first author. Employing more researchers to analyze the data independently may have enhanced the reliability and validity of the findings.

### Implications for neurorehabilitation and future work

Our research aimed to address the question of how IVR training environments should be designed for ABI patients with different cognitive capabilities. Through our studies, we generated recommendations for VR developers and co-created examples of IVR training environments. In short, tailoring visual, auditory, and cognitive demands to patients' cognitive capabilities might provide patients with training experiences that are stimulating and not overwhelming. Additionally, creating realistic and meaningful virtual environments replicating real-life tasks and contexts could facilitate skill transfer from the virtual environment to real-world scenarios. However, these findings need to be validated and further developed in the final phase of the Double-Diamond model: Delivery [52].

Future research should engage patients and their caregivers in co-creation activities to capture their opinions on IVR training environments, further explore the topic of motivation, and evaluate the usability of technology. Research should also incorporate the opinions and experiences of chronic stroke patients and their therapists to provide a broader understanding of the potential of using IVR technology in settings like at home, where it can increase therapy dosage and accessibility, supporting continuous rehabilitation outside the clinical environment. Furthermore, replicating this work in other rehabilitation centers would allow exploration of whether therapists from other countries and/or cultures adopt similar strategies or have developed unique strategies to create suitable training environments. Finally, future research should establish the actual effectiveness of training in such personalized training environments, considering the extrinsic load due to the equipment.

## Conclusions

Our work identified the five phases of the recovery process (Screening, Planning, Training, Reflecting (re-planning), and Discharging) and six key themes describing the characteristics of suitable (physical) training environments: Specific, Meaningful, Versatile, Educational, Safe, and Supportive. According to neurorehabilitation experts, tailored physical environments can increase patient attention and support therapists in promoting recovery. In immersive virtual training environments, neurorehabilitation experts recommended adjusting stimuli levels to patient capabilities, offering realistic practice scenarios together with feedback, and monitoring attention and fatigue. Also, by considering patients’ preferences and needs holistically, therapists can enable training in meaningful and safe environments. According to neurorehabilitation experts, this approach may potentially help in maintaining motivation and promote skill transfer, although this would need experimental evaluation.

Future research should validate these findings in the Delivery phase of the Double-Diamond model, also engaging patients and caregivers. Additionally, since we focused only on sensorimotor neurorehabilitation, future research should investigate other areas of neurorehabilitation, such as speech and mental health therapy, to expand the potential benefits of this technology.

## Supplementary Information

Additional file 1.

Additional file 2.

Additional file 3.

## Data Availability

Data are available on Zenodo.org: 10.5281/zenodo.13136277. An earlier version of Studies 1–3 was presented as an extended abstract: Cucinella, S. L., De Winter, J., Grauwmeijer, E., and Marchal-Crespo, L. (2023). Towards personalized Immersive VR neurorehabilitation: a human-centered design. 15th International Conference on Virtual Rehabilitation, Montréal, Canada, July 23–25, 2023.

## Abbreviations

ABI: Acquired-brain injury
HMD: Head-mounted displays
IVR: Immersive virtual reality
