# Supplementary material for: Towards personalized immersive virtual reality neurorehabilitation: a human-centered design
Source: J Neuroeng Rehabil. 2025 Jan 20;22:7. doi: 10.1186/s12984-024-01489-5 (PMC11748334; doi:10.1186/s12984-024-01489-5)
Supplement: Supplementary file 1 — Additional file 1. [file 12984_2024_1489_MOESM1_ESM.docx]

### Additional files

#### Additional file 1. Study 2. List of questions used for the semi-structured interviews.

| **Topics** | **Questions** | **Sub-questions** |
| --- | --- | --- |
| Roles, responsibilities, tasks | Can you describe to me your job? | *How many years of experience do you have in your field?*  *What are your primary responsibilities and tasks?* |
| Diagnosis, prognosis, treatment design | What is essential to evaluate new patients? | *What do you observe in patients?*  *What do you need to know about a patient?*  *Are factors like patients’ (type and level of) impairment/injury, personality traits, educational level, gender, marital status, socio-economic background, low/high income, nationality, language, etc. influencing the design of the treatment plan? To what extent? Why?*  *Is there any relationship between socioeconomic background, personality traits, low/high income with the ability of patients to take care of their health?*  *To what extent does personality influence the design of the treatment therapy?* |
|  | What type of exams do you execute? | *How do you assess the patients’ cognitive abilities?* |
|  | How do you deal with a patient thinking/feeling like he/she cannot make it? | |
|  | What are the differences between Group 1, 2 and 3? | |
| Therapy treatment | How does your evaluation influence the therapy treatment design? | *How do you choose the best treatment for the patient?*  *How often do you meet with a patient?* |
|  | How is the assessment translated into activities of daily living (ADL)? | |
|  | How to be always effective in the treatment? | *What do patients need to learn in rehabilitation?*  *How does learning become effective with them?*  *How do you train the patients’ cognitive abilities?*  *How do you decide on the tasks patients must execute?*  *What to present to patients? And when?*  *What makes learning difficult for patients?* |
|  | What types of exercises are given to patients for training their cognitive abilities? | *What do you think are the most effective exercises to train their cognitive abilities?*  *How do impaired cognitive and executive abilities impact the therapy strategy?*  *Are lack of Motivation, Self-awareness, and Attention the main cognitive issues within patients with a stroke?*  *Are there any cognitive strategies you teach or employ to help and support patients?*  *How do you modulate the complexity of a task?*  *What elements do you take in consideration for reducing patients’ cognitive load during task execution?*  *How do you ensure patients are improving their cognitive abilities?*  *What additional tasks make the exercises more difficult? (e.g., talking while walking)* |
|  | How do you measure cognitive load? | *Can the cognitive load be detected/measured by changes in the patient’s behaviour?*  *If so, can you tell me more about that?* |
| Patients’ challenges, needs, motivation | What do you think are the main challenges for a patient suffering from a stroke during rehabilitation? Why? | *What do you think patients need?*  *What do you think motivates patients the most?*  *How do you think patients feel during rehabilitation?* |
|  | Have you observed any fluctuation of motivation in patients during their rehabilitation? | *Can you tell me more about it?*  *What do you think the motivation fluctuations in patients during their rehabilitation depend on?* |
|  | Why must patients self-evaluate themselves after any exercise? | |
| Users’ involvement | To what extent the involvement of family members is important for rehabilitation? Why? | *Is the therapy treatment of a patient living alone different from others having a family?* |
| IVR-based rehabilitation | Do you have any previous experience with VR or IVR systems for rehabilitation? | *If so, can you tell me more about that?*  *What do you think about patients with a stroke using VR/IVR?*  *Would they be willing to wear a VR headset to practice?*  *Would they be willing to practice with a robot arm in VR?* |
|  | What do you think it is possible to do with IVR in rehabilitation? Why? | *What tasks would you create for training patients’ cognitive abilities more effectively?*  *What characteristics should a VE have to promote motor re-learning and neuroplasticity?*  *What would you like to have? to see? to hear?*  *In your opinion, what could increase motivation?* |
| General | Do you know any association of former patients suffering from a stroke? | |

####

#### Additional file 2. Study 3. Online questionnaire.

| 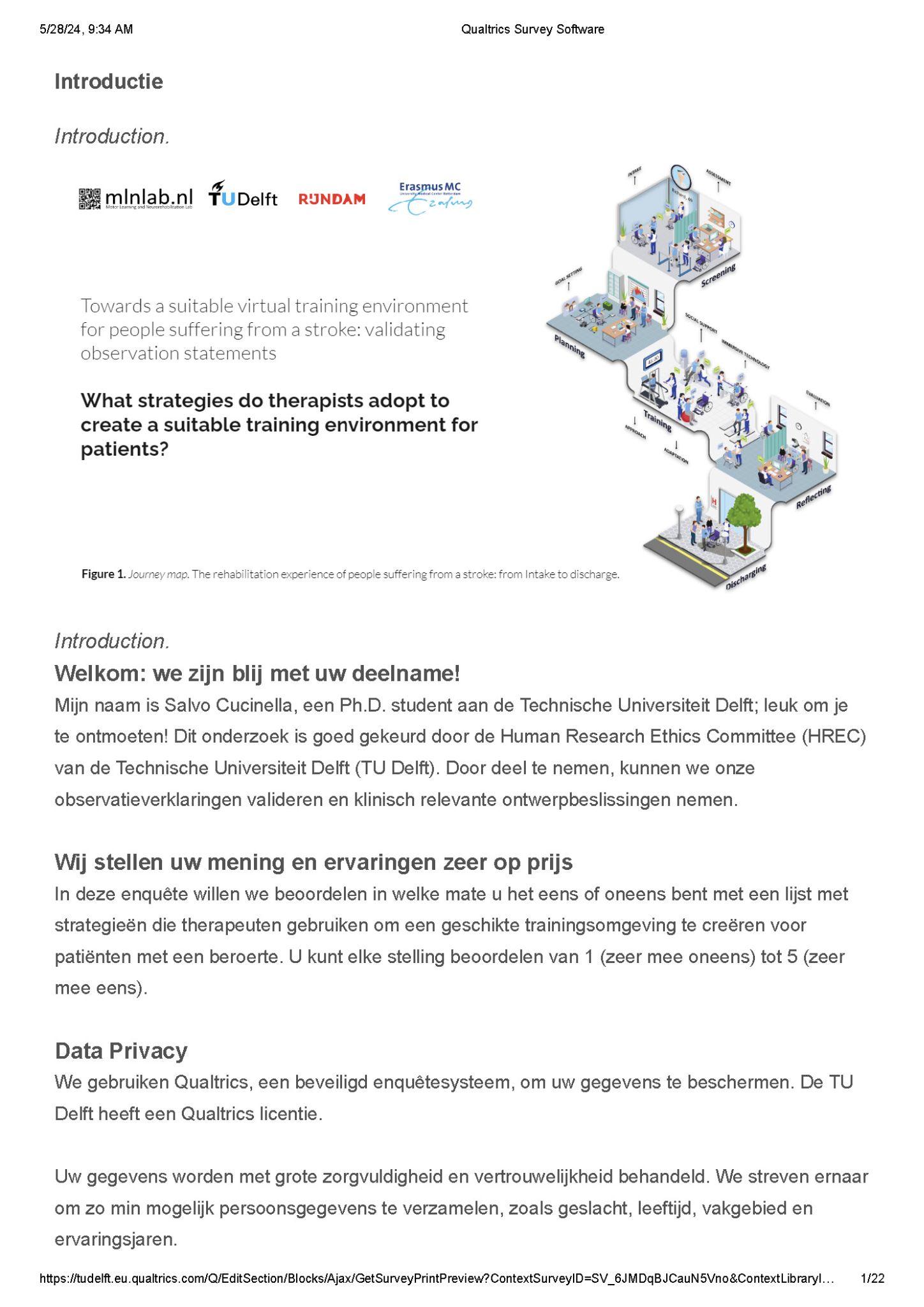 |
| --- |
| 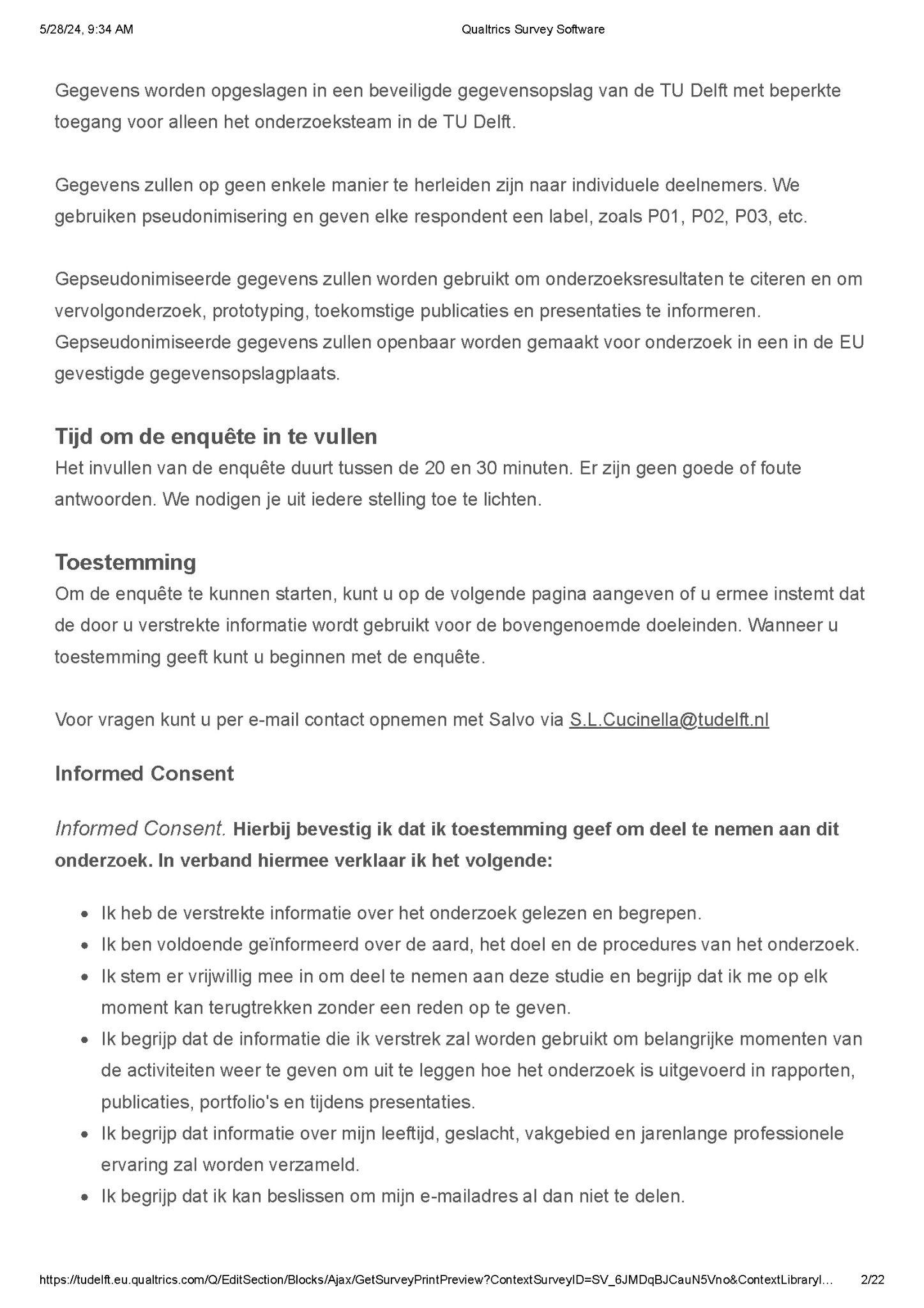 |
| 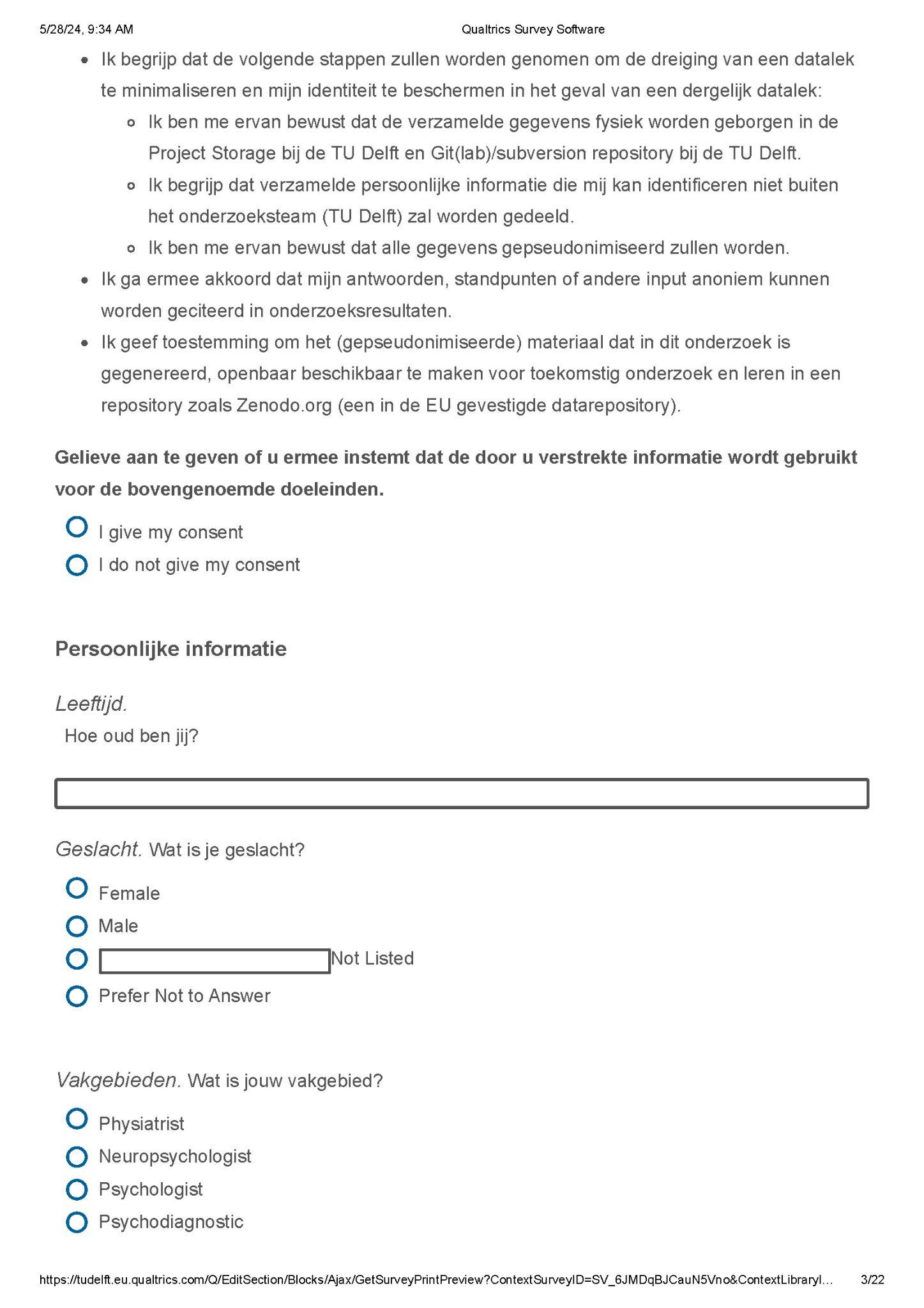 |
| 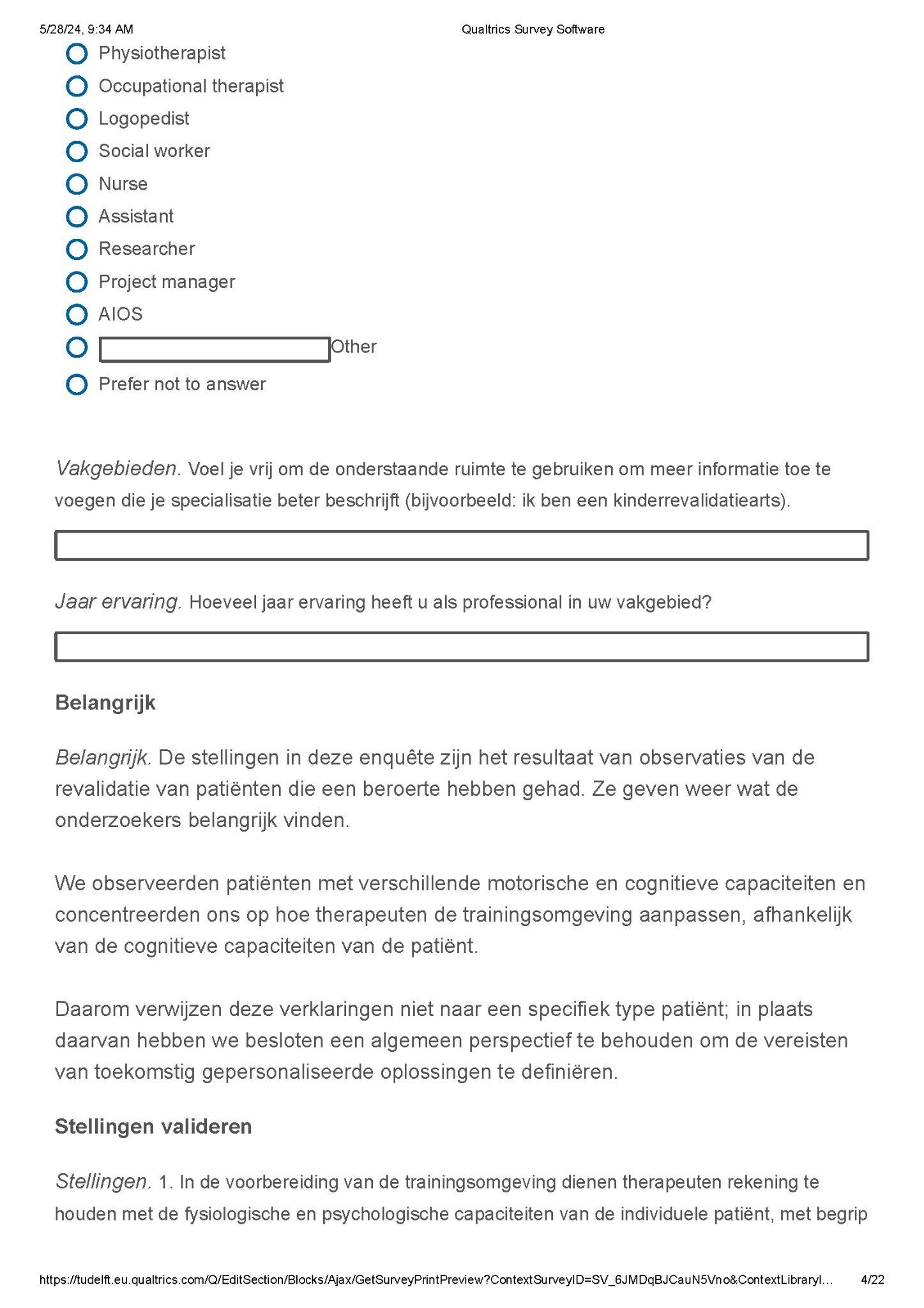 |
| 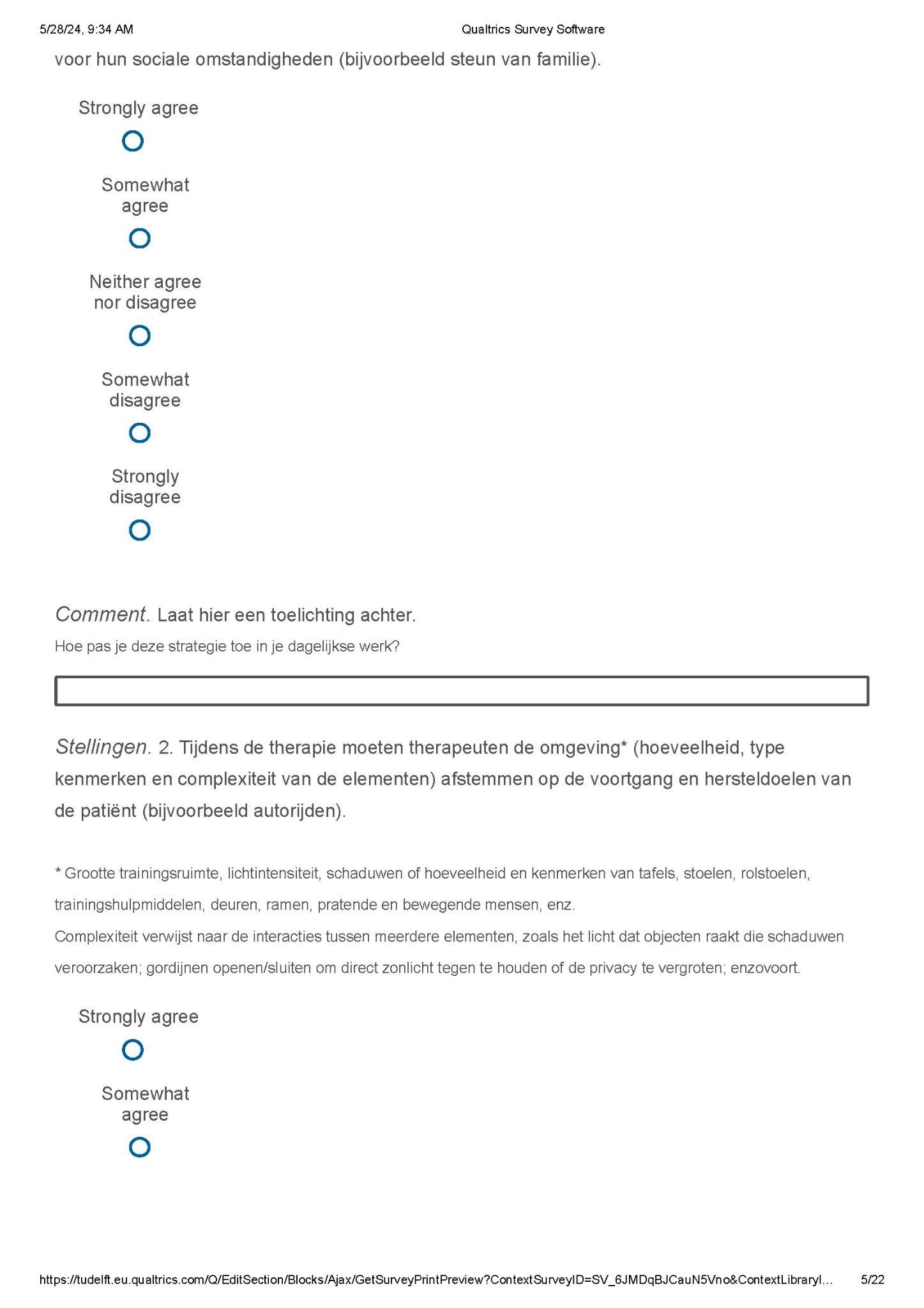 |
| 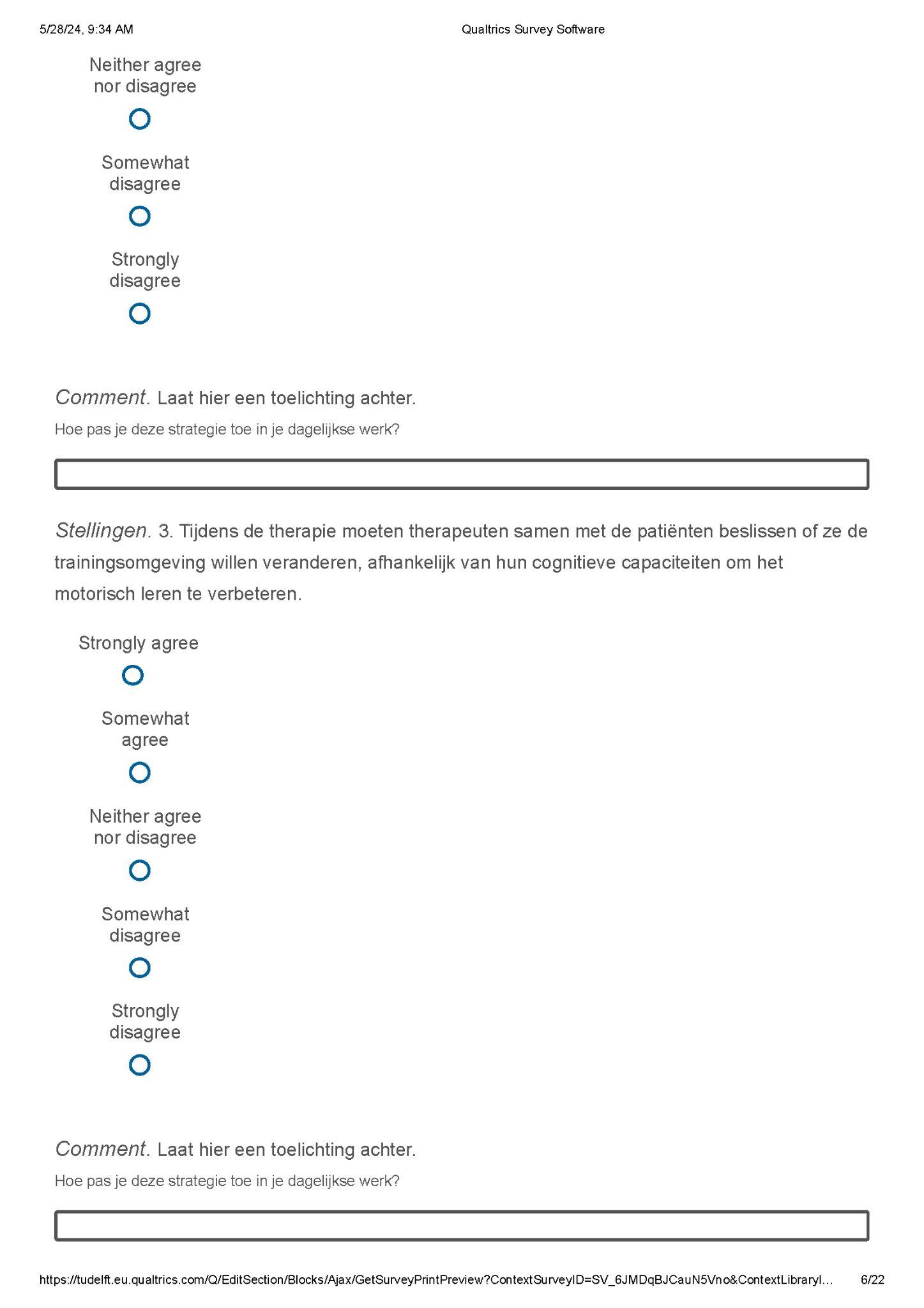 |
| 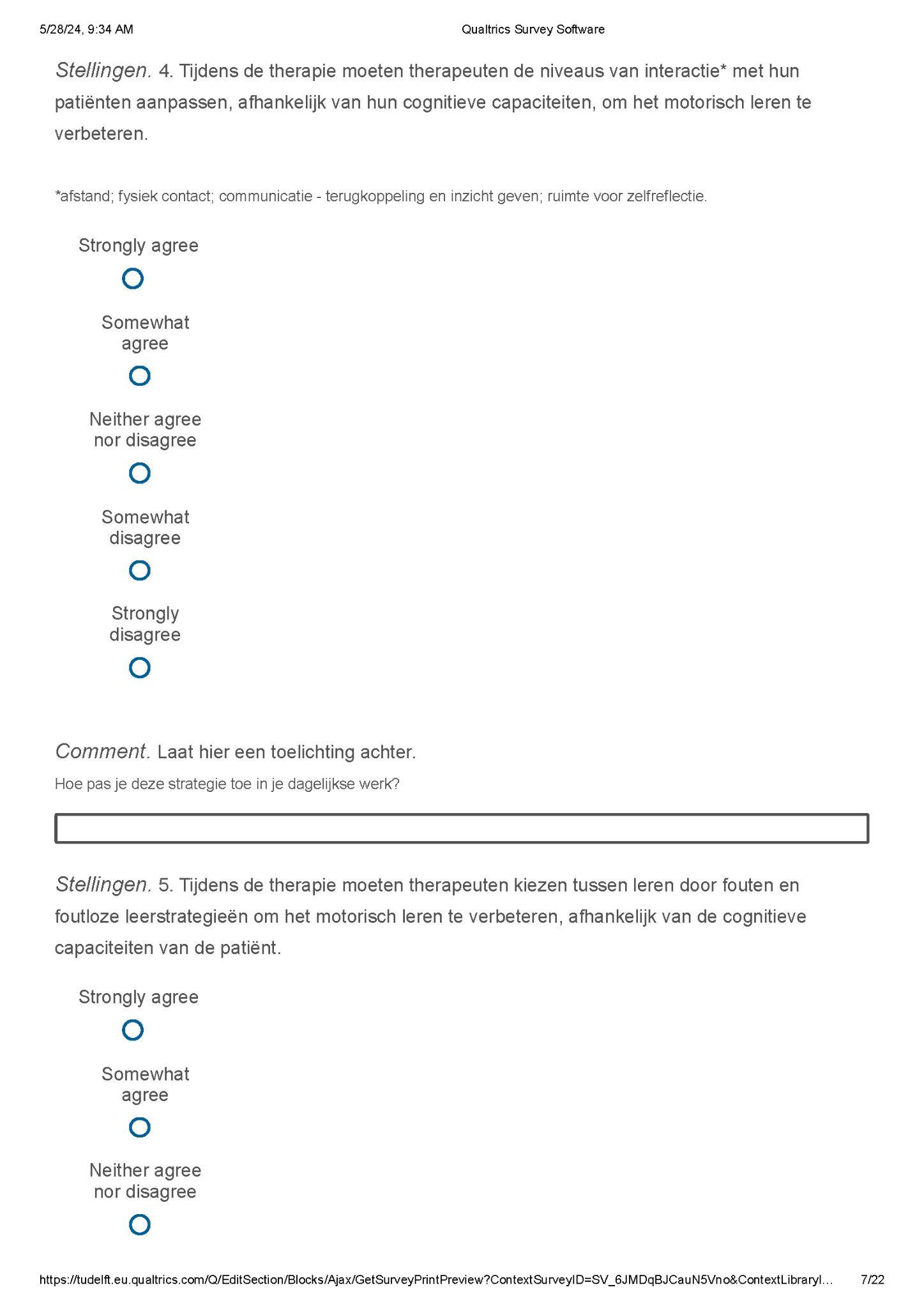 |
| 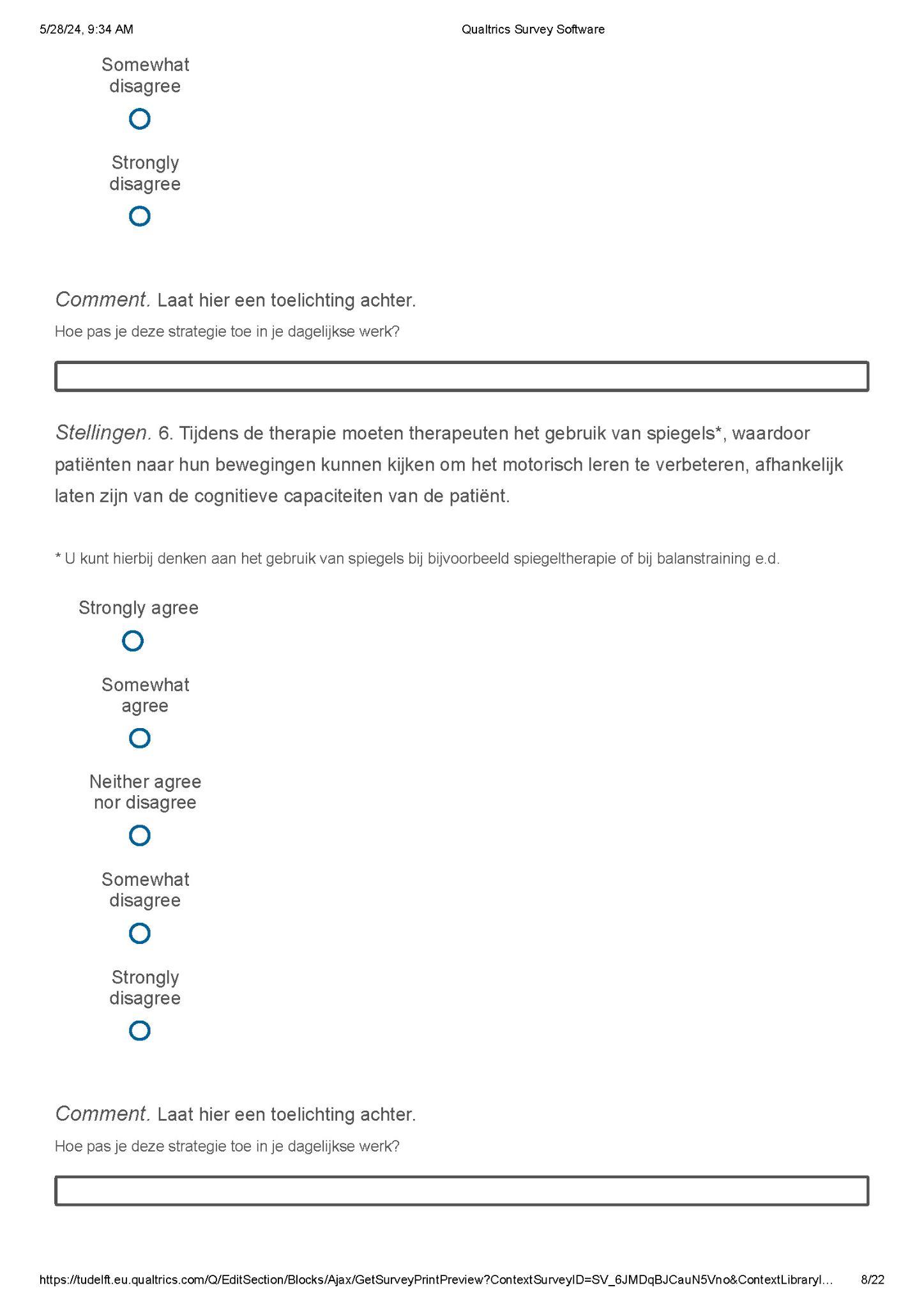 |
| 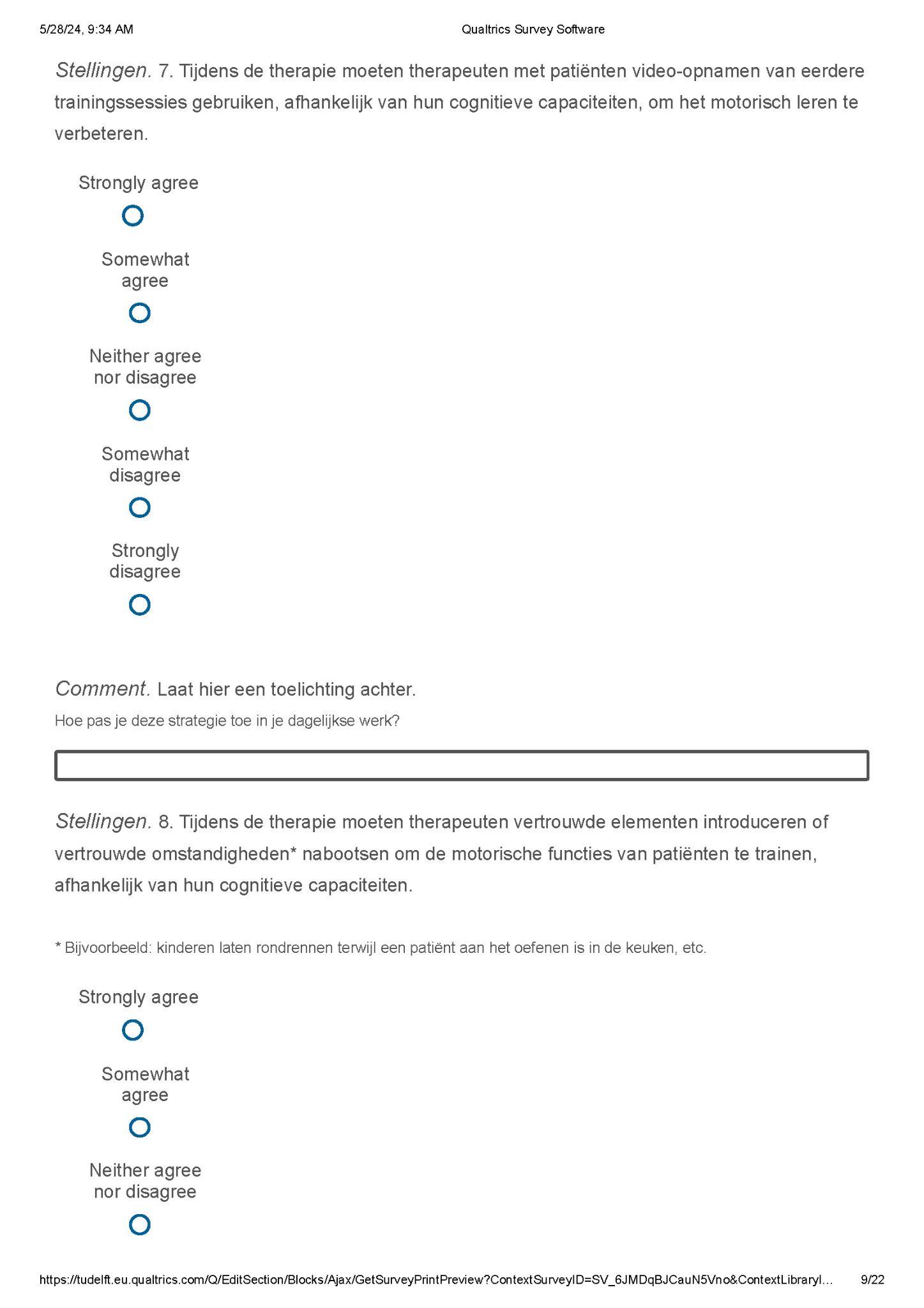 |
| 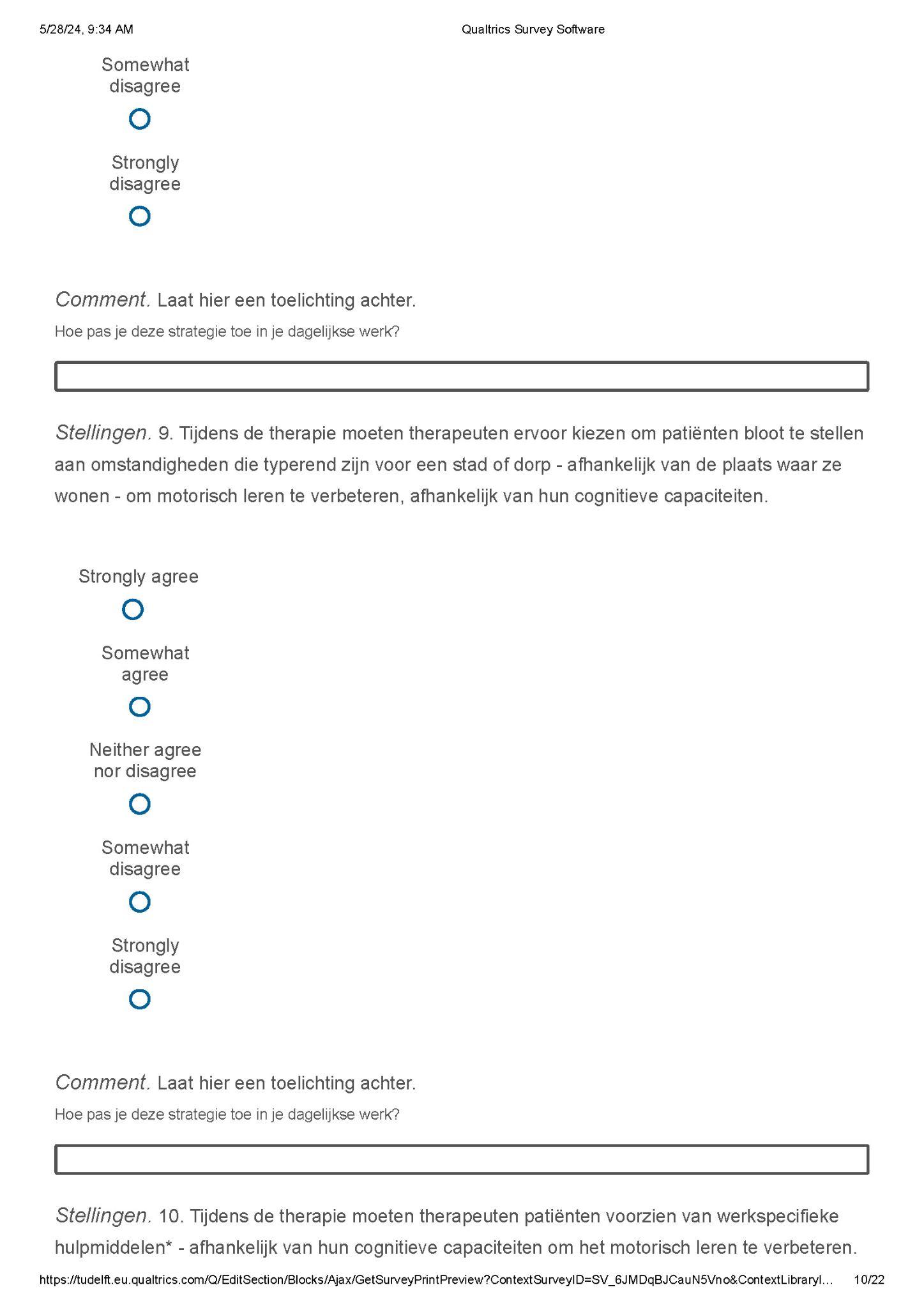 |
| 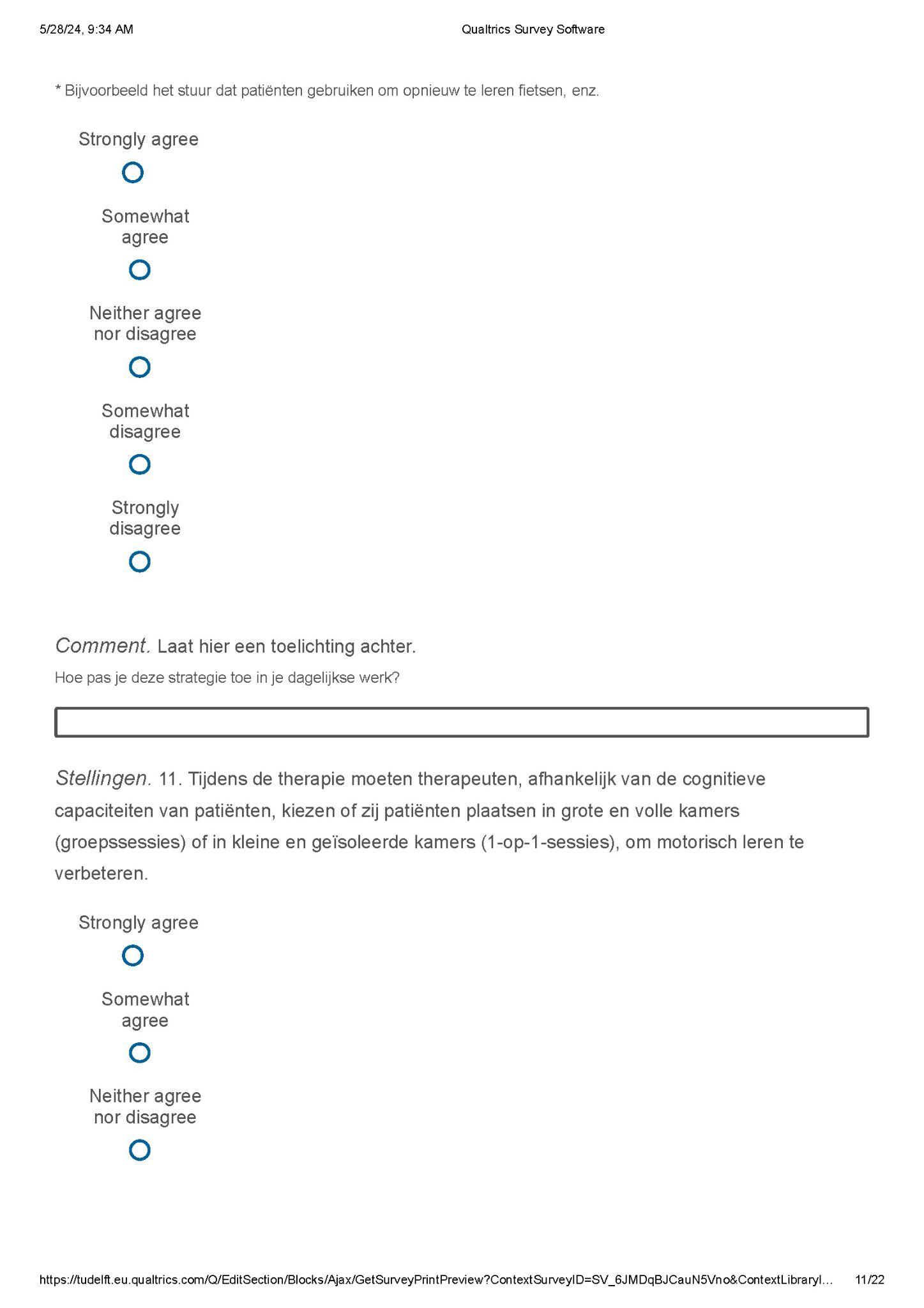 |
| 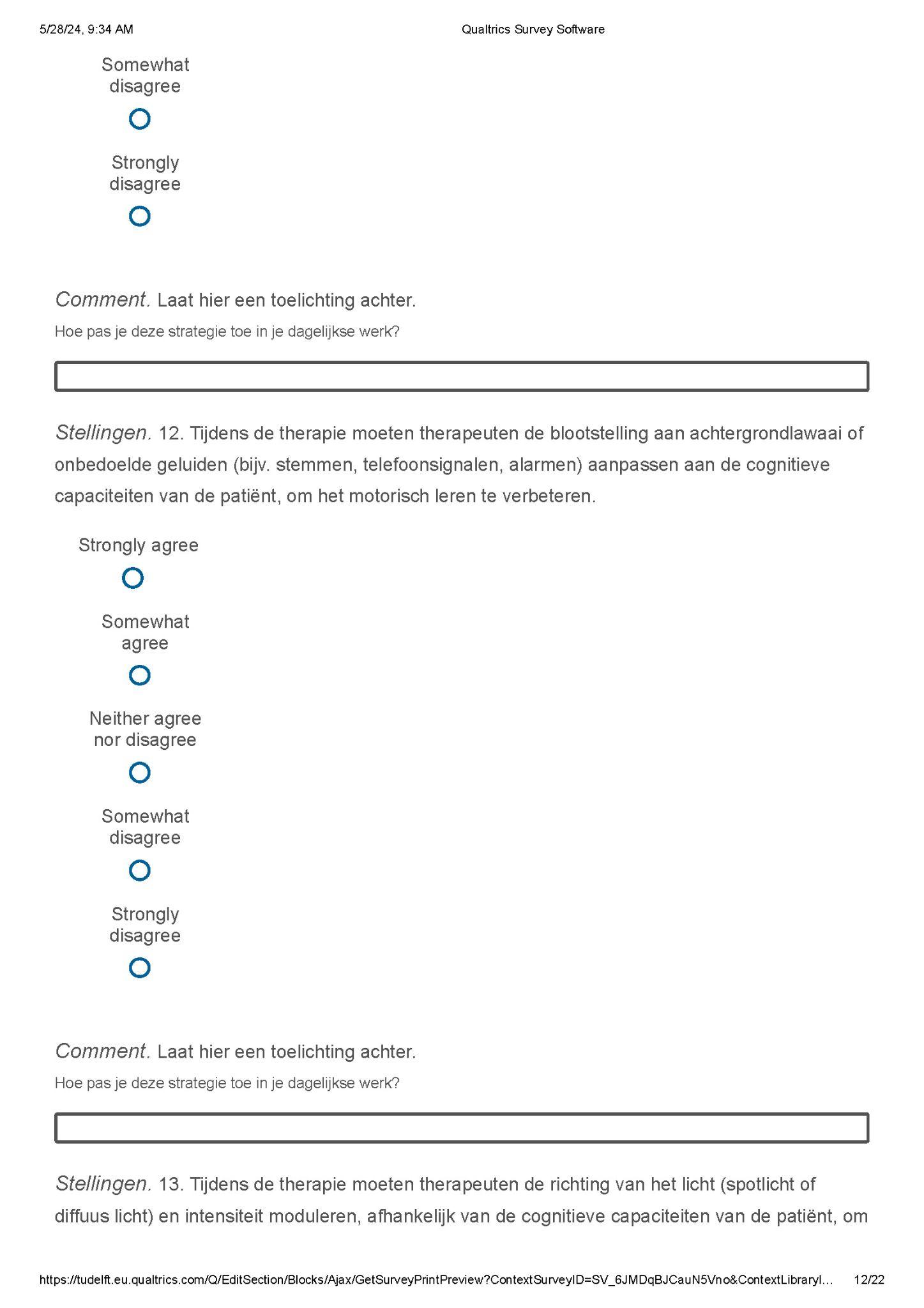 |
| 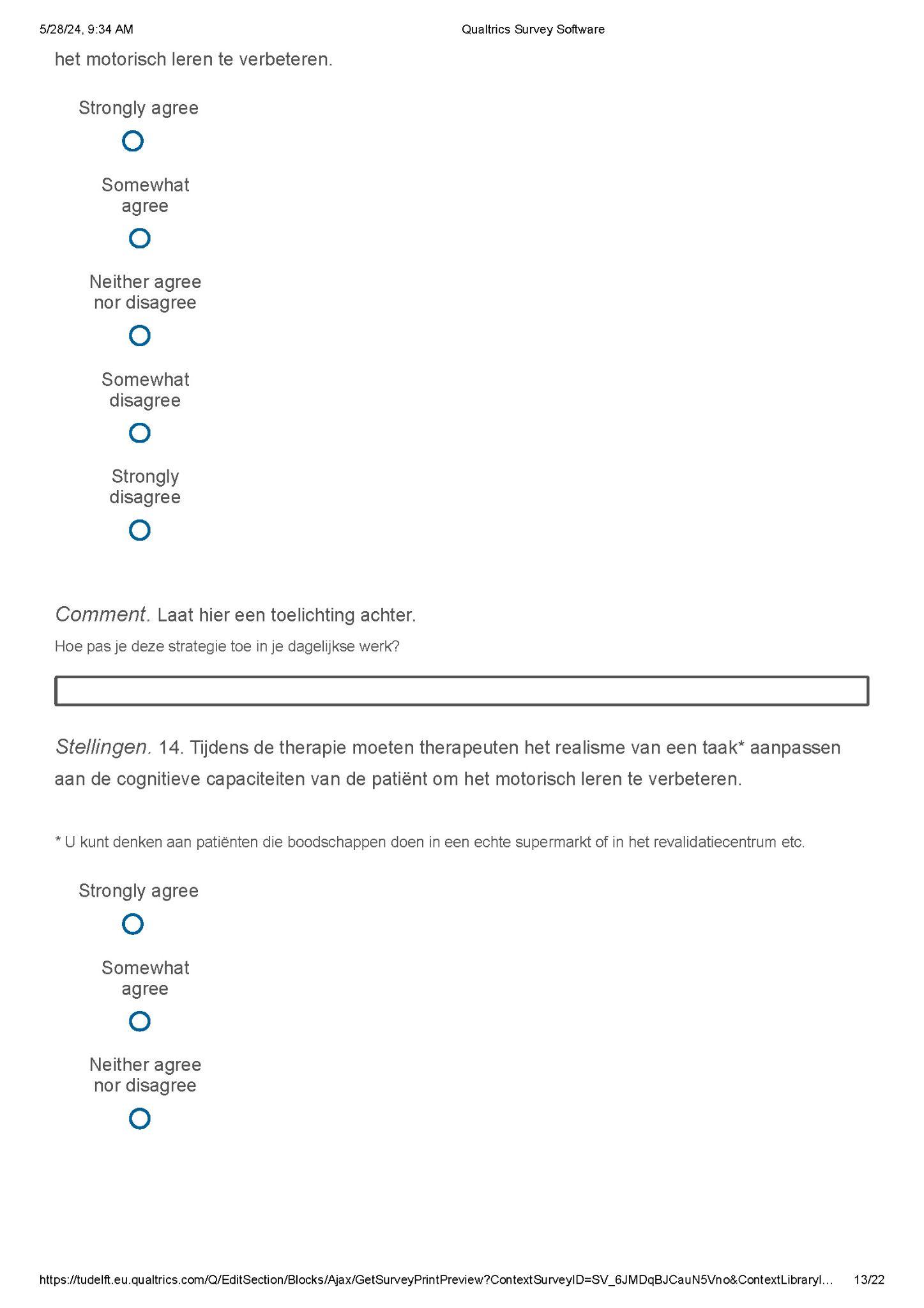 |
| 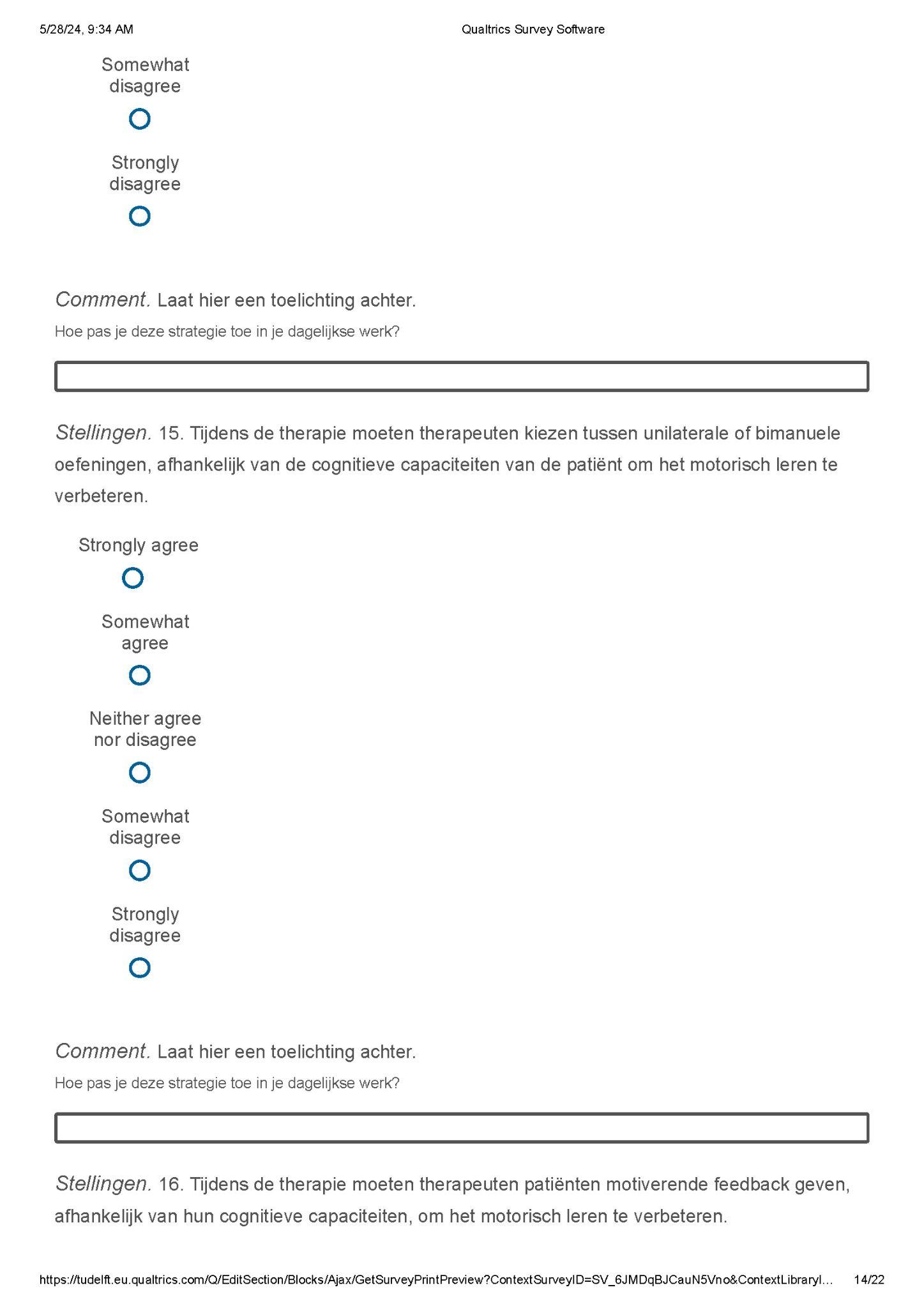 |
| 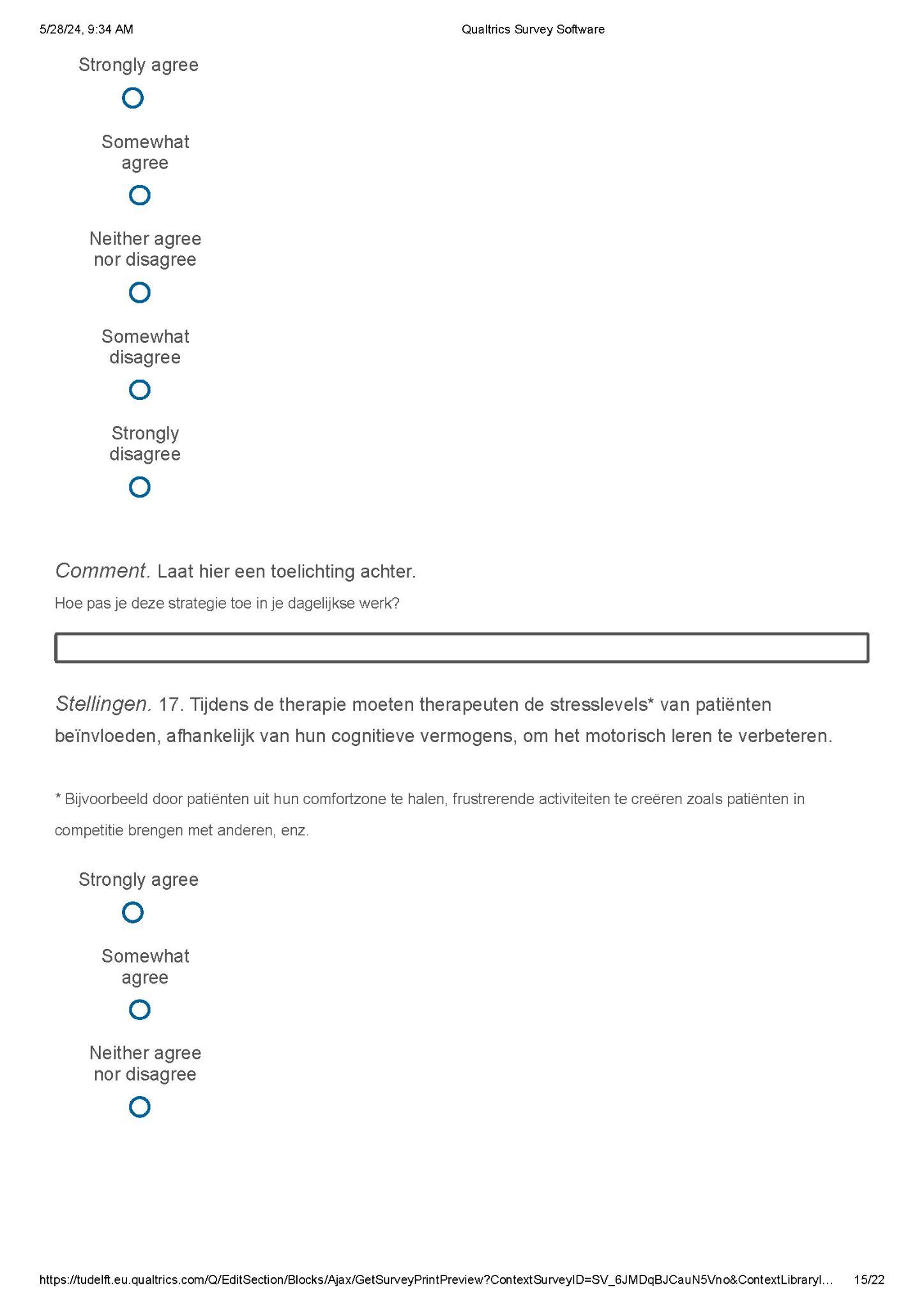 |
| 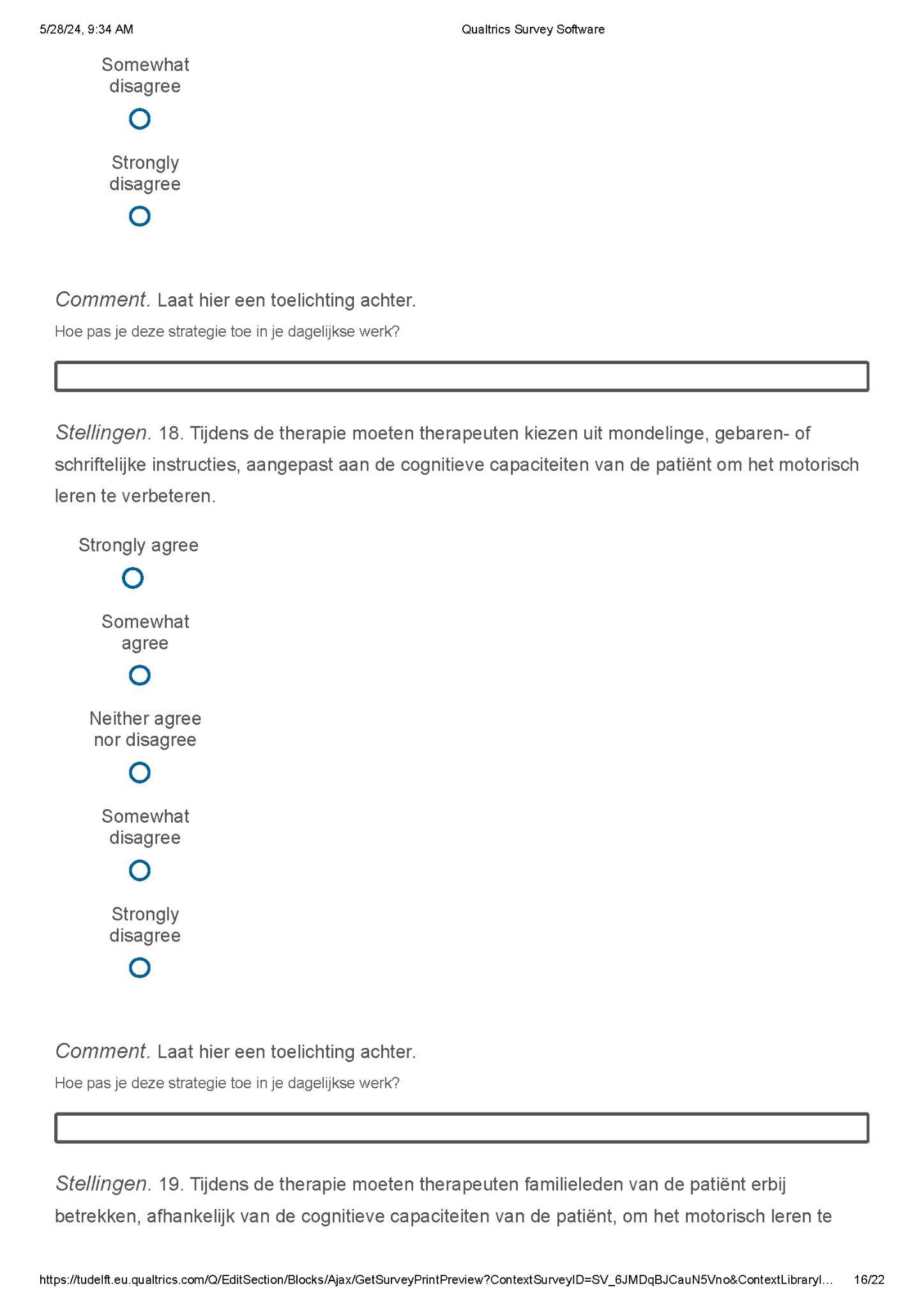 |
| 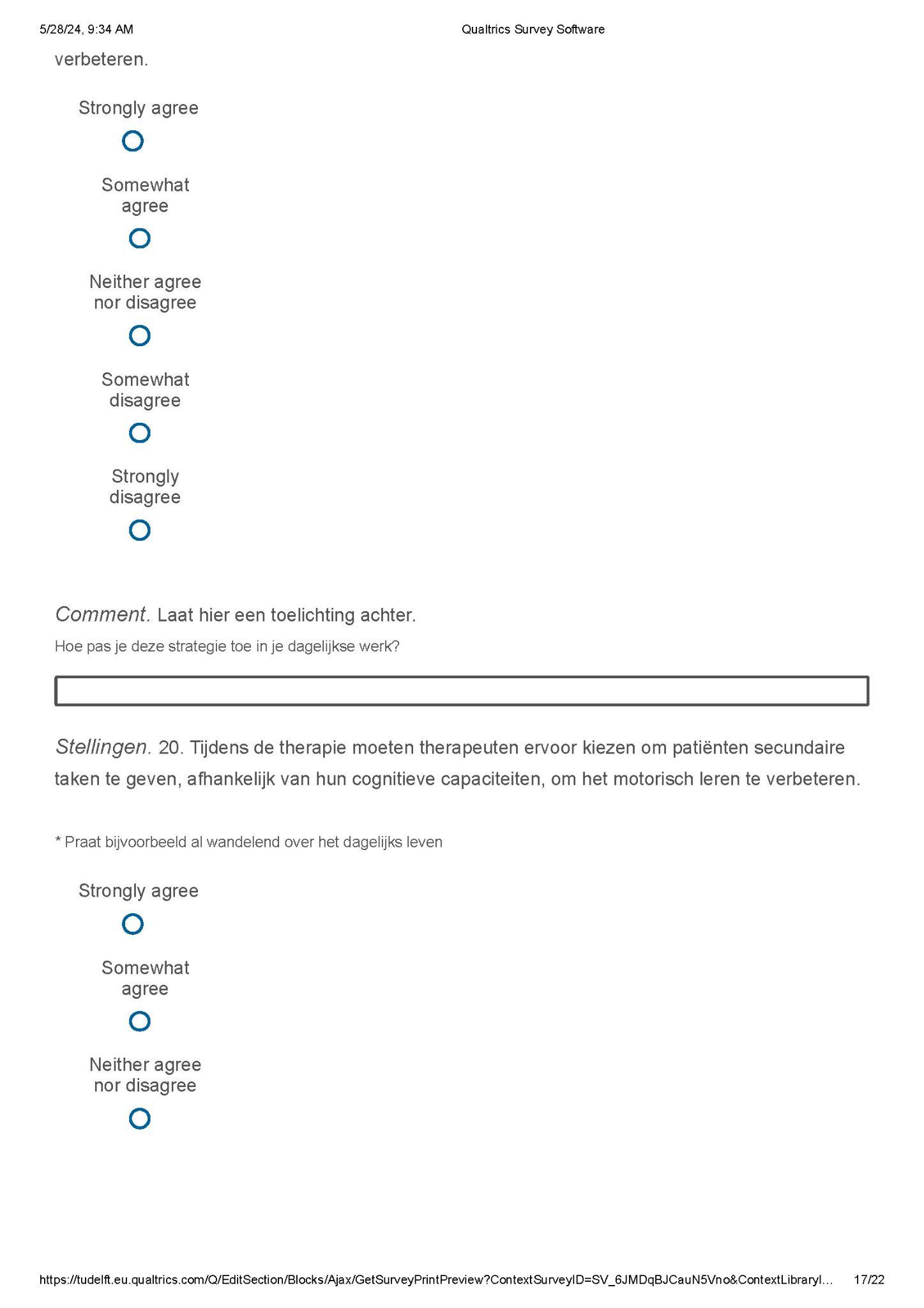 |
| 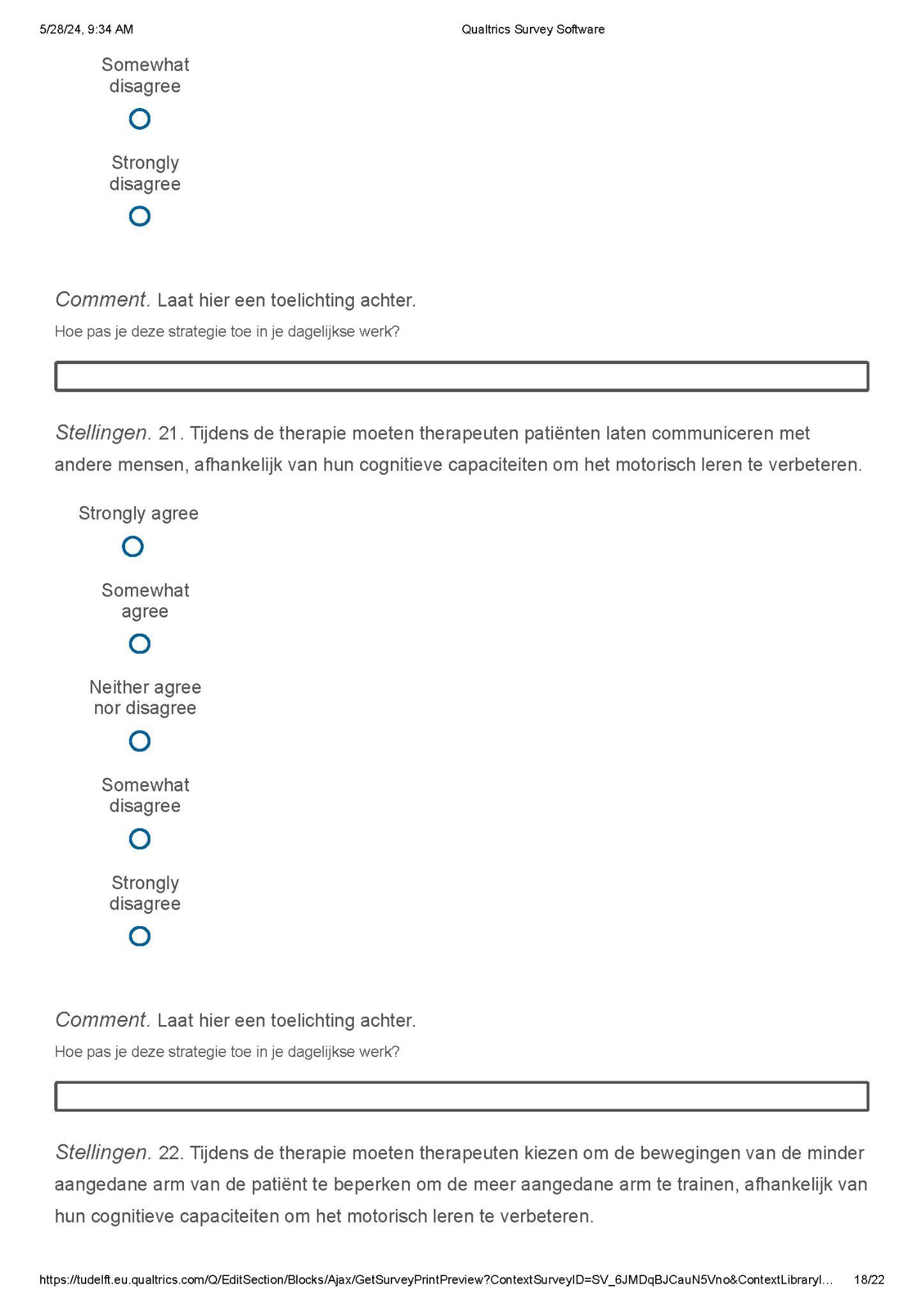 |
| 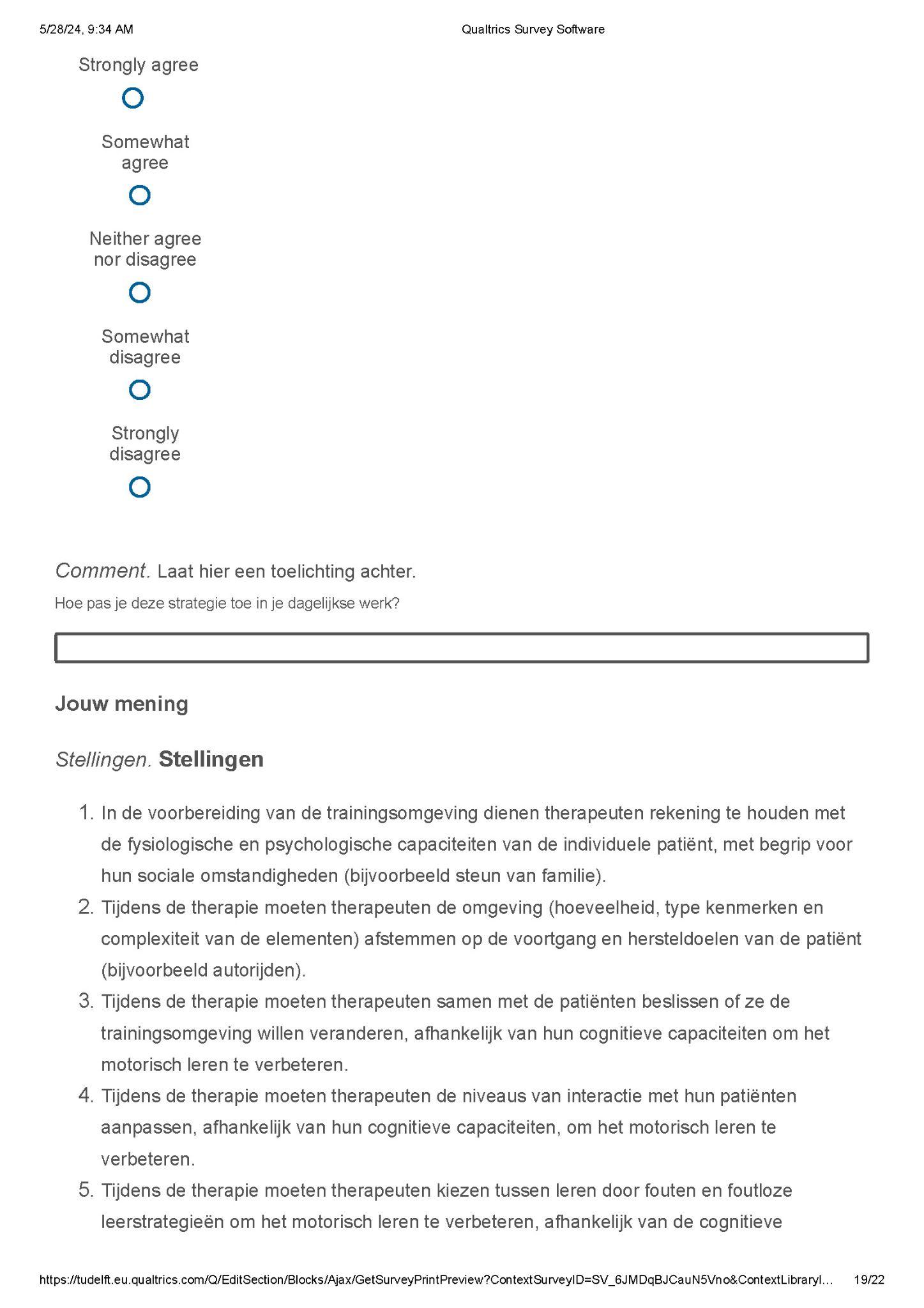 |
| 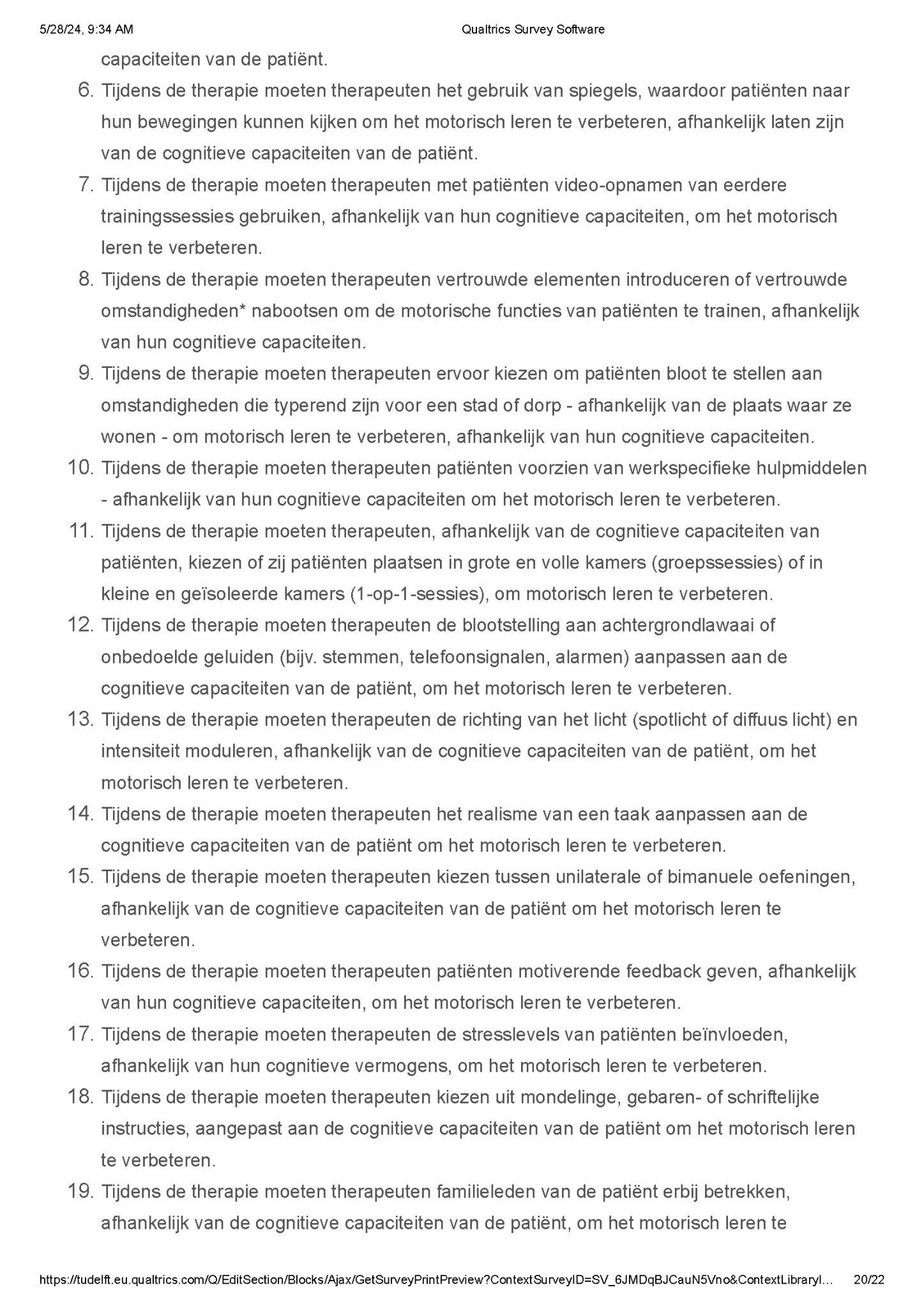 |
| 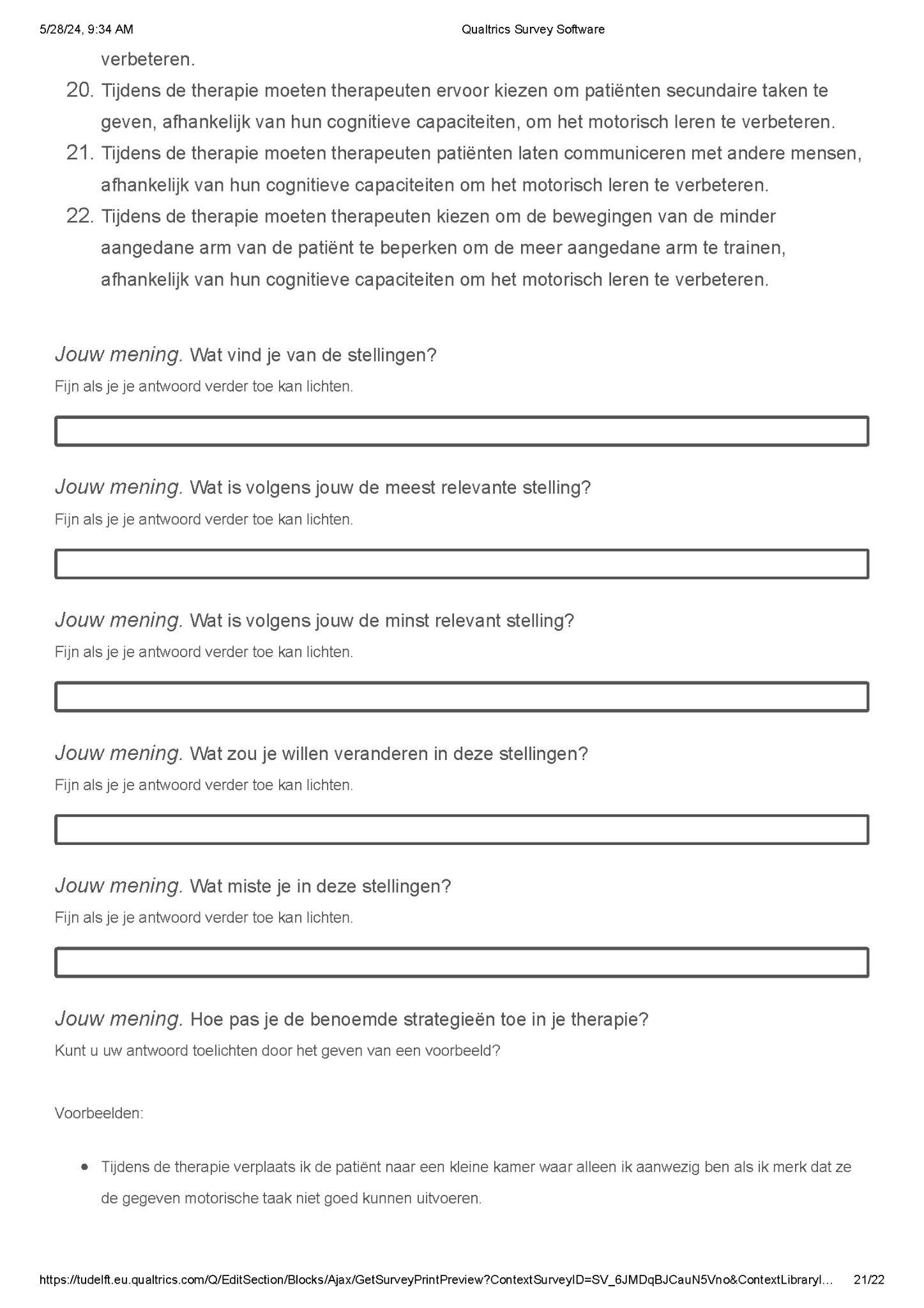 |
| 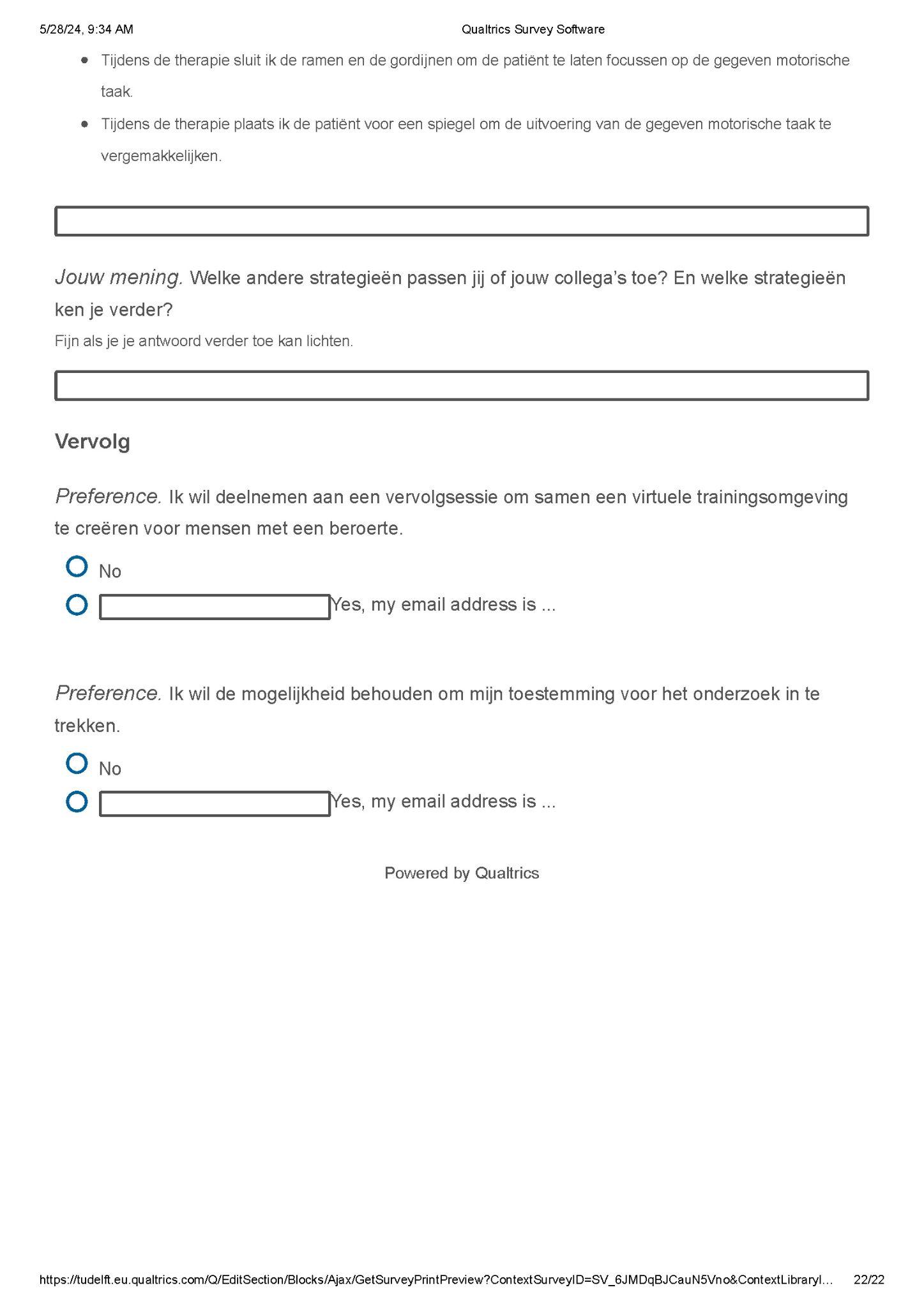 |

#### Additional file 3. Study 4. Pictures of the paper sheets used and filled in by participants with Pot-its reporting their answers.

| **Group A** | **Group B** |
| --- | --- |
| **Activity 1 - Focus group** | |
| 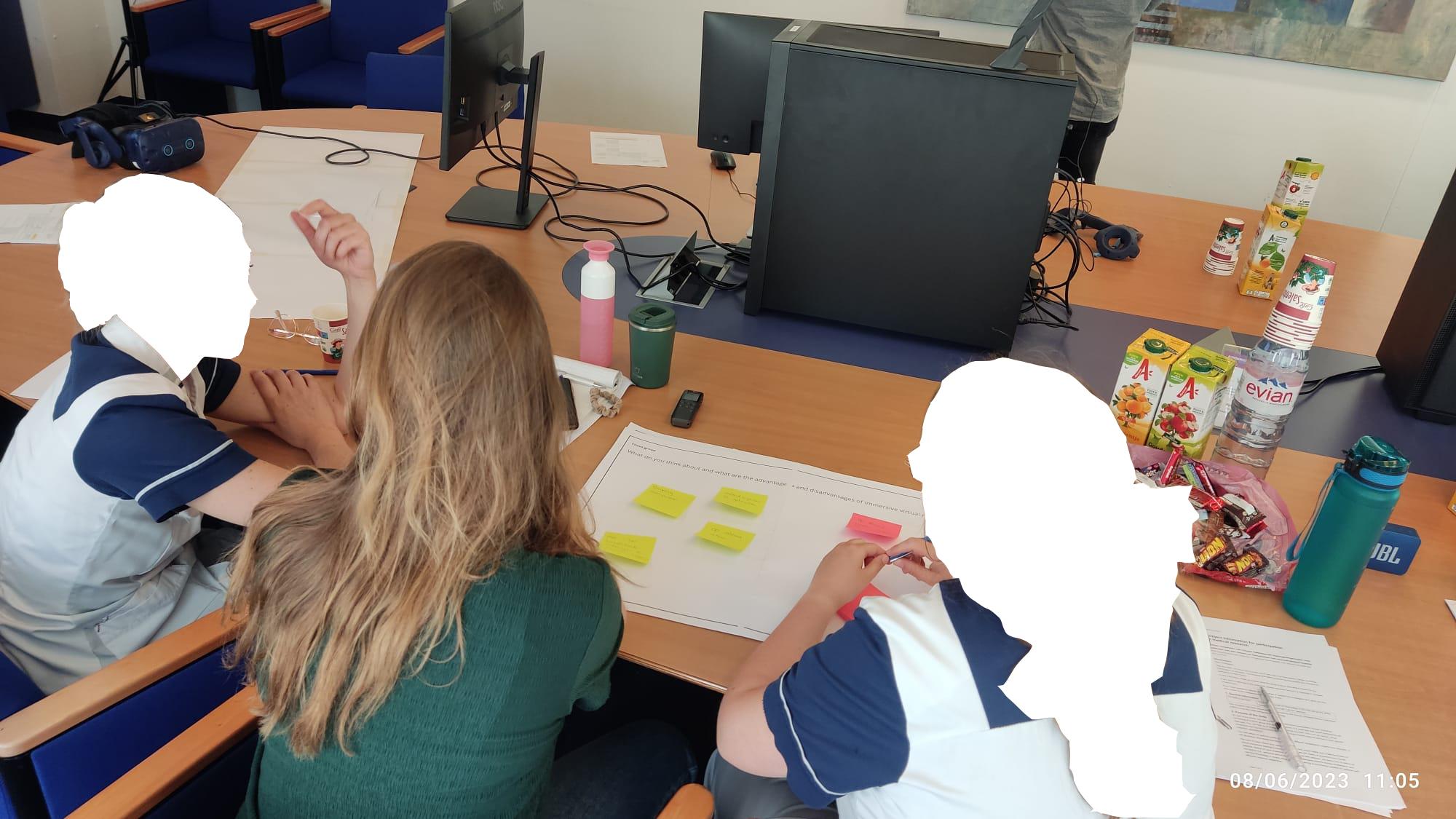 | |
| **Activity 1 - Focus group.** Participants working on the paper sheet used during the focus group. Post-its placed on top of the paper sheet report participants’ ideas. | |
| 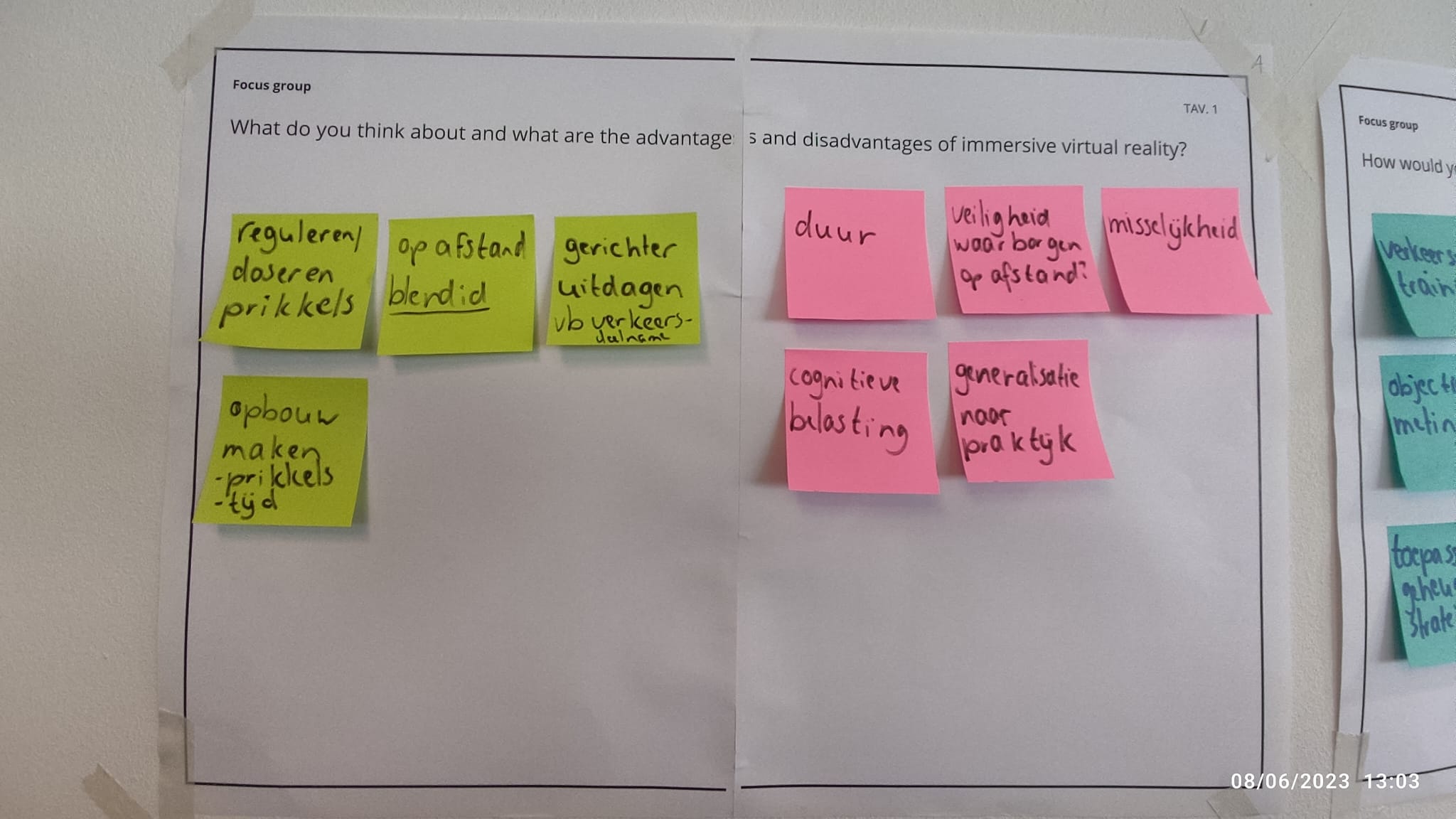 | 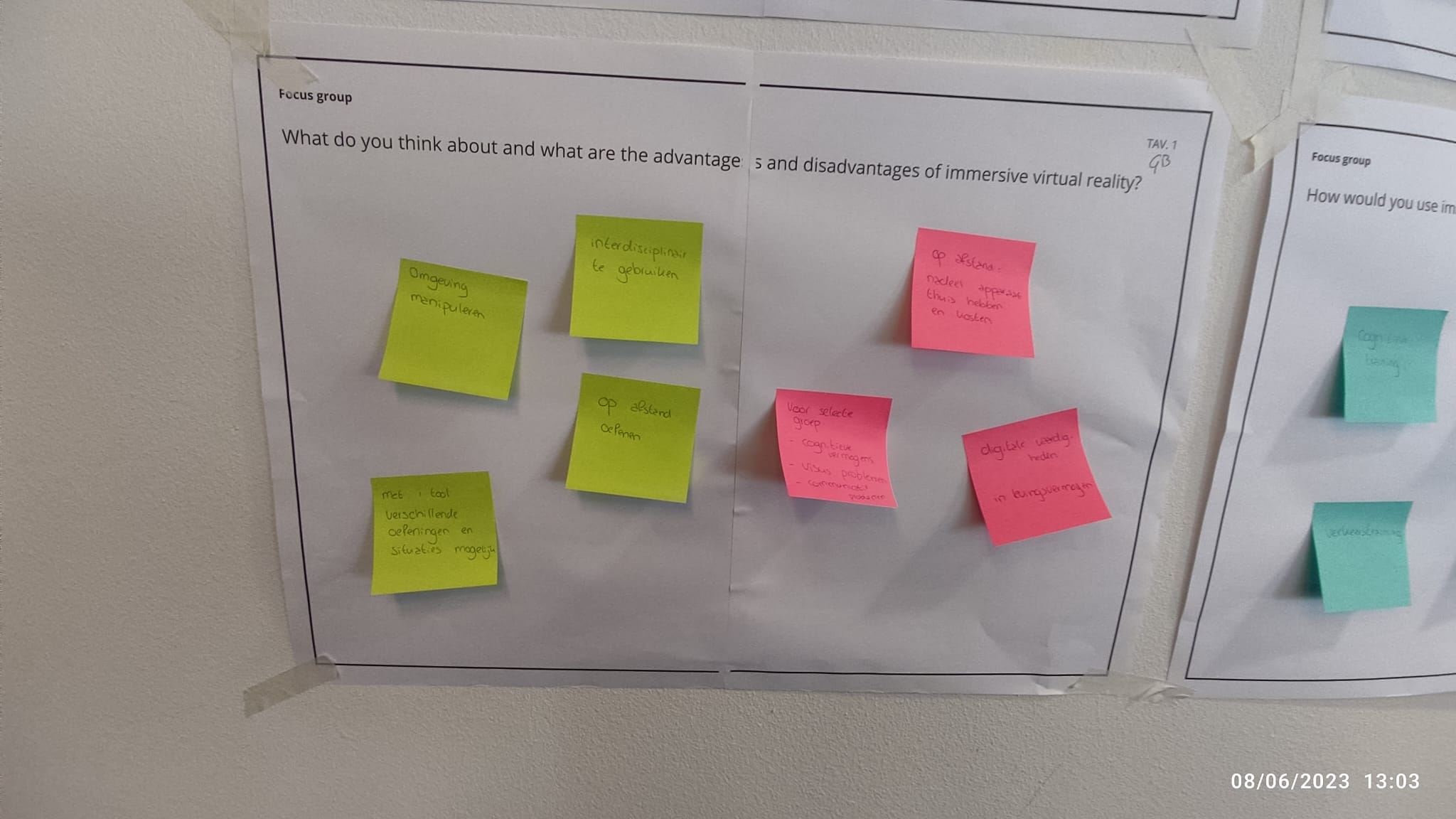 |
| **Paper sheet 1.** Question 1. *What do you think about and what are the advantages and disadvantages of immersive virtual reality?* | |
| 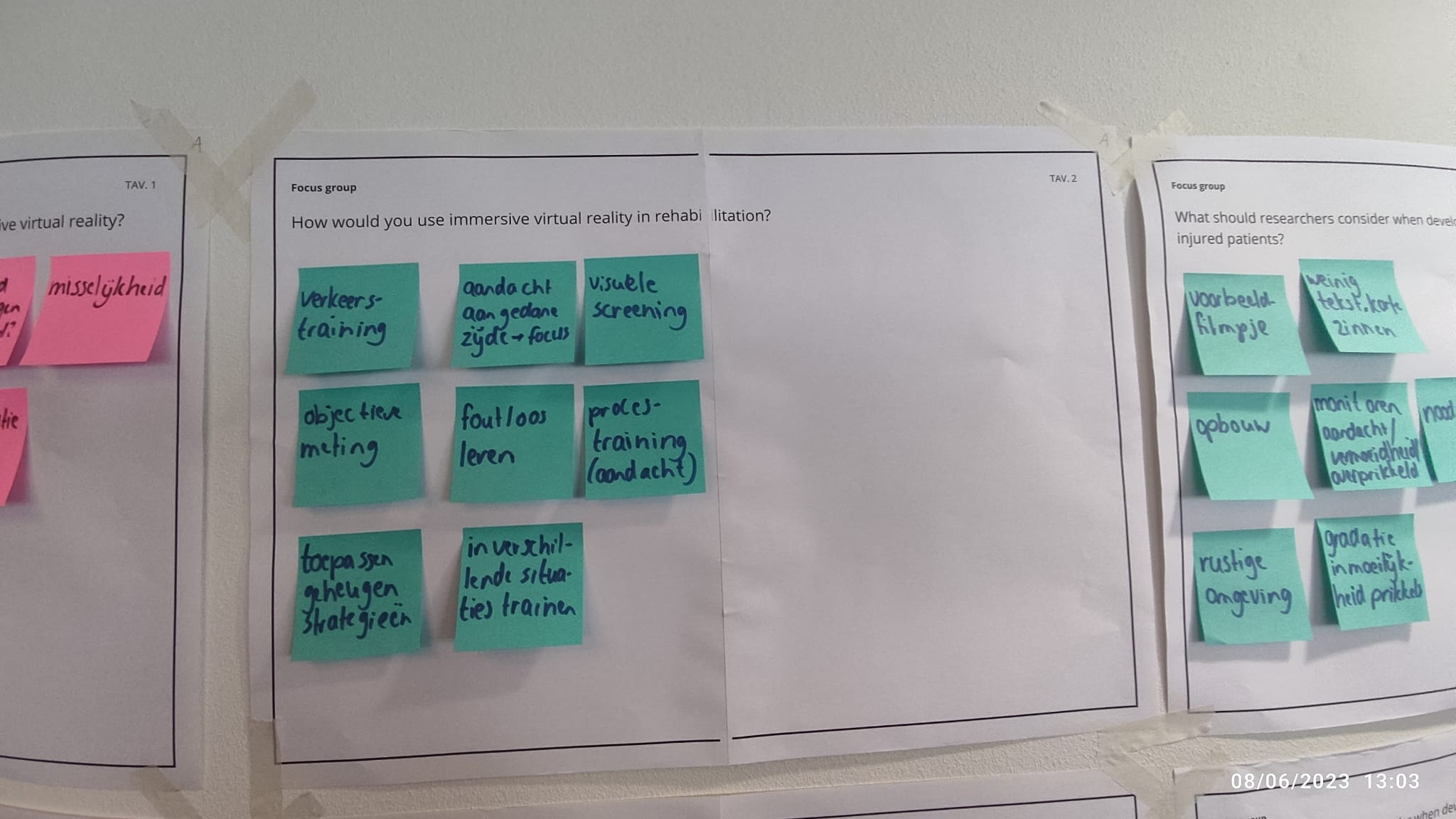 | 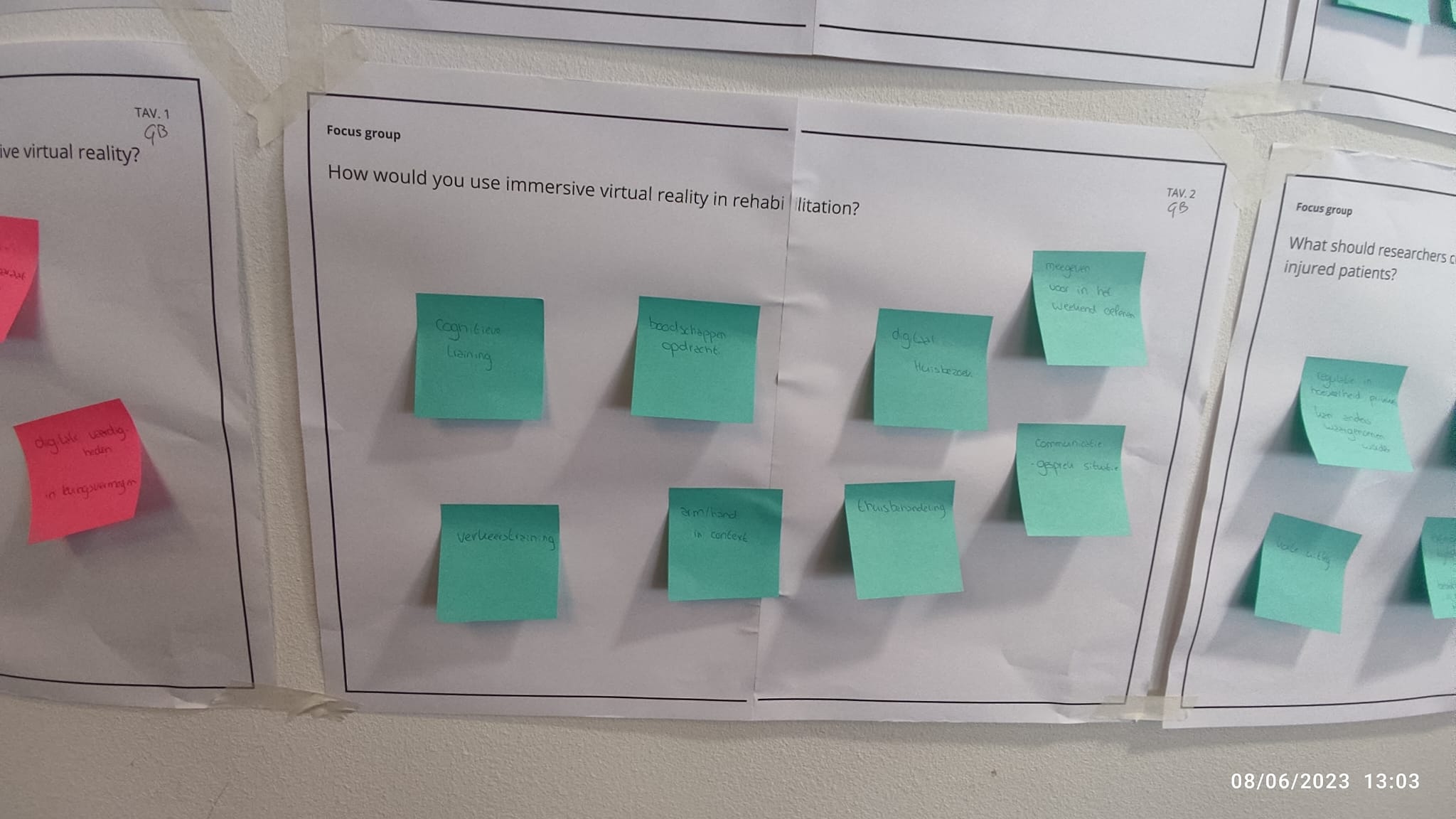 |
| **Paper sheet 2.** Question 2. *How would you use immersive virtual reality in rehabilitation?* | |
| 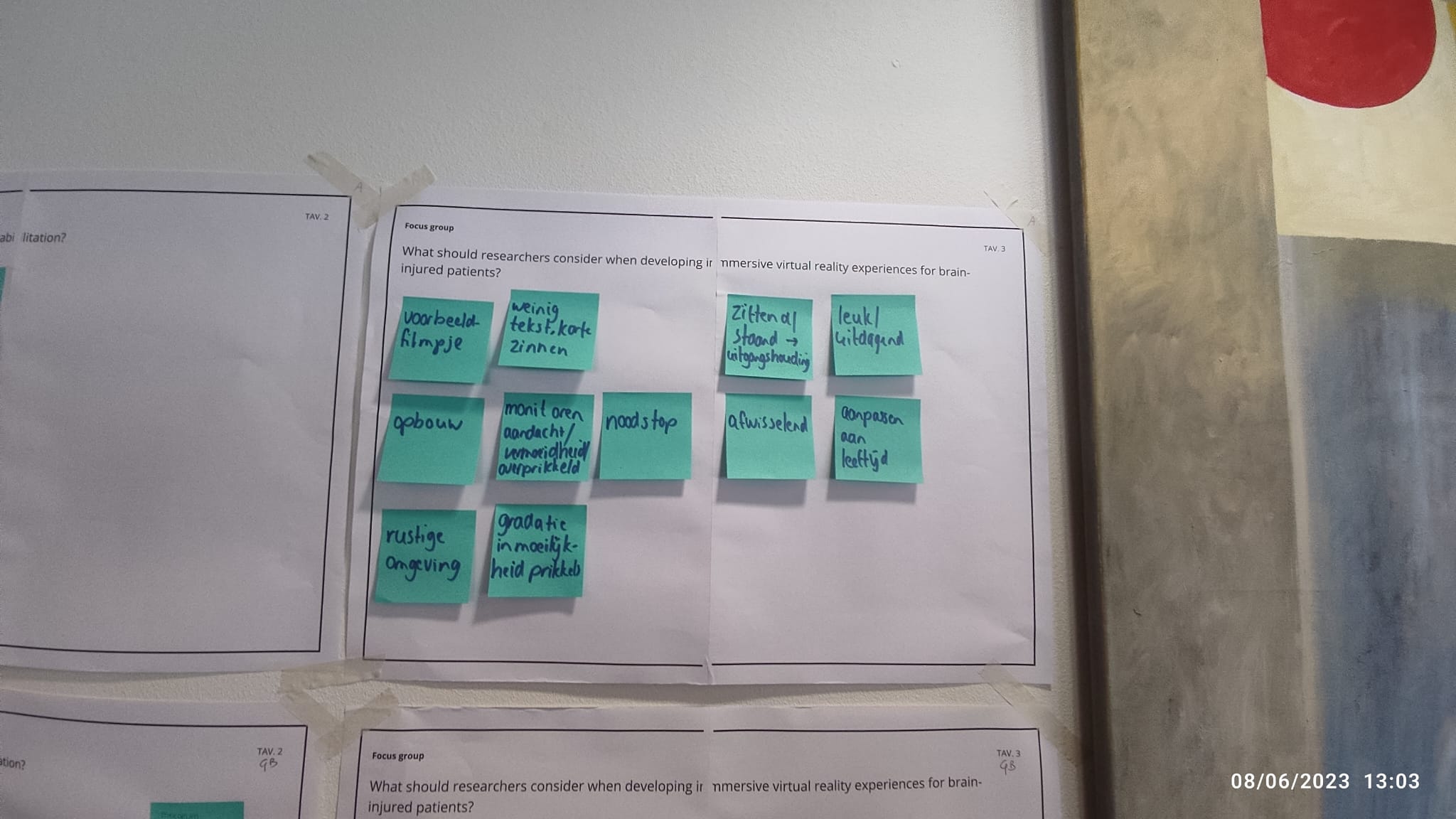 | 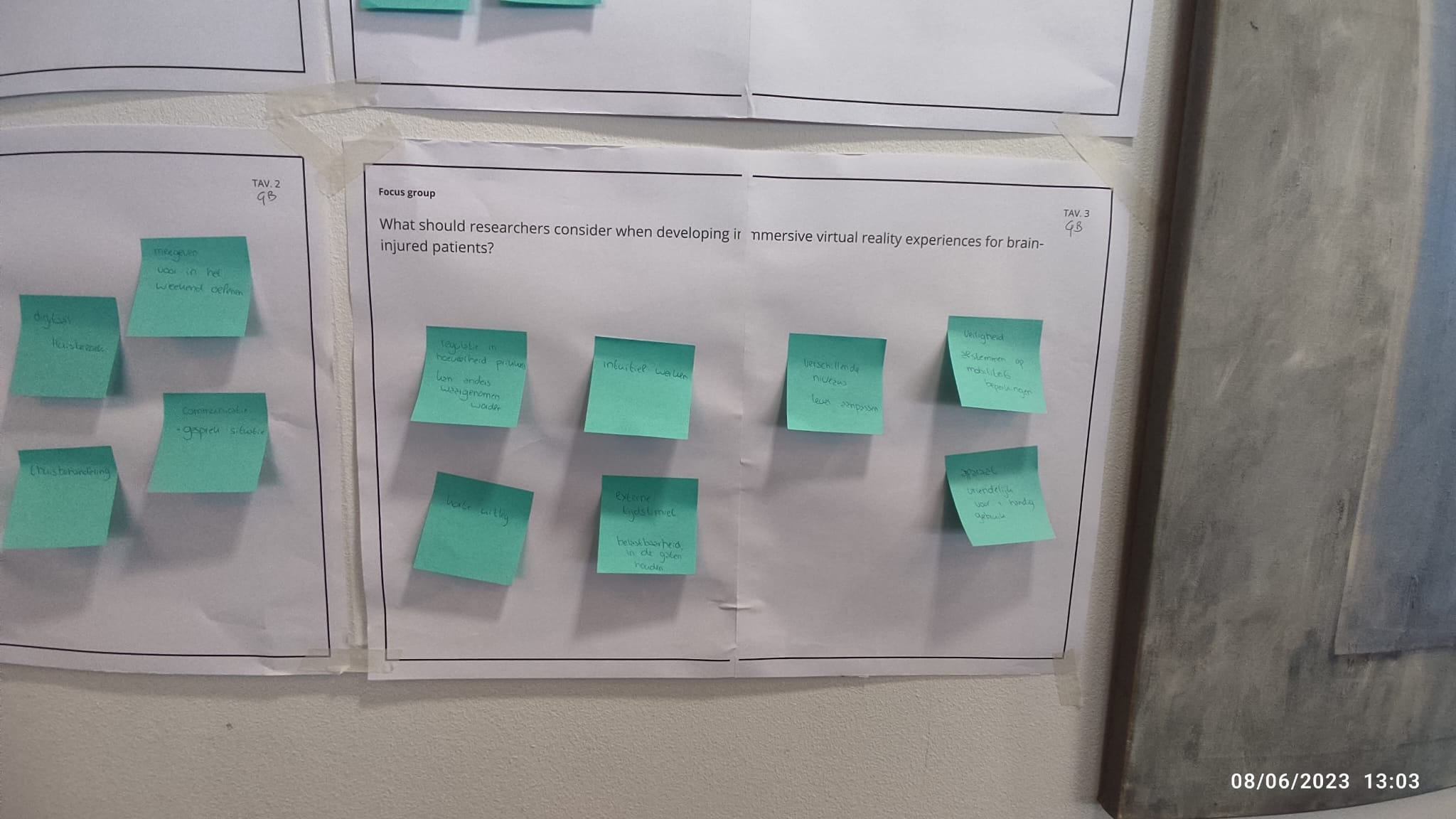 |
| **Paper sheet 3.** Question 3. *What should researchers consider when developing immersive virtual reality experiences for brain-injured patients?* | |
| **Activity 2 - Ideation session** | |
| **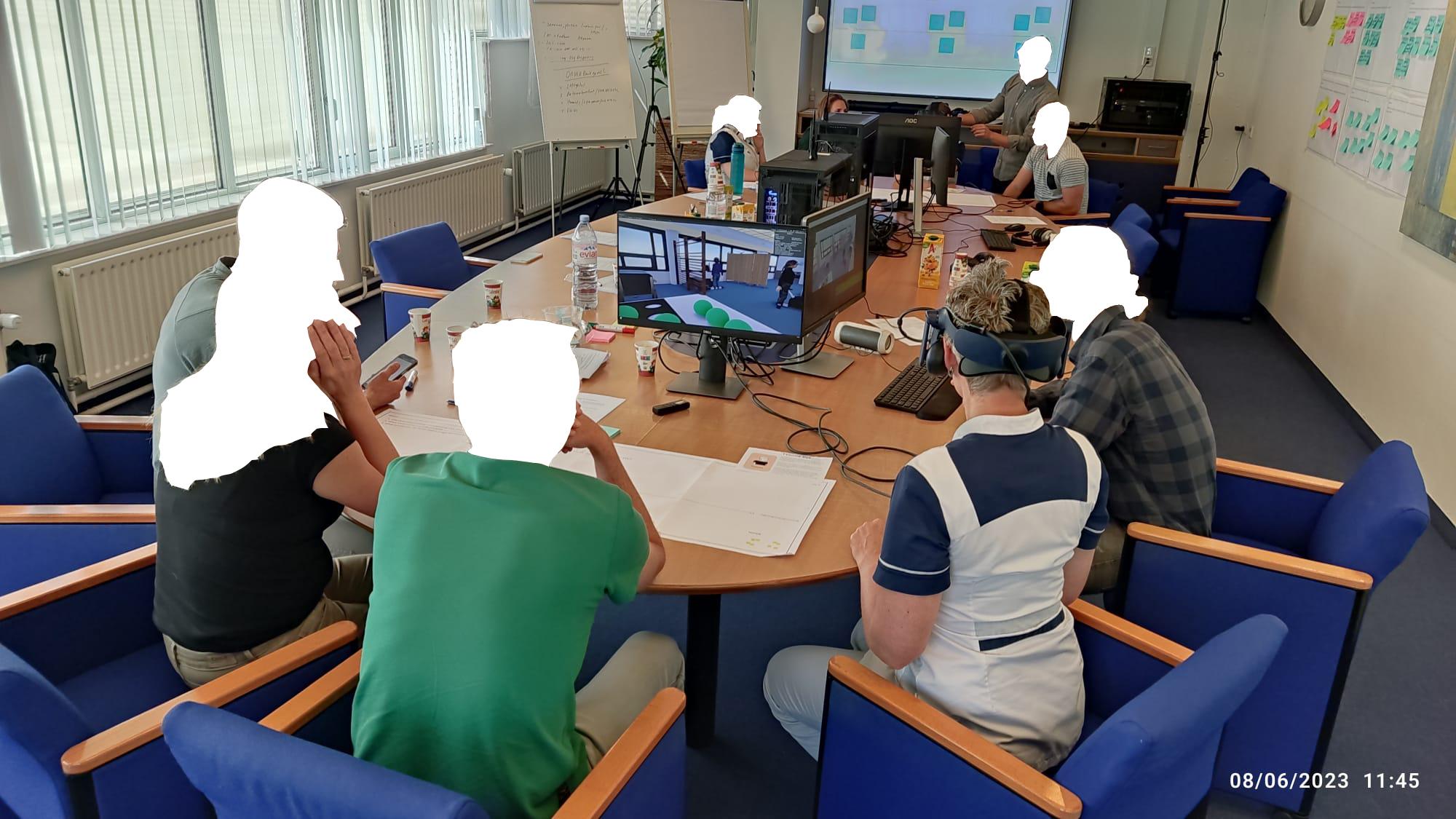** | |
| **Activity 2 - Ideation session.** Participants working on the paper sheet used during the focus group while one is wearing the HMD to explore the Immersive virtual environment. | |
| 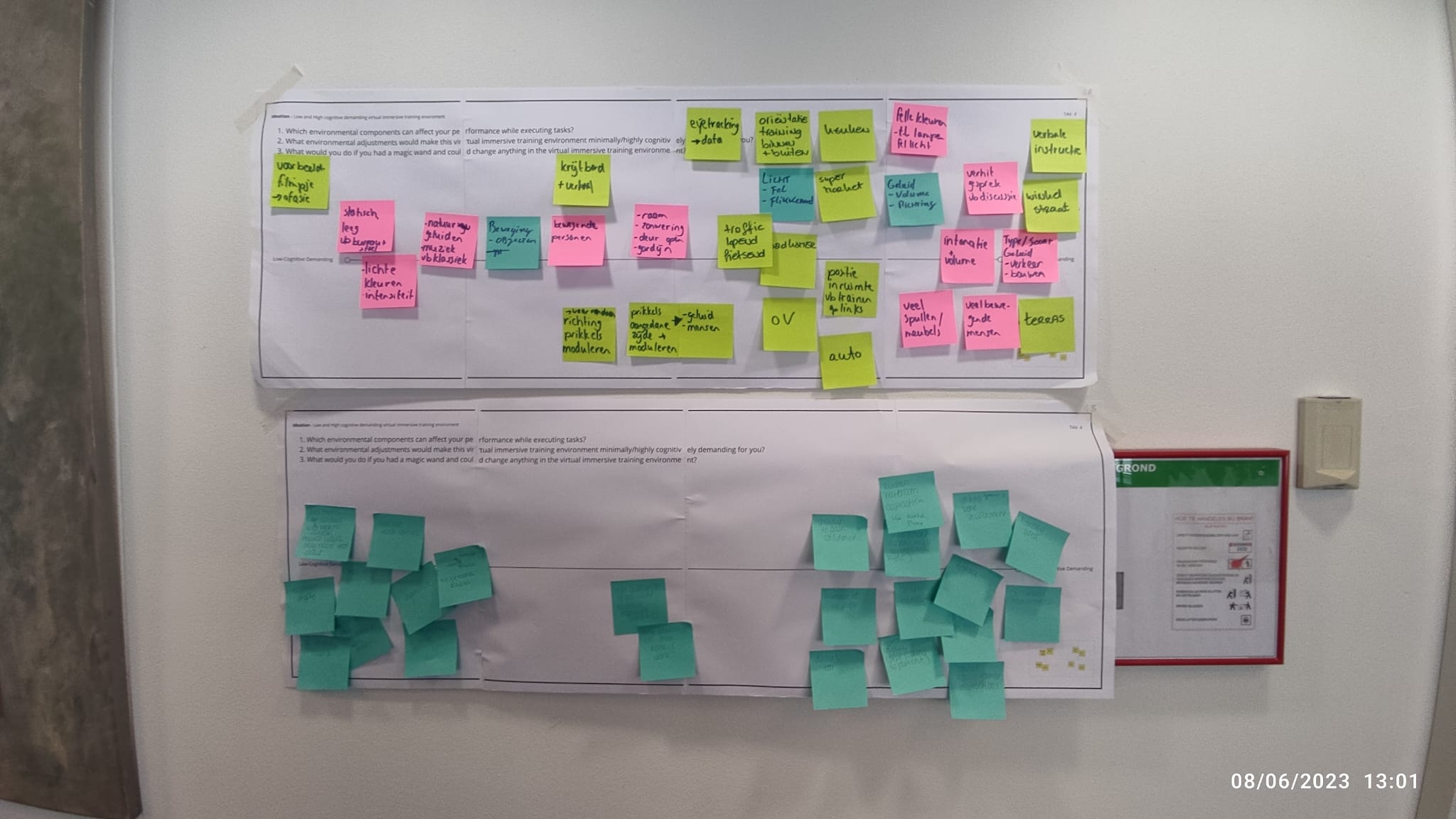 | |
| 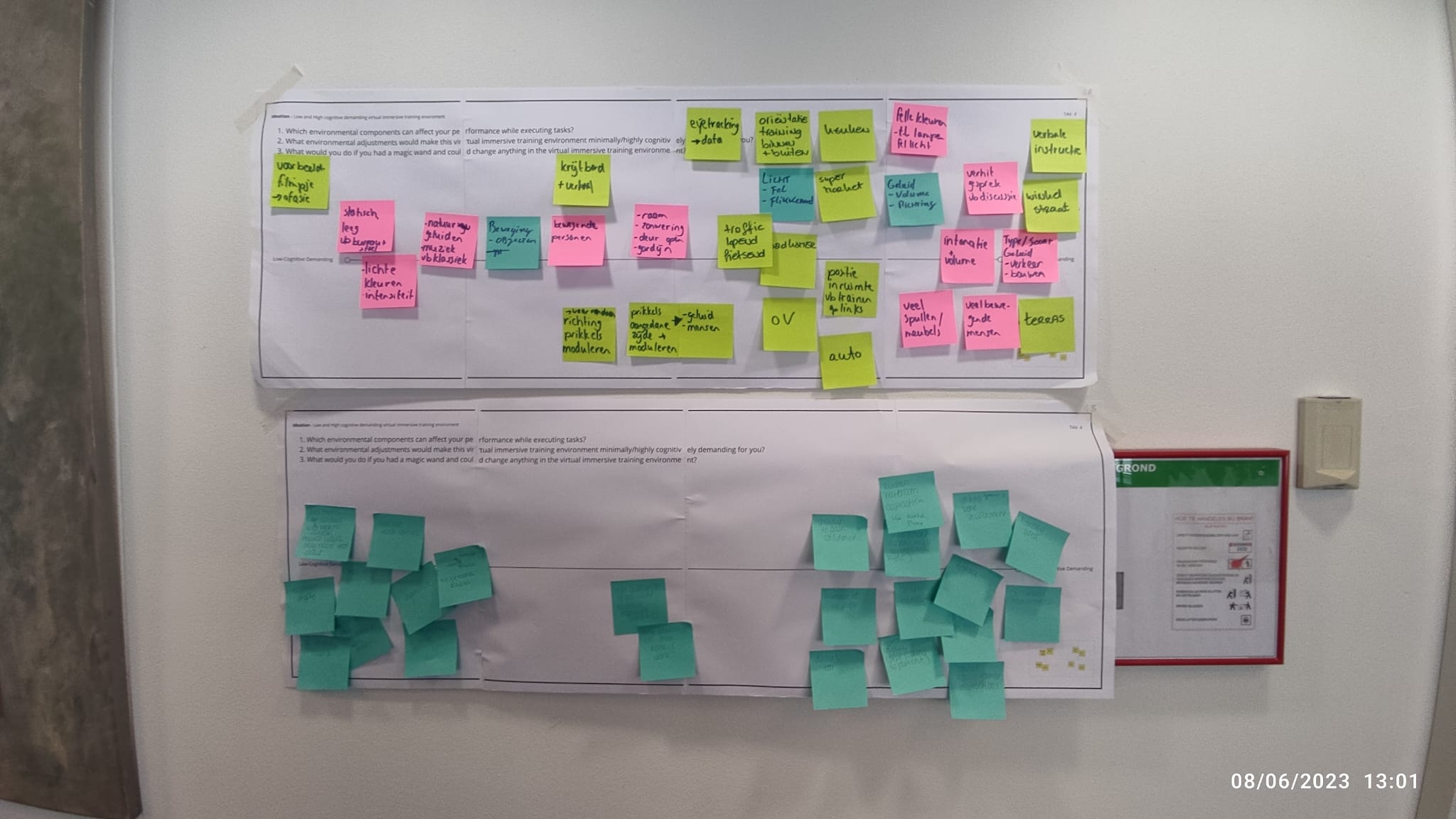 | |
| **Paper sheet.** Top: Group A, Bottom: Group B. Question 1. *Which environmental components can affect your performance while executing tasks?* Question 2. *What environmental adjustments would make this virtual immersive training environment minimally/highly cognitively demanding for you?* Question 3. *What would you do if you had a magic wand and could change anything in the virtual immersive training environment?* | |
