# Supplementary material for: Towards personalized immersive virtual reality neurorehabilitation: a human-centered design
Source: J Neuroeng Rehabil. 2025 Jan 20;22:7. doi: 10.1186/s12984-024-01489-5 (PMC11748334; doi:10.1186/s12984-024-01489-5)
Supplement: Supplementary file 2 — Additional file 2. [file 12984_2024_1489_MOESM2_ESM.pdf]

Introductie

Introduction.

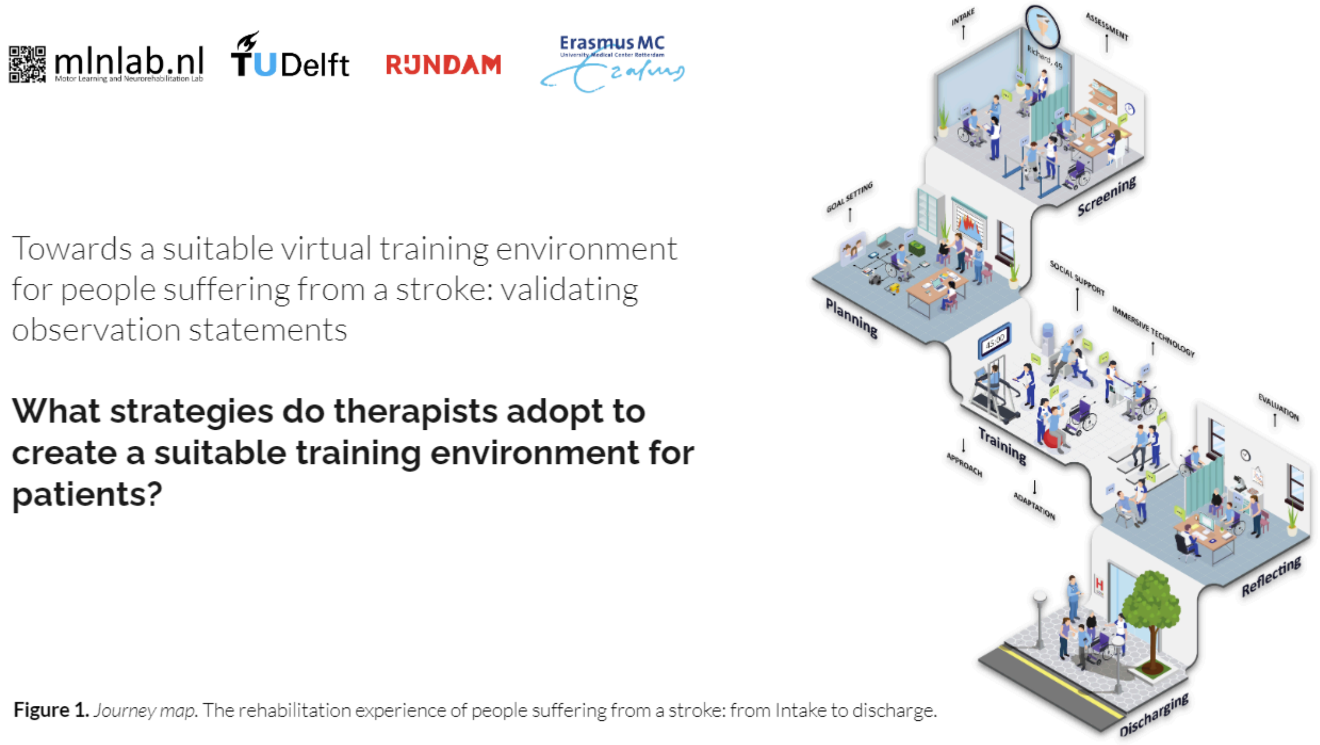

Figure 1. *Journey map.* The rehabilitation experience of people suffering from a stroke: from Intake to discharge.

Introduction.

Welkom: we zijn blij met uw deelname!

Mijn naam is Salvo Cucinella, een Ph.D. student aan de Technische Universiteit Delft; leuk om je te ontmoeten! Dit onderzoek is goed gekeurd door de Human Research Ethics Committee (HREC) van de Technische Universiteit Delft (TU Delft). Door deel te nemen, kunnen we onze observatieverklaringen valideren en klinisch relevante ontwerpbeslissingen nemen.

Wij stellen uw mening en ervaringen zeer op prijs

In deze enquête willen we beoordelen in welke mate u het eens of oneens bent met een lijst met strategieën die therapeuten gebruiken om een geschikte trainingsomgeving te creëren voor patiënten met een beroerte. U kunt elke stelling beoordelen van 1 (zeer mee oneens) tot 5 (zeer mee eens).

Data Privacy

We gebruiken Qualtrics, een beveiligd enquêtesysteem, om uw gegevens te beschermen. De TU Delft heeft een Qualtrics licentie.

Uw gegevens worden met grote zorgvuldigheid en vertrouwelijkheid behandeld. We streven ernaar om zo min mogelijk persoonsgegevens te verzamelen, zoals geslacht, leeftijd, vakgebied en ervaringsjaren.

Gegevens worden opgeslagen in een beveiligde gegevensopslag van de TU Delft met beperkte toegang voor alleen het onderzoeksteam in de TU Delft.

Gegevens zullen op geen enkele manier te herleiden zijn naar individuele deelnemers. We gebruiken pseudonimisering en geven elke respondent een label, zoals P01, P02, P03, etc.

Gepseudonimiseerde gegevens zullen worden gebruikt om onderzoeksresultaten te citeren en om vervolgonderzoek, prototyping, toekomstige publicaties en presentaties te informeren. Gepseudonimiseerde gegevens zullen openbaar worden gemaakt voor onderzoek in een in de EU gevestigde gegevensopslagplaats.

Tijd om de enquête in te vullen

Het invullen van de enquête duurt tussen de 20 en 30 minuten. Er zijn geen goede of foute antwoorden. We nodigen je uit iedere stelling toe te lichten.

Toestemming

Om de enquête te kunnen starten, kunt u op de volgende pagina aangeven of u ermee instemt dat de door u verstrekte informatie wordt gebruikt voor de bovengenoemde doeleinden. Wanneer u toestemming geeft kunt u beginnen met de enquête.

Voor vragen kunt u per e-mail contact opnemen met Salvo via S.L.Cucinella@tudelft.nl

Informed Consent

Informed Consent. **Hierbij bevestig ik dat ik toestemming geef om deel te nemen aan dit onderzoek. In verband hiermee verklaar ik het volgende:**

- Ik heb de verstrekte informatie over het onderzoek gelezen en begrepen.
- Ik ben voldoende geïnformeerd over de aard, het doel en de procedures van het onderzoek.
- Ik stem er vrijwillig mee in om deel te nemen aan deze studie en begrijp dat ik me op elk moment kan terugtrekken zonder een reden op te geven.
- Ik begrijp dat de informatie die ik verstrek zal worden gebruikt om belangrijke momenten van de activiteiten weer te geven om uit te leggen hoe het onderzoek is uitgevoerd in rapporten, publicaties, portfolio's en tijdens presentaties.
- Ik begrijp dat informatie over mijn leeftijd, geslacht, vakgebied en jarenlange professionele ervaring zal worden verzameld.
- Ik begrijp dat ik kan beslissen om mijn e-mailadres al dan niet te delen.

- Ik begrijp dat de volgende stappen zullen worden genomen om de dreiging van een datalek te minimaliseren en mijn identiteit te beschermen in het geval van een dergelijk datalek:
    - Ik ben me ervan bewust dat de verzamelde gegevens fysiek worden geborgen in de Project Storage bij de TU Delft en Git(lab)/subversion repository bij de TU Delft.
    - Ik begrijp dat verzamelde persoonlijke informatie die mij kan identificeren niet buiten het onderzoeksteam (TU Delft) zal worden gedeeld.
    - Ik ben me ervan bewust dat alle gegevens gepseudonimiseerd zullen worden.
- Ik ga ermee akkoord dat mijn antwoorden, standpunten of andere input anoniem kunnen worden geciteerd in onderzoeksresultaten.
- Ik geef toestemming om het (gepseudonimiseerde) materiaal dat in dit onderzoek is gegenereerd, openbaar beschikbaar te maken voor toekomstig onderzoek en leren in een repository zoals Zenodo.org (een in de EU gevestigde datarepository).

Gelieve aan te geven of u ermee instemt dat de door u verstrekte informatie wordt gebruikt voor de bovengenoemde doeleinden.

- ○ I give my consent
- ○ I do not give my consent

Persoonlijke informatie

Leeftijd.
Hoe oud ben jij?

[ ]

Geslacht. Wat is je geslacht?

- ○ Female
- ○ Male
- ○ [ ] Not Listed
- ○ Prefer Not to Answer

Vakgebieden. Wat is jouw vakgebied?

- ○ Physiatrist
- ○ Neuropsychologist
- ○ Psychologist
- ○ Psychodiagnostic

- Physiotherapist
- Occupational therapist
- Logopedist
- Social worker
- Nurse
- Assistant
- Researcher
- Project manager
- AIOS
- [ ] Other
- Prefer not to answer

Vakgebieden. Voel je vrij om de onderstaande ruimte te gebruiken om meer informatie toe te voegen die je specialisatie beter beschrijft (bijvoorbeeld: ik ben een kinderrevalidatiearts).

Jaar ervaring. Hoeveel jaar ervaring heeft u als professional in uw vakgebied?

Belangrijk

Belangrijk. De stellingen in deze enquête zijn het resultaat van observaties van de revalidatie van patiënten die een beroerte hebben gehad. Ze geven weer wat de onderzoekers belangrijk vinden.

We observeerden patiënten met verschillende motorische en cognitieve capaciteiten en concentreerden ons op hoe therapeuten de trainingsomgeving aanpassen, afhankelijk van de cognitieve capaciteiten van de patiënt.

Daarom verwijzen deze verklaringen niet naar een specifiek type patiënt; in plaats daarvan hebben we besloten een algemeen perspectief te behouden om de vereisten van toekomstig gepersonaliseerde oplossingen te definiëren.

Stellingen valideren

Stellingen. 1. In de voorbereiding van de trainingsomgeving dienen therapeuten rekening te houden met de fysiologische en psychologische capaciteiten van de individuele patiënt, met begrip

voor hun sociale omstandigheden (bijvoorbeeld steun van familie).

Strongly agree

○

Somewhat agree

○

Neither agree nor disagree

○

Somewhat disagree

○

Strongly disagree

○

Comment. Laat hier een toelichting achter.

Hoe pas je deze strategie toe in je dagelijkse werk?

Stellingen. 2. Tijdens de therapie moeten therapeuten de omgeving* (hoeveelheid, type kenmerken en complexiteit van de elementen) afstemmen op de voortgang en hersteldoelen van de patiënt (bijvoorbeeld autorijden).

* Grootte trainingsruimte, lichtintensiteit, schaduwen of hoeveelheid en kenmerken van tafels, stoelen, rolstoelen, trainingshulpmiddelen, deuren, ramen, pratende en bewegende mensen, enz.

Complexiteit verwijst naar de interacties tussen meerdere elementen, zoals het licht dat objecten raakt die schaduwen veroorzaken; gordijnen openen/sluiten om direct zonlicht tegen te houden of de privacy te vergroten; enzovoort.

Strongly agree

○

Somewhat agree

○

Neither agree nor disagree

○

Somewhat disagree

○

Strongly disagree

○

Comment. Laat hier een toelichting achter.

Hoe pas je deze strategie toe in je dagelijkse werk?

Stellingen. 3. Tijdens de therapie moeten therapeuten samen met de patiënten beslissen of ze de trainingsomgeving willen veranderen, afhankelijk van hun cognitieve capaciteiten om het motorisch leren te verbeteren.

Strongly agree

○

Somewhat agree

○

Neither agree nor disagree

○

Somewhat disagree

○

Strongly disagree

○

Comment. Laat hier een toelichting achter.

Hoe pas je deze strategie toe in je dagelijkse werk?

Stellingen. 4. Tijdens de therapie moeten therapeuten de niveaus van interactie* met hun patiënten aanpassen, afhankelijk van hun cognitieve capaciteiten, om het motorisch leren te verbeteren.

*afstand; fysiek contact; communicatie - terugkoppeling en inzicht geven; ruimte voor zelfreflectie.

Strongly agree

Somewhat agree

Neither agree nor disagree

Somewhat disagree

Strongly disagree

Comment. Laat hier een toelichting achter.

Hoe pas je deze strategie toe in je dagelijkse werk?

Stellingen. 5. Tijdens de therapie moeten therapeuten kiezen tussen leren door fouten en foutloze leerstrategieën om het motorisch leren te verbeteren, afhankelijk van de cognitieve capaciteiten van de patiënt.

Strongly agree

Somewhat agree

Neither agree nor disagree

Somewhat disagree

○

Strongly disagree

○

Comment. Laat hier een toelichting achter.

Hoe pas je deze strategie toe in je dagelijkse werk?

Stellingen. 6. Tijdens de therapie moeten therapeuten het gebruik van spiegels*, waardoor patiënten naar hun bewegingen kunnen kijken om het motorisch leren te verbeteren, afhankelijk laten zijn van de cognitieve capaciteiten van de patiënt.

* U kunt hierbij denken aan het gebruik van spiegels bij bijvoorbeeld spiegeltherapie of bij balanstraining e.d.

Strongly agree

○

Somewhat agree

○

Neither agree nor disagree

○

Somewhat disagree

○

Strongly disagree

○

Comment. Laat hier een toelichting achter.

Hoe pas je deze strategie toe in je dagelijkse werk?

Stellingen. 7. Tijdens de therapie moeten therapeuten met patiënten video-opnamen van eerdere trainingssessies gebruiken, afhankelijk van hun cognitieve capaciteiten, om het motorisch leren te verbeteren.

Strongly agree

Somewhat agree

Neither agree nor disagree

Somewhat disagree

Strongly disagree

Comment. Laat hier een toelichting achter.

Hoe pas je deze strategie toe in je dagelijkse werk?

Stellingen. 8. Tijdens de therapie moeten therapeuten vertrouwde elementen introduceren of vertrouwde omstandigheden* nabootsen om de motorische functies van patiënten te trainen, afhankelijk van hun cognitieve capaciteiten.

* Bijvoorbeeld: kinderen laten rondrennen terwijl een patiënt aan het oefenen is in de keuken, etc.

Strongly agree

Somewhat agree

Neither agree nor disagree

Somewhat disagree

○

Strongly disagree

○

Comment. Laat hier een toelichting achter.

Hoe pas je deze strategie toe in je dagelijkse werk?

Stellingen. 9. Tijdens de therapie moeten therapeuten ervoor kiezen om patiënten bloot te stellen aan omstandigheden die typerend zijn voor een stad of dorp - afhankelijk van de plaats waar ze wonen - om motorisch leren te verbeteren, afhankelijk van hun cognitieve capaciteiten.

Strongly agree

○

Somewhat agree

○

Neither agree nor disagree

○

Somewhat disagree

○

Strongly disagree

○

Comment. Laat hier een toelichting achter.

Hoe pas je deze strategie toe in je dagelijkse werk?

Stellingen. 10. Tijdens de therapie moeten therapeuten patiënten voorzien van werkspecifieke hulpmiddelen* - afhankelijk van hun cognitieve capaciteiten om het motorisch leren te verbeteren.

* Bijvoorbeeld het stuur dat patiënten gebruiken om opnieuw te leren fietsen, enz.

Strongly agree

Somewhat agree

Neither agree nor disagree

Somewhat disagree

Strongly disagree

Comment. Laat hier een toelichting achter.

Hoe pas je deze strategie toe in je dagelijkse werk?

Stellingen. 11. Tijdens de therapie moeten therapeuten, afhankelijk van de cognitieve capaciteiten van patiënten, kiezen of zij patiënten plaatsen in grote en volle kamers (groepssessies) of in kleine en geïsoleerde kamers (1-op-1-sessies), om motorisch leren te verbeteren.

Strongly agree

Somewhat agree

Neither agree nor disagree

Somewhat disagree

○

Strongly disagree

○

Comment. Laat hier een toelichting achter.

Hoe pas je deze strategie toe in je dagelijkse werk?

Stellingen. 12. Tijdens de therapie moeten therapeuten de blootstelling aan achtergrondlawaai of onbedoelde geluiden (bijv. stemmen, telefoonsignalen, alarmen) aanpassen aan de cognitieve capaciteiten van de patiënt, om het motorisch leren te verbeteren.

Strongly agree

○

Somewhat agree

○

Neither agree nor disagree

○

Somewhat disagree

○

Strongly disagree

○

Comment. Laat hier een toelichting achter.

Hoe pas je deze strategie toe in je dagelijkse werk?

Stellingen. 13. Tijdens de therapie moeten therapeuten de richting van het licht (spotlicht of diffuus licht) en intensiteit moduleren, afhankelijk van de cognitieve capaciteiten van de patiënt, om

het motorisch leren te verbeteren.

Strongly agree

Somewhat agree

Neither agree nor disagree

Somewhat disagree

Strongly disagree

Comment. Laat hier een toelichting achter.

Hoe pas je deze strategie toe in je dagelijkse werk?

Stellingen. 14. Tijdens de therapie moeten therapeuten het realisme van een taak* aanpassen aan de cognitieve capaciteiten van de patiënt om het motorisch leren te verbeteren.

* U kunt denken aan patiënten die boodschappen doen in een echte supermarkt of in het revalidatiecentrum etc.

Strongly agree

Somewhat agree

Neither agree nor disagree

Somewhat disagree

○

Strongly disagree

○

Comment. Laat hier een toelichting achter.

Hoe pas je deze strategie toe in je dagelijkse werk?

Stellingen. 15. Tijdens de therapie moeten therapeuten kiezen tussen unilaterale of bimanuele oefeningen, afhankelijk van de cognitieve capaciteiten van de patiënt om het motorisch leren te verbeteren.

Strongly agree

○

Somewhat agree

○

Neither agree nor disagree

○

Somewhat disagree

○

Strongly disagree

○

Comment. Laat hier een toelichting achter.

Hoe pas je deze strategie toe in je dagelijkse werk?

Stellingen. 16. Tijdens de therapie moeten therapeuten patiënten motiverende feedback geven, afhankelijk van hun cognitieve capaciteiten, om het motorisch leren te verbeteren.

Strongly agree

○

Somewhat agree

○

Neither agree nor disagree

○

Somewhat disagree

○

Strongly disagree

○

Comment. Laat hier een toelichting achter.

Hoe pas je deze strategie toe in je dagelijkse werk?

Stellingen. 17. Tijdens de therapie moeten therapeuten de stresslevels* van patiënten beïnvloeden, afhankelijk van hun cognitieve vermogens, om het motorisch leren te verbeteren.

* Bijvoorbeeld door patiënten uit hun comfortzone te halen, frustrerende activiteiten te creëren zoals patiënten in competitie brengen met anderen, enz.

Strongly agree

○

Somewhat agree

○

Neither agree nor disagree

○

Somewhat disagree

○

Strongly disagree

○

Comment. Laat hier een toelichting achter.

Hoe pas je deze strategie toe in je dagelijkse werk?

Stellingen. 18. Tijdens de therapie moeten therapeuten kiezen uit mondelinge, gebaren- of schriftelijke instructies, aangepast aan de cognitieve capaciteiten van de patiënt om het motorisch leren te verbeteren.

Strongly agree

○

Somewhat agree

○

Neither agree nor disagree

○

Somewhat disagree

○

Strongly disagree

○

Comment. Laat hier een toelichting achter.

Hoe pas je deze strategie toe in je dagelijkse werk?

Stellingen. 19. Tijdens de therapie moeten therapeuten familieleden van de patiënt erbij betrekken, afhankelijk van de cognitieve capaciteiten van de patiënt, om het motorisch leren te

verbeteren.

Strongly agree

○

Somewhat agree

○

Neither agree nor disagree

○

Somewhat disagree

○

Strongly disagree

○

Comment. Laat hier een toelichting achter.

Hoe pas je deze strategie toe in je dagelijkse werk?

Stellingen. 20. Tijdens de therapie moeten therapeuten ervoor kiezen om patiënten secundaire taken te geven, afhankelijk van hun cognitieve capaciteiten, om het motorisch leren te verbeteren.

* Praat bijvoorbeeld al wandelend over het dagelijks leven

Strongly agree

○

Somewhat agree

○

Neither agree nor disagree

○

Somewhat disagree

○

Strongly disagree

○

Comment. Laat hier een toelichting achter.

Hoe pas je deze strategie toe in je dagelijkse werk?

Stellingen. 21. Tijdens de therapie moeten therapeuten patiënten laten communiceren met andere mensen, afhankelijk van hun cognitieve capaciteiten om het motorisch leren te verbeteren.

Strongly agree

○

Somewhat agree

○

Neither agree nor disagree

○

Somewhat disagree

○

Strongly disagree

○

Comment. Laat hier een toelichting achter.

Hoe pas je deze strategie toe in je dagelijkse werk?

Stellingen. 22. Tijdens de therapie moeten therapeuten kiezen om de bewegingen van de minder aangedane arm van de patiënt te beperken om de meer aangedane arm te trainen, afhankelijk van hun cognitieve capaciteiten om het motorisch leren te verbeteren.

Strongly agree

○

Somewhat agree

○

Neither agree nor disagree

○

Somewhat disagree

○

Strongly disagree

○

Comment. Laat hier een toelichting achter.

Hoe pas je deze strategie toe in je dagelijkse werk?

Jouw mening

Stellingen. **Stellingen**

1. In de voorbereiding van de trainingsomgeving dienen therapeuten rekening te houden met de fysiologische en psychologische capaciteiten van de individuele patiënt, met begrip voor hun sociale omstandigheden (bijvoorbeeld steun van familie).
2. Tijdens de therapie moeten therapeuten de omgeving (hoeveelheid, type kenmerken en complexiteit van de elementen) afstemmen op de voortgang en hersteldoelen van de patiënt (bijvoorbeeld autorijden).
3. Tijdens de therapie moeten therapeuten samen met de patiënten beslissen of ze de trainingsomgeving willen veranderen, afhankelijk van hun cognitieve capaciteiten om het motorisch leren te verbeteren.
4. Tijdens de therapie moeten therapeuten de niveaus van interactie met hun patiënten aanpassen, afhankelijk van hun cognitieve capaciteiten, om het motorisch leren te verbeteren.
5. Tijdens de therapie moeten therapeuten kiezen tussen leren door fouten en foutloze leerstrategieën om het motorisch leren te verbeteren, afhankelijk van de cognitieve

capaciteiten van de patiënt.

6. Tijdens de therapie moeten therapeuten het gebruik van spiegels, waardoor patiënten naar hun bewegingen kunnen kijken om het motorisch leren te verbeteren, afhankelijk laten zijn van de cognitieve capaciteiten van de patiënt.
7. Tijdens de therapie moeten therapeuten met patiënten video-opnamen van eerdere trainingssessies gebruiken, afhankelijk van hun cognitieve capaciteiten, om het motorisch leren te verbeteren.
8. Tijdens de therapie moeten therapeuten vertrouwde elementen introduceren of vertrouwde omstandigheden* nabootsen om de motorische functies van patiënten te trainen, afhankelijk van hun cognitieve capaciteiten.
9. Tijdens de therapie moeten therapeuten ervoor kiezen om patiënten bloot te stellen aan omstandigheden die typerend zijn voor een stad of dorp - afhankelijk van de plaats waar ze wonen - om motorisch leren te verbeteren, afhankelijk van hun cognitieve capaciteiten.
10. Tijdens de therapie moeten therapeuten patiënten voorzien van werkspecifieke hulpmiddelen - afhankelijk van hun cognitieve capaciteiten om het motorisch leren te verbeteren.
11. Tijdens de therapie moeten therapeuten, afhankelijk van de cognitieve capaciteiten van patiënten, kiezen of zij patiënten plaatsen in grote en volle kamers (groepssessies) of in kleine en geïsoleerde kamers (1-op-1-sessies), om motorisch leren te verbeteren.
12. Tijdens de therapie moeten therapeuten de blootstelling aan achtergrondlawaai of onbedoelde geluiden (bijv. stemmen, telefoonsignalen, alarmen) aanpassen aan de cognitieve capaciteiten van de patiënt, om het motorisch leren te verbeteren.
13. Tijdens de therapie moeten therapeuten de richting van het licht (spotlicht of diffuus licht) en intensiteit moduleren, afhankelijk van de cognitieve capaciteiten van de patiënt, om het motorisch leren te verbeteren.
14. Tijdens de therapie moeten therapeuten het realisme van een taak aanpassen aan de cognitieve capaciteiten van de patiënt om het motorisch leren te verbeteren.
15. Tijdens de therapie moeten therapeuten kiezen tussen unilaterale of bimanuele oefeningen, afhankelijk van de cognitieve capaciteiten van de patiënt om het motorisch leren te verbeteren.
16. Tijdens de therapie moeten therapeuten patiënten motiverende feedback geven, afhankelijk van hun cognitieve capaciteiten, om het motorisch leren te verbeteren.
17. Tijdens de therapie moeten therapeuten de stresslevels van patiënten beïnvloeden, afhankelijk van hun cognitieve vermogens, om het motorisch leren te verbeteren.
18. Tijdens de therapie moeten therapeuten kiezen uit mondelinge, gebaren- of schriftelijke instructies, aangepast aan de cognitieve capaciteiten van de patiënt om het motorisch leren te verbeteren.
19. Tijdens de therapie moeten therapeuten familieleden van de patiënt erbij betrekken, afhankelijk van de cognitieve capaciteiten van de patiënt, om het motorisch leren te

verbeteren.

20. Tijdens de therapie moeten therapeuten ervoor kiezen om patiënten secundaire taken te geven, afhankelijk van hun cognitieve capaciteiten, om het motorisch leren te verbeteren.
21. Tijdens de therapie moeten therapeuten patiënten laten communiceren met andere mensen, afhankelijk van hun cognitieve capaciteiten om het motorisch leren te verbeteren.
22. Tijdens de therapie moeten therapeuten kiezen om de bewegingen van de minder aangedane arm van de patiënt te beperken om de meer aangedane arm te trainen, afhankelijk van hun cognitieve capaciteiten om het motorisch leren te verbeteren.

Jouw mening. Wat vind je van de stellingen?

Fijn als je je antwoord verder toe kan lichten.

Jouw mening. Wat is volgens jouw de meest relevante stelling?

Fijn als je je antwoord verder toe kan lichten.

Jouw mening. Wat is volgens jouw de minst relevant stelling?

Fijn als je je antwoord verder toe kan lichten.

Jouw mening. Wat zou je willen veranderen in deze stellingen?

Fijn als je je antwoord verder toe kan lichten.

Jouw mening. Wat miste je in deze stellingen?

Fijn als je je antwoord verder toe kan lichten.

Jouw mening. Hoe pas je de benoemde strategieën toe in je therapie?

Kunt u uw antwoord toelichten door het geven van een voorbeeld?

Voorbeelden:

- Tijdens de therapie verplaats ik de patiënt naar een kleine kamer waar alleen ik aanwezig ben als ik merk dat ze de gegeven motorische taak niet goed kunnen uitvoeren.

- Tijdens de therapie sluit ik de ramen en de gordijnen om de patiënt te laten focussen op de gegeven motorische taak.
- Tijdens de therapie plaats ik de patiënt voor een spiegel om de uitvoering van de gegeven motorische taak te vergemakkelijken.

Jouw mening. Welke andere strategieën passen jij of jouw collega's toe? En welke strategieën ken je verder?

Fijn als je je antwoord verder toe kan lichten.

Vervolg

Preference. Ik wil deelnemen aan een vervolgsessie om samen een virtuele trainingsomgeving te creëren voor mensen met een beroerte.

- No
- Yes, my email address is ...

Preference. Ik wil de mogelijkheid behouden om mijn toestemming voor het onderzoek in te trekken.

- No
- Yes, my email address is ...

Powered by Qualtrics
