# Supplementary material for: Towards personalized immersive virtual reality neurorehabilitation: a human-centered design
Source: J Neuroeng Rehabil. 2025 Jan 20;22:7. doi: 10.1186/s12984-024-01489-5 (PMC11748334; doi:10.1186/s12984-024-01489-5)
Supplement: Supplementary file 3 — Additional file 3. [file 12984_2024_1489_MOESM3_ESM.docx]

**Additional files**

**Additional file 3.** Study 4. Pictures of the paper sheets used and filled in by participants with Pot-its reporting their answers.

| **Group A** | **Group B** |
| --- | --- |
| **Study 4 - activity 1: focus group** | |
| 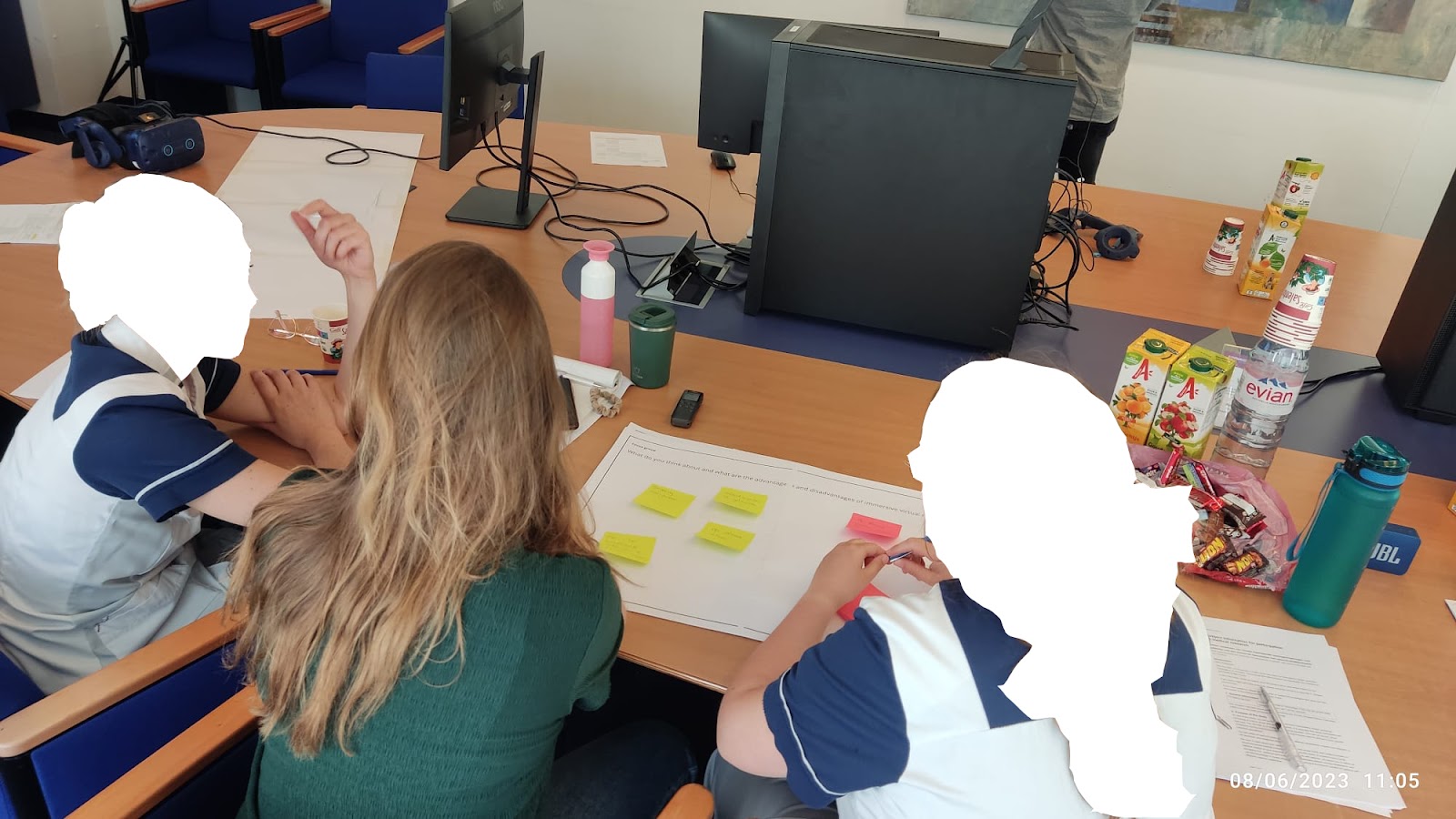 | |
| **Activity 1 - Focus group.** Participants working on the paper sheet used during the focus group. Post-its placed on top of the paper sheet report participants’ ideas. | |
| 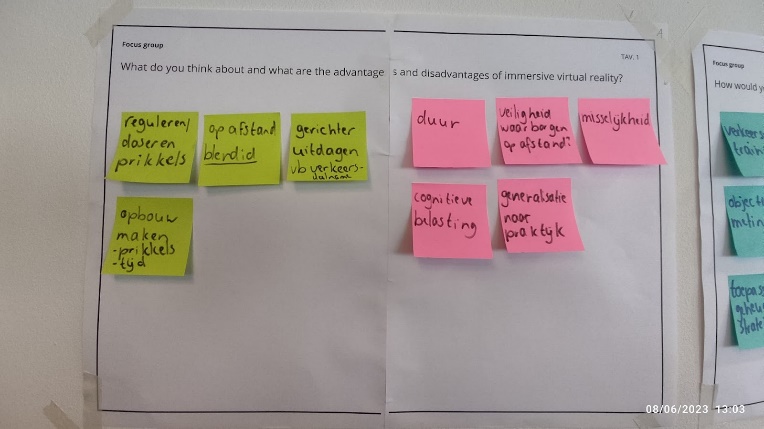 | 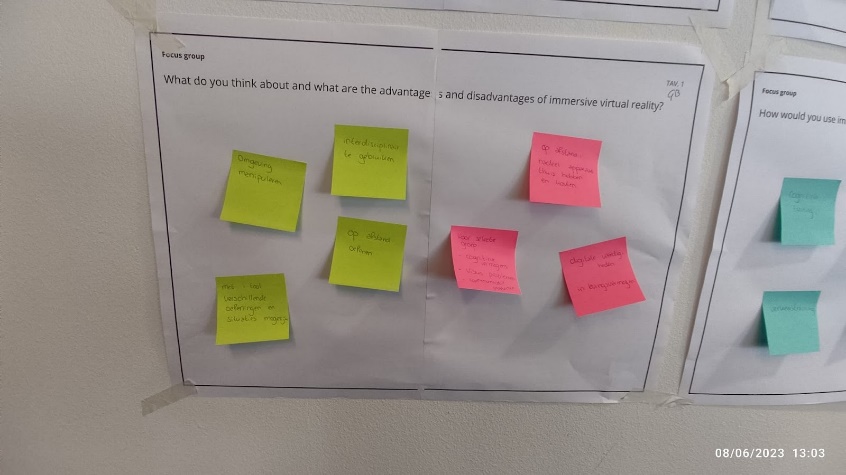 |
| **Paper sheet 1.** Question 1. *What do you think about and what are the advantages and disadvantages of immersive virtual reality?* | |
| 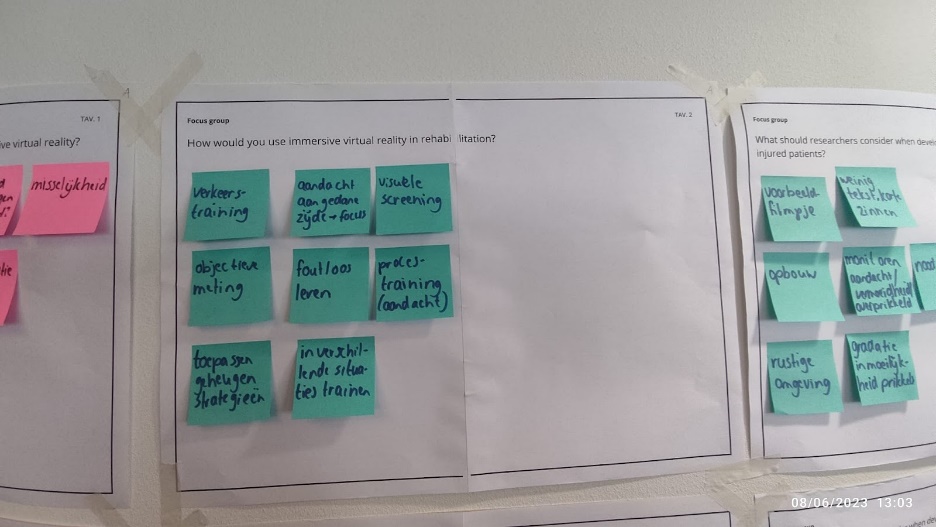 | 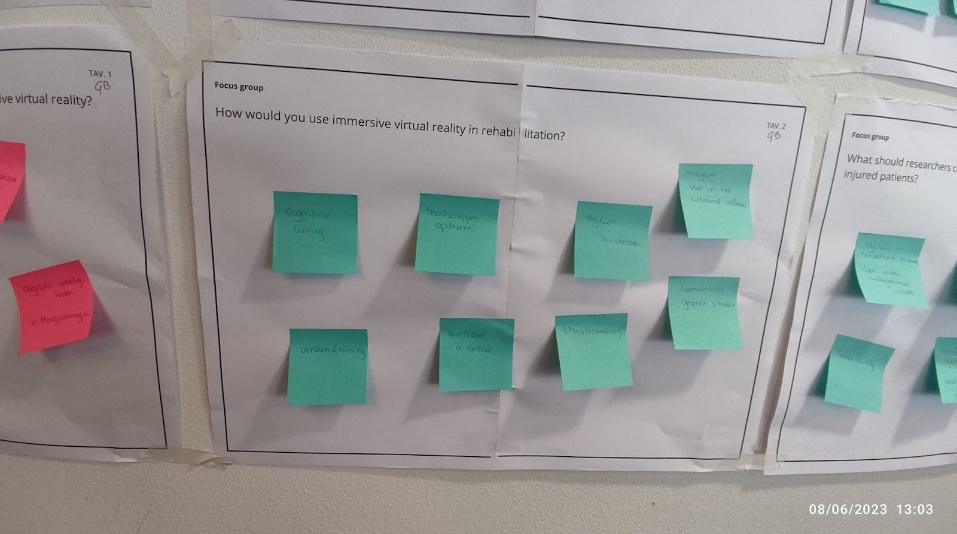 |
| **Paper sheet 2.** Question 2. *How would you use immersive virtual reality in rehabilitation?* | |
| 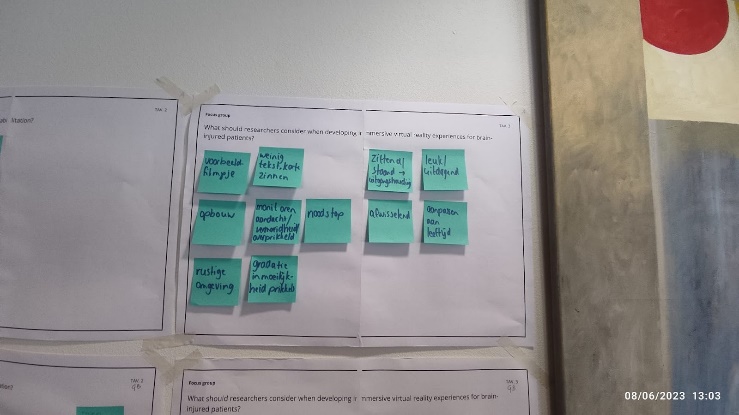 | 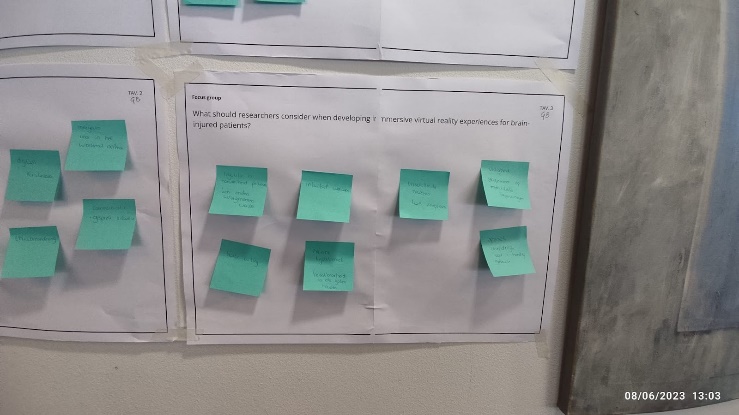 |
| **Paper sheet 3.** Question 3. *What should researchers consider when developing immersive virtual reality experiences for brain-injured patients?* | |

| **Study 4 - activity 2: ideation session** |
| --- |
| **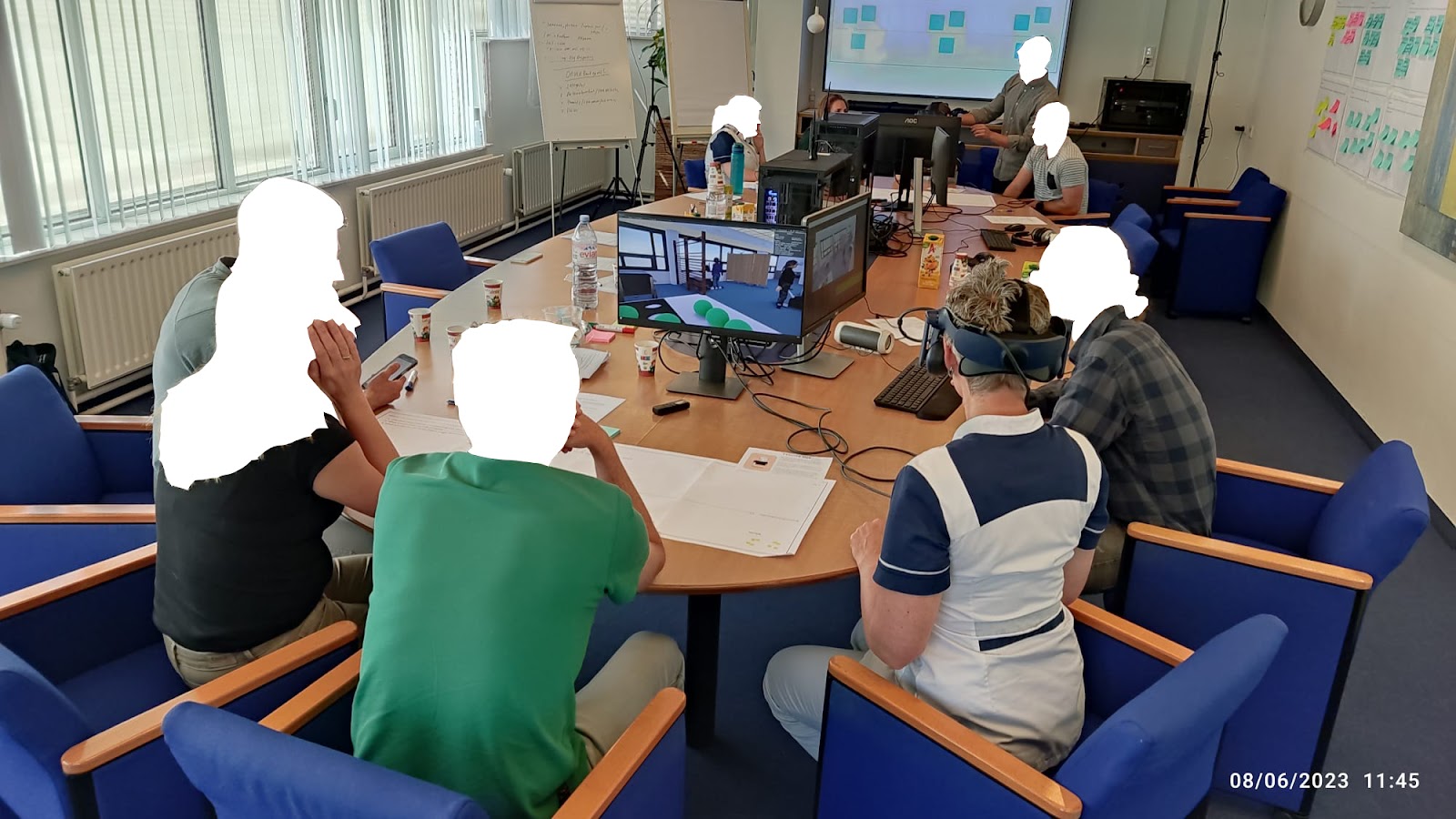** |
| **Activity 2 - Ideation session.** Participants working on the paper sheet used during the focus group while one is wearing the HMD to explore the immersive virtual environment. |
| 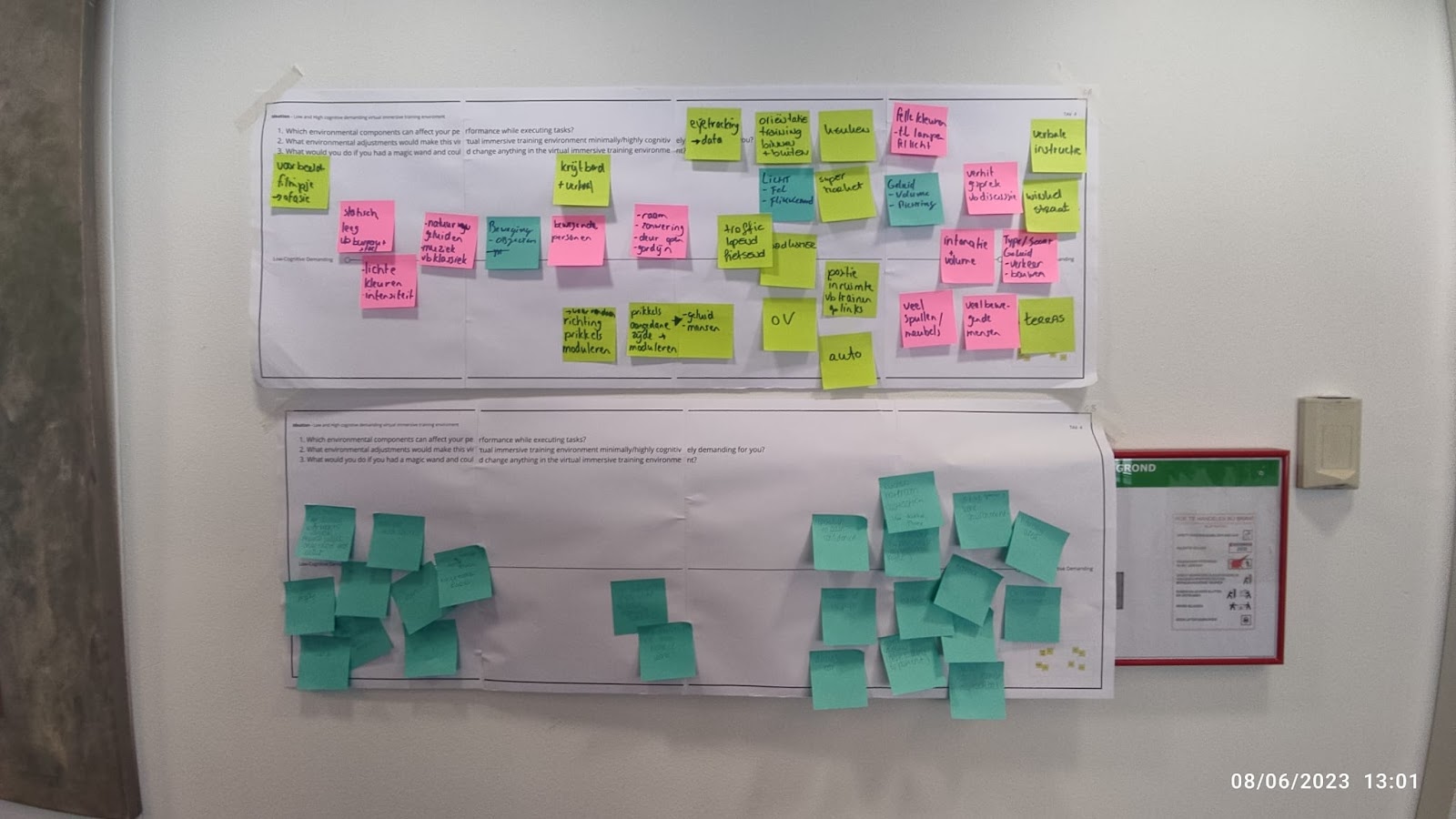 |
| 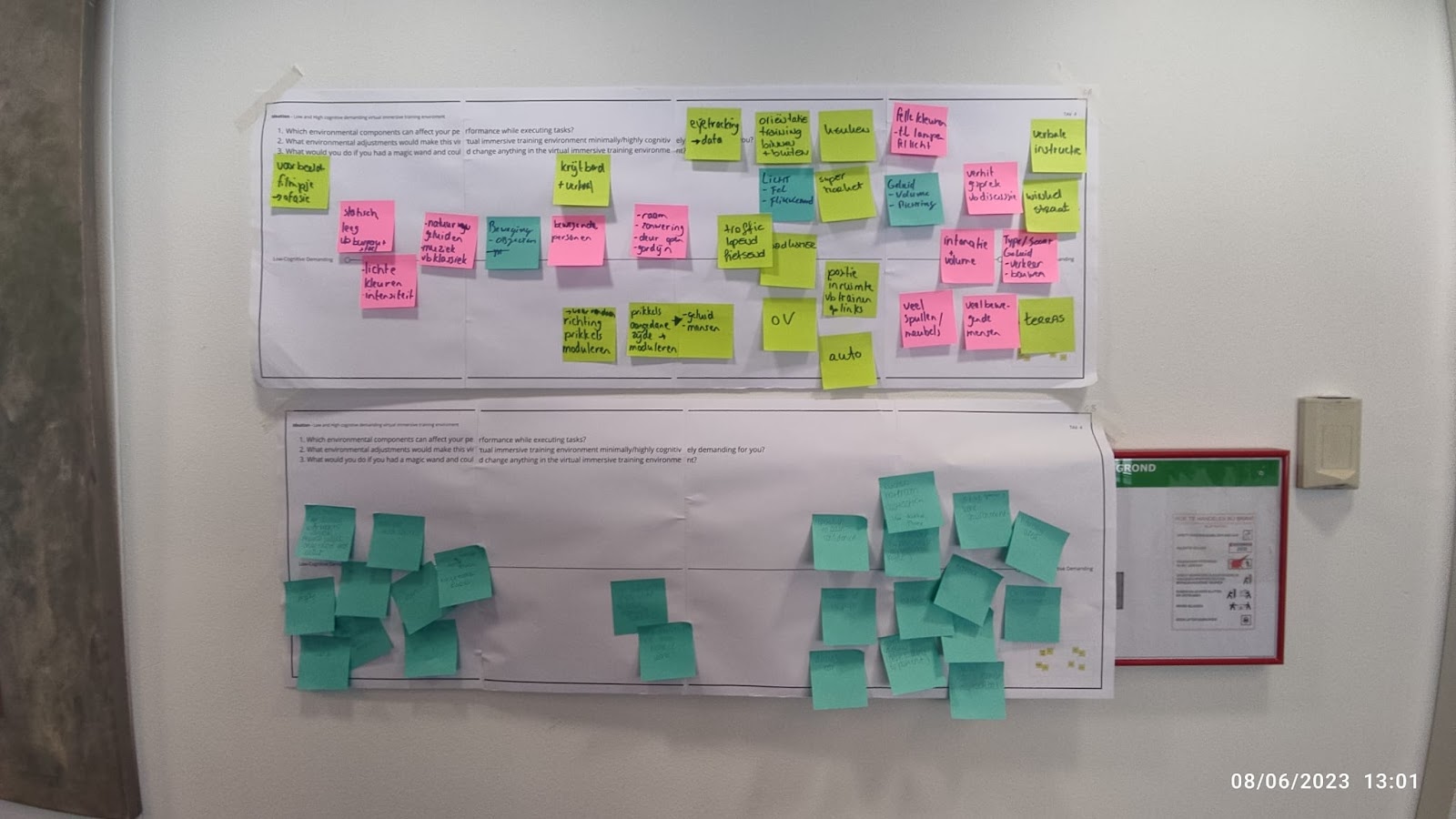 |
| **Paper sheet.** Top: Group A, Bottom: Group B. Question 1. *Which environmental components can affect your performance while executing tasks?* Question 2. *What environmental adjustments would make this virtual immersive training environment minimally/highly cognitively demanding for you?* Question 3. *What would you do if you had a magic wand and could change anything in the virtual immersive training environment?* |
